# Supplementary material for: Genomic Insights into ARR Genes: Key Role in Cotton Leaf Abscission Formation
Source: Int J Mol Sci. 2025 Jul 24;26(15):7161. doi: 10.3390/ijms26157161 (PMC12346069; doi:10.3390/ijms26157161)
Supplement: Supplementary file 1 [file ijms-26-07161-s001.zip › Additional file S1.pdf]

The protein sequences of the *ARR* proteins from cotton species and *Arabidopsis thaliana*.

G. arboretum

>GaARR1
MESKELNLNKELKAGGAGNGFIDRSKVRILLCDNDTKSCEEVFSLLLKCSYQVTTVRSARQVI
DALNAEGPDIDIILTEVDLPMTKGMKLLKYIMRNNELRRIPVIMMSAQDEVSIVVKCLRLGAA
DYLVKPLRTNELLNLWTHMWRRRRELGLSEKNILNCDFDLVASDPSDANTNSTTLFSDDTDE
RSRKSSNPEMGISTHQEDESAAATVEPPQSDSPECRPDVPGISDRRTGQFSSVPKKSELKIGESS
AFFTYVKSSAVKTSSTQVATPNHESAAENKIGEEHLPQPGEQVVSDTRVHENGETWENNSQG
DEFRSSSSVPDSLSLERSSTPASMEFSQQRDFKEDKFSPALVPPSNETQHDVSGLPTQSAYLHY
MPGVLNQVMMPSSTQLFQNNLHDIHNHTSSPLVPQYNHLQQCLPHPHVSGMASFPYYPVNM
CMQPGQMPTGHSWPSFGNSSSNEVQPSKVDRREAALIKFRQKRKERCFDKKIRYVNRKRLAE
RRPRVRGQFVRKNGATVDLNGQPASADYDEDEEEQASRDSSPEDDTSGC
>GaARR2
MNSSNGNGSMSTASSSGAWNGRDQFPAGLRVLVVDDDQTCLMVFEKMLKTCLYQVTKCNK
AETALSVLREHKNEFDIVISDVHMPDMDGFELLKHINSEMDLPVILISADARKQVVMKGVTYG
ACDYLIKPVRIETLKNIWQHVVRKKARNTF
>GaARR3
MKNSIGGKGSMSTASSITTWKAGDVVSVPDQFPAGLRVLVVDDDPTCLIILEKMLRNCSYDVT
KCNRAETALLKLRENRNGFDIVISDVHMPDMDGFKLLEHIGLEMDLPVIMMSADDGKDVVM
KGVTHGACDYLIKPVRIEALKNIWQHVVRKRKNEWKDLEQSGSVEEGDRQPKQSEDTDYSSS
VNEGNWKSSKKRKDDEDETDDRDDTSTLKKPRVVWSVELHQQFVAAVNQLGIDKAVPKKIL
ELMNVPGLTRENVASHLQKYRLYLRRLSGVSPHSCNLNNSFMNPQDETFGPLSSVHGFDLQTL
TATGQLPAQCLATLQAAVLGRSTAKSSIPMPLVNQRNIFSFENPKLRFGEGQQQHVNNNNKQV
NLLHGIPTAMESKQLTSLRHTSQSIGNLNMQVAPHGAQSSQNNSSLIEMGQPLSRVQILNDSTV
PLSVGQPIVPNGIAANVSTRNGIPENIRAPGYNLVSQTSSILNFPMNHASELPVDTFSLRSTPGMS
DHTSKGAFHEDFNSEIKGSGGFLPSYDVFNDYQYKSQNWELQNAGMILDDSQHSNSLQGNLD
LTQSVLVQQGFPSGQINGQNRSVPIVSKAMFSAGDSTEPGNLLNVNHHLNTIRADNTVRVKSE
SVADGNPSNLFTDHFGQEDLMIALLKQQQGIAPVENEFDFDGYSLDNIPV
>GaARR4
MVLAESGFSSPRNDAFPAGLRVLVVDDDPTWLKILEKMLKKCSYEVTTCCLAREALNLLRER
KDGYDIVISDVNMPDMDGFKLLEHVGLEMDLPVIMMSVDGETSRVMKGVQHGACDYLLKPI
RMKELRNIWQHVFRKKIHEVRDIESLEGFESIQMTRSGYDLFDDGHFLSGDDTTSGRKRKDAD
NKHDDRELSDPSSTKKARVVWTVDLHQKFVKAVNQIGFDKVGPKKILDLMNVPWLTRENVA
SHLQKYRLYLSRLQKESDIKNSFIGMKHSDLPSKDSTASFGPHKAMNMIPDDVPNSTYSFSASN
SQAQNVDLKGQGDLKGITSAPMAEPKGALSVDIRDSHEAKSTQMSFDHSLGSVDSAVSFASFN
STTPLQYPWTEIPEIQFKQECESLHLENGFSQLPLPGPSMIENEANRSRIEVKPLLDECRSNFVEH
LGPVGAEDLFPIQSKSQSLNNQVFDLISATKSSMKTQDVGLNYLADSEFALRNLNASGVGVPL
ATLSEDLQICWLQGDCYPMNYGLQDLECSTYDNPALMAETPFHLYDVLRFDHEHLFDPAEYY
AIDQGLFA
>GaARR5
MATMHRVVQSSVSTSDATTTSYGGLTSCKAADIVISDQFPAGLRVLVVDDDITCLKILEQMLH
RCRYHVTTCPQAKVALNLLRERKGCFDVILSDVYMPDMDGYKLLEHVGLEMDLPVIMMSAD
GSTRAVMKGIRHGACDYLIKPIREEELKNIWQHVVRKKWNENKELEHSGSLDDTDQHKRGHD

DAEYGSSANDATDTSLKPLKKRSNSKEGDNGEIDNDDPSTSKKPRVVWSVELHQQFVSAVNQ
LGIDKAVPKRILELMNVPGLTRENVASHLQKFRLYLKRISGVAQQGGIANPLCGPVEAKVKIGS
LGSFNIQPLAASGQIPPQTLAALHAELLGRPAGNLVVAMDQSALLQATPHGPKCIQVDHGVAF
IQDLVKSESSSSKHFSQSFAPVEDVASGFRSWPSNNIDTAGPSNSGGLSTQNGNMLIDLLQQQQ
QLQKPQQRSTVSELRRSINVQPSCHVVPSQSSASFQAGNSPVSVTQNGSYSRTAVIDYNFLSSQ
SNCPSLNIGQVSDVTLQTTGVLSGYIPLASVSPSVSSCSVNADNCASQQVQTSSITFKASRRLPG
FVHSTSNIPDPYGSTKSGDLLNQEPFNNLGYINKGTCLPAKFAVDEFQSHLSSSSHGKVFCENIG
TRVKQEPSMEFGDNAKVGIPMLQQFPPNDLMSVFTE
>GaARR6
MGEVVVTSEEVEEVKIDSETEEQENGEVEGNMRRTKKKKKNKKPGCSASGAVVNWERFLPM
PALRVLLVEADDSTRQIISALLRKCSYRVAAVPDGLKAWEMLKGRPHNVDLILTEVDLPSISGF
ALLTLIMEHDIFKSIPVIMMSSQDSVSTVYKCMLRGAADYLVKPIRRNELRNLWQHVWRRQSS
IVGGNSPRDESIGQKKVETTSENNAASNHSIGCLDGVGKNKEQTEKGSDAQSSCTKPEMEAES
AQKENMQEFSHLIKVNSLPIESQKHEAHGSFNQNLLMHEMETEVVDSCKDAYTTLYKGVELE
NQRRDTRVLVEAGDALVESPREAIDFMGTFNKNCTSSSINSAKKFDSSLYLDLSLRRSNPNVFE
NQVTQERPTLWHPSSSAFTRYTSRVSHPLHSTSMSFVDQKKDSETNSEKMLTNIMSENNSDTP
SPTLTSQRNTNSLTIGATVELKQTEVATPCTQHRLFPVPLPVKGIRLNNPCNGYNTIIPPIFCARS
SSSTAPSPSTANQQEPAFRVNLFRHSSFEVNSSGQSYDRLASNTKQSTSQPLQKLDQKLDSIEDR
GHISPTTDQSASSSFCNGSLSQLNGVAYGSTGASNGNVDQVAVTRASTESKNDDSLSSPSGKPC
RSIQREAALMKFRLKRKDRCFEKKVRYESRKKLAEQRPRVKGQFVRQVQADPMHTDTEHHY
GNSSDG
>GaARR7
MGNMATQTQFHVLAVDDSLIDRKLIERLLKTSSYQVTAVDSGSKALEFLGLNGENEDEERNSS
VESVSAADEDHQVGVNLIITDYCMPGMTGYDLLRKIKQSSSFKDIPVVIMSSENIPSRINRCLED
GAEEFFLKPVKLSDVNKLRPHLMKGITTKTEMQSNTNKRKGSEEIQSPDRTRPRYNELEVV
>GaARR8
MGIVQMNNNGPVAGGWVELNTHICDENTNIRDGVTGEGQGLSDEDESRINEDVENRNNGKK
AVVQLRGPLVCWERFLPLRSLKVLLVENDDSTRHVVCALLRNCGYEVTAVSNGLLAWKILED
LTNHIDLVLSEVVMPCLSGIGLLCKIMNHKTRKNIPVIMMSSHDSMNIVLKCLSKGAVDFLVKP
IRKNELKNLWQHVWRKCHSSSCSGSGSGIQTQKSTKSKSGGSDNNTGNNEEDDIGSVGLNAQ
DRSDNGSGTQSSWTKRAIEVDSSQPISARDQVMHSRSEVLGNSWVPVTITRECDSRDDELDNA
VKGKDLEIGVPKITALQLENPSEKVNTIAAGGNQEKLSELNPSKDDEKLEKAQLELTGEKPGV
DLVNRAADVIGVISKNTDAQIESAVFDIPDSLPKVSDTKGKVIYKTKEMPSLVLSLKRLIDVGD
SGTSAHERNVLRHSDLSAFSRYNSGSTANQAPIGNVGSCSPLDNSLEATNTDSMKNFHSNSNN
MPPNQQSNGSSNNNDMGSTTNNAFSKSAVLNDKPASKTSGPSSAFQPVQKGHATAMQSPAED
KADAAIGKKILAKAKGTDQQVQVQHHHHHYYHYHHHVHKMPQNQKLDNQDDLCQCGSSN
MSSAPHVEANAGNHSSNGSAPESNHGSNGQNGNITALNSRGLNLESENGLLGKGGTVGGIGF
GGSNGADQNRFSQREAALNKFRQKRKERCFEKKVRYQSRKKLAEQRPRIRGQFVRQVPENKN
KDTNC
>GaARR9
MARNGGVAWMRTTEKIDGYDLSTSDTEEVHVLAVDDSLVDRKVIERLLRISSCKVTAVDSGR
RALQYLGLDEEIQKKETNGFDGLKVDLIITDYCMPGMTGYELLKKVKESSAFREIPVVIMSSEN
VIARIDRCLEEGAEDFIVKPVKLSDVKRIIDYTTTELREGEQREGARARRRGINKRKQREGDDD
LSSSPPSTLSSSTSSSPSSSIQSATAPSSPSTLDSPTRRLKMTSSE

>GaARR10
MTLQEKRGGSNGEDGGKDRFPIGMHVLAVDDDPVCLKVLENLLRKCQYHVSTTNQATTALK
MLRENRNRYDLVITGVNMPDMDAFKLLELVGLEMDLPVIMLSTHGDTELVMKGITHGACDY
LLKPVRIEELKNIWQHVVRKNKPDFKDHINALNQDNGHEDEDRSIQEKPRVVWSDEVHRKFV
SAVNELGLDKAVPKKVLDLMNVEGLKRETVASHLQKYRLYIGHSIVATLGSKDPSYLRMGPL
GGFGYFHTLTGPGRISSASLPSYQPGGMFGRLNTSATLSLHGISSSVIQPGHCQTSNNPINGSGKI
QPAVVPANQKKNGTLFQGIPTSVDLNQPSQNKPTNGCGEFNRVNDPNFQDARMAVGGSCNTL
HVSSGNPLLLQSNTQQTKHSGAFGNQASLSNSILDLCSFKAKSANLEDSKGDISNVGLNNYIIL
NMDYATKQQRGDSRHDNNGNTNHSFCRADSLVPFLFVCFVLFCCVYKICQKTYDAWC
>GaARR11
MTMEEKLGGSNGEDGGRDRFPIGMRVLAVDDDPICLKVLENLLRKCQYHVTTTNQAITALKM
LRENRNRYDLVISDVNMPDMDGFKLLELVGLEMDLPVIMLSAHSDTKLVMKGITHGACDYLL
KPVRIEELKNIWQHVVRKKKPDSKDQINASNQDKSRGGTGETGPTSSSSDQKVSKKRKDQSED
EDEEGDDNGHEDPSTQKKPRVVWSVELHRKFVSAVNQLGLEKAVPKKILDLMNVEGLTREN
VASHLQKYRLYLKRLSSVATQQANMVAALGSKDPSYLRMGSLDGFGDFRTLTGPGRISSASLS
SYQPGGLFGRLNSSAALSLRGISSGVIQSGHSQTLSNPINGFGKIQPAVVPANQNQNGTLFQGIP
TSINQLSQNKPTNHFGEFNRGNDPNAFGVATNFQDVRVTVGGSSNTLPVSSGNPLLLQSNTQQ
TQHSGAFGNQSSSLGVTSLNQEPFDMNVRGSSNFLDHGRCSENWQSAVQLSSFPSNALSTSEA
FSHEQLPNNNLQESISWTSSHLSSSPLDLSSSMANPANLEDSRGNISQVSLNNNVIQNIDYTTKQ
QWGDSRHDYNGNMNNSFSRGDSLVPASGPMMDQSNLISDKMNDVSLFSQFSGDSTYVVPHL
DGEKSAFDTKPRSNEDFLFETKPQNGFNQNSFEPLENIMMSMIKSDQNNETPLMDGEFGFDAY
SLGSCI
>GaARR12
MRVERVISDENDSFPVGMRVLAVDDDPTCLLLLETLLTKCKYNVTTTSQAITALKMLRENKN
KFDLVISDVHMPDMDGFKLLELVGLEMDLPVIMLSANGDTKLVMKGITHGACDYLLKPVRIE
ELQNIWQHVIRRKKFDRKDRNGSGSQDKPYIDSSEAAGLGNVDQNGKFNKKRKDQNEDEDEE
RDENGHDNEDLSTQKKPRVVWSVELHRKFVAAVNQLGIEKAVPKKILELMDVENLTRENVAS
HLQKFRLYLKRISCVANQQANMAAALGSVDSTHLRMGTMNGLGNFHTLAGPDQIHNAAFRS
FQHSGVLRRLNTPAGVGISGLPSSGLVQLSPVQNLGNTSTNQSKLQSFVIPGNHNANILQGMP
MSLEFDQLQNNRAASHVGQFPSVDNTTVLPVSSISGSLIDAGCSSSPLLGVTSNSLLLEGSSQLP
TLHARNLRDSVSGFPNRSTLSDFTSIAPASNQLHGSKADIQDQVSPISCNAGKIISSAPQDWNAP
KKDAPYQSNVPSCSINSLRAVNGDMAWLGQCLDQNNPIFNRNVGFDFVDPLSIKCSEGENLA
MEPSVIEKEGYLMVQPSRQGSYIPDNEDENFAMEPSVIEKEGYLIVQPRRQGSYIPGNLGSLED
LASAITRQDHDKGKPTDGDFGYNDYSLRTCI
>GaARR13
MCHEQKEARHGVVRDGQGSGSIGENGSRIVERTLNVNNGSLEAIEVHDVSEIPQRQPRGSMIR
WERFLPFRTIKVLLVENEDLTRHLVSALLQNCSYEVVAVANGLQAWKLLEDPTNHIDIVLTEE
DMPVLSGSDLLCMIMNHKMLKNIPVIMMSSHDCINLVFKCLSKGAVDFLVKPIRKNELRNLW
QHVWRRCHSSSGSVSESGTLSKKSIKLKVNDEPENYAANSDEHDDDSDVPVGCNGSENGSGT
QSSWTKRAAEGESSQPMSSLNRFPGAPNSTCAQVVHVKHEKRRSPWTCVTQRKECQEQHEQL
LDATEGKDLEVRVESNNEWQCGNQCKNSPTHLAEAASKTFDRGWFEHQDENITGKDRIPDIIA
TLQQAECRASDAPGGPSDVPQLKDGACHGSEEKLSFELTLKRWQGASDGRNAANDGHNVLR
HSDSSAFSKYSTASSAKQALTGNVGSCSPLDHSSVTKKTEIMCTFPSHSNGILLNQSSVGSNNK
NDMTTTAKFVGPKPKALVDKSGSISTFKCLHSSSFQPMHGCCICSSQEVSPETVGHDTSLKIMA

ITDKQCRSSSSVNGSASGSNYGSNGHNGSETGLRAEHAVMEDGNGTASGRSGGSGANEDRVA
QRAAALTKFRQKRKERCFEKKVRYQSRKKLAEQRPRVKGQFVRRTISDWEGGKDNSSYDFTS
EDRYSDSLR
>GaARR14
MELMKSENIVQDRQPQPQEEEEIMQQEEEEEQRFHVLAVDDSVIDRKLLEKLLKASSYQVTCV
ESGEKALEYLGLLHHSSPASSSSQHHHQETQGHKVNLIMTDFSMPGTSGYDLLKRIKGSSWKD
VPVVVMSSENVPSRISMCLEGGAEEFMLKPLQLSDLHKIQAHLLKSLPHS
>GaARR15
MLYQIMDGDYGGSCGGCSEDMTVEFGYEPHVLAVDDNLIDRTLVEKLLKNSSCKVTTAENGL
RALEFLGLGSDQRNSLEGTVSKVNLVITDYCMPGMTGYELLKKIKESSVLKEVPVVIMSSENIP
TRINQCLEEGAQMFMLKPLKQSDVKQLKRHLMKYRS
>GaARR16
MELMKSSDNIIQDRTQQQHKQEPEMQAQEGQQEQGHHFHVLAVDDSVIDRKLLEKLLKVSSY
QVTCVDSGDKALEYLGLLNNLDSDSTASSSSSSSSSSSSNSSSCSQSSQREGLKVNLIMTDFCMP
GMSGYDLLKRVKGSSWKDVPVVVMSSENVPSRISMCLEGGAEEFMLKPLQLSDLEKIQAYLL
KSLHLSCTNIDKDDDDNNTDDNSDNNNDKVVNNDSNIDKGNNSTSMNNNFSKRKALSSEDTE
SRRPKIKGLAVAI
>GaARR17
MAPTLPSSDQASDQDNWDNGRLARKRQLLIEIVLRNRLTALVVDGDTTCRVLEQLLLRSYGV
QTQGVDNGRDAVALIASGVKFDLIIIDMILPVLNGLEATRQIRDMGVHCKMLGVTICSGESERQ
AFLAAGVDVFIEKPLDPEHLVPILRELDGQ
>GaARR18
MALTLPLGEASDQDKWDNGGLERKRQLLIEIVLRSRLTALMVDGDTTCWVLKQELLRSYGV
QTLGVDNGRDAIDLIASDAAFNLIIIEKILPVLNGLEVTRQICEIGMRCKMLAVTAYSGESERQA
FLATSVDVFIEKPLDLEHLVPILRELDRQ
>GaARR19
MDGGFDGDFPAGLKVLVVDDNRTCLLVLETMLRKLSYEVTTCQLARHALALLREDKNRFDIV
LCDLHMPELDGLKLLEIIGLEMDLPVVMMSSDDGKGVIMKGIIHGACDYLVKPVQMEAVRLI
WQHVVRKRQRALEDFQQLRGNIHATGRTLLLKQAKNAVDQMPARERRILKRERENEDEDDE
GELSEEVTTAKKPRVIWTQELHDIFVIAVNQLRQQAVPKKILERMQAMNVTGLSRANIASHLQ
KYRLHLRKGGGQPLADNRNVNLNPSIGQASSFNQFNLQFQQPTAIGSSCNQLPMQNLMITPQP
CTINDSVVPDSTQCFPTELSTNDLSQPNLLFQNDVPTSNVEHLYRNVGVNVSELSNPISSVDDSS
VDQLIYEPSFLVRQYDPEDFFHGGFPRSDSYVSVRDIEYLTSEEAPMKRISFFLR
>GaARR20
MRVERIINDTNDEFPIGIRVLAVDDDPTCLLLLETLLRRCKYNVTTTNQAITALKLLRQNKFDL
VISDVHMPDMDGFKLLELVGLEMDLPVIMLSANGDTNVVMKGITHGACDYLLKPVRIEELNN
IWQHVVRRKRFERKDRYNSDSQDKLRADSGEAVGMGSIGNNGKLKKKRKDQDEDEERDENG
HHNNEDPSFRKKSRVVWSAELHSKFVAAVNQLGMKNATPKKILELMNVQKLTTQNVGSHLQ
KFRLYLKRISCAANQEVNIAATVRMRSPNGLGNFHTLAGSNQLHNAAFRSFPPRGVLRRLNTH
AGLLIRDLPSPGTIRSGHGQSSVNSGNDQCKLQSFVSGIHNANILQGLPMSLELDQVQTNNGVS
HIGELQIADSTTVFPVSSSLIDATITGFSGNPLLGVTSNSLMLEASSQQAGNSRDIVPAIGFRNGN
TLSDLAPIAPASNRESNADLQREPIPINCNARELITSAPQEWNSPYQSNVTPCSMNSSIPVSGTM
VQFGLCLDQNNSMDSDSIGPSSFIDNSAMEQSIIDKEGYLMLEIWGQGSHIPYNIGLLEDLWEL
Q

>GaARR21
MTMEEKMGGSNREDEAMDRFPVGMRVLAVDDDPICLKVLGTLLLKCQYQVTTTNQAISALK
MLRENRNRYDLVISDVNMPDMDGFKLLELVGLEMDLPVIMLSAHSDTKLVMKGITHGACDY
LLKPVRIEELKNIWQHVVRRKKPDSKDRVNALNQDKASGGIGEAVQTSTSCSDQKFNKKRKD
QNEDEEDDGEDNEHENDDPSTQKKPRVVWSVELHRKFVAAVNHLGLDKAVPKKILDLMNVE
GLTRENVASHLQKYRLYLKRLSSVATQQANMVAALGGKDPSYLRMGSLDGFGDFRTLTGSG
RLSSASLSSYQPSGMFGRLNSSAALNLRGISSGVIQQGHPQTLSNSINGLGKIQPVGLPANQNQ
NGTLFQGIPTSIELNQLSQNKSANHFGELNPVNNPNVFGVATNFPDARMTVGSSSSSLSTPSGSP
LLLQANTQQAQHSGAIGNQSSLGVASLNQESFDMGVHGFSNFVDNGRCHENWQNTVQLSSFP
SNSLSTCEAFSHEQLHTNNLQESISWRSSHLSDNPIDLSASMANAGLEHSRGDMQCQISPSNNV
IHNIDYAAKRQWGENSSFAGADSLVSGAPYVQHLEAGKLAFNTKLRSNEDFLFEQRKPQNEFS
QNNFETLDIISSSMIKPEQYNETGMMEGELGYDAFPLGSCI
>GaARR22
MEEKMGGSNGEDSGEDQFPVGMRVLAVDDDPICLKILANLLCKCQYQVTTTNQAIIALKMLR
ENKNRYDLVISDVNMPDMDGFKLLELVGLEMDLPVIMLSAHSDTKLVMKGITHGACDYLLKP
VRIEELKNIWQHVVRRKKPDSKDELAAPNHDKARGGTGEAGQTWVACSSDQKVNKKRKDQS
EDEEEETEDNGRENEDSSCQKKPRVVWSVDLHRKFVAAVNQLGLGDQAVPKKILDLMNVEG
LTRENVASHLQKYRLYLRRLSSVATQQANMIVALGSRGPSHLRTGSLDGFGDFRSFTGPGRFS
SASLPSYQSRGTFGRLNSSAALTLSGISSGVIQPGNSVNGLGKIQPVVLPANQNQNGVLFQGITA
SIELNQLSQTKSTNHFGEYNCVDERNVFRISSSFPDARVVVGSSSNSLSTASGNALVLQGNTQQ
VQCSAAFENLPSFGMTSLNRESNDLSVRGSSNCLEHGRCSENWQGTVQLSNFPPSNEQLPSNN
LQESIPWRNSSPSNGCIALSSMASSAFLGDSRADMLCKAGLNNSYSHVDSRVSASGSMMDQSN
AVSSNTNDVSLFSHLNGEAPFAVWLSEGDKSSFDTDLRSNDNFLFLQSKPQNGFSQNNFESLE
DIMSPVFKLEQNNETAFMDGGFGFDVAYPLGSCM
>GaARR23
MAQSKHGGRKLGQSNKMTALVVDDNMINRTIHHRLLDNLGIENEVVSNGKEAVDIHYSGKM
FDLILMDMDMPIMNGIEATKKLREMGIRSVIAGVSSRAMEEEIQEFMEAGLDDYQEKPLTMPK
LVSIIHKING
>GaARR24
MSGNGASSSSLNMISGEDVVNNNLTALVVDDSPLLRLLHDIHLKKYGLKVQVAENGKVAVDL
FHLGASFDLVLMDKEMPVMNGVEATKELRAMGVTSMIVGVTSKDGPGEQQAFMEAGLDYC
FEKPLTPEIIGILLEELNKHNKKD
>GaARR25
MNVDGDGDKGLREQNHRLYDGSKRTTNGVVAEEHVMLEDVKVNKIAQNVKDGHVGAVQA
PAMRQIAQQQSQNAMCYWQRFLHLTTVTVLLVENDDSTRHVVTALLRNCCYDVVEAANVL
QAWKILEDLTNHIDLILAEVGMPSLSGLVLLSKIMSHKTRKNVPVIMMSSQDSMNLVFKCLSK
GAVDFLVKPIRKNELKNLWQHVWRRCHSSSGSGSESGTQXQKSVRSKSVEKSDNNSGSNDEE
NNGSIGLNIGDGSDDGSGTQNSWTKQAIEVDSPRLVSPPDQVAECPDSTCAQVVHSNAELSGK
KWVPVAAAKGCQEQDEQLDNVAVGKDLDIGMARNLDLQLECPVEVPMRTVGAKQINLLDM
SSSKFSEQIGKRQLDLNSESPSNKQKSEAANQTGITSKTTDLKKEIAENEDSNRLSKIPDGNDKT
INDSKEAPSVELGLKRIRGVKDAGTVVRDERNVLRRSDSSAFSRYNTASNANKVPXVNIGSSS
ALDSNLELTRKGSVCDNQSHLVNDLPNQSSNVGSNNIDMGSTTNNAFAKAAVDKNKSAASST
ADDVTTTAELAQARGIHHELQMLHPSNHYDQHHHLTHGMQKQQQRQPPEHDDLSLKKLAAD
APLCGSSNVLGGLVEGNVANYSVNGSASGSNHGSNGPNGSSNAVNTVGXKMVSDNGIAGKS

GSGDASGSGSGSGSGSGSGSGSGSKADQSKSAHREAALTKFRQKRKERCFQKKVRYQSRKRL
AEQRPRIRGQFVRQTVNNNDPASNVNSCDE
>GaARR26
MEVAGEVLASESQLHVLAVDDSHVDRKVIEKLLKISSCKVTTVESGARALQFLGLDEEKGSLG
FNGLKVNLIMTDYSMPGMTGYELLKRIKRSSTFREIPVVIMSSENILTRIDSCLEEGAEEFLVKP
VKLSDVKRVMNDCIRRGETEGNKRRRVDKRKFEDDNHASSSSSSSSSSIPSSPSSPLPSSPSTAA
TSSPLHSLKRAKLRDHN
>GaARR27
MNSSSGKSYMSTASSSVIWKAGGDVVADQFPAGLRVLVVDDDPTCLMILEKMLKACQYNV
TKCNRAETALSMLRENRNGFDIVISDVHMPDMDGFKLLEHIGLEMDLPVIMMSADDGKNVV
MKGVTHGACDYLIKPVRIEALKNIWQHVVRKRKNEWKDFEQSGSVEEGDRQPKQSEDADYS
SSANEGNWKSSKRRKDDEDENDERDDTSTLKKPRVVWSVELHQQFVAAVNQLGIEKAVPKKI
LELMNVPGLTRENVASHLQKYRLYLRRLSGVSQHPSNLNNSFMNPQDATFGPLDLQALAATG
QLPAQSLATLQAAGLGRSTAKSGIPMPLVDQRNIFSFENPKLRFGEAQQHVNNNKQMNLLHGI
PTTMEPKQLSGLHHTAQSMGNMNMPVLSHGSQSSQNNPLLMQMAQPQARGQMLNDSTVGL
APRLLSPMGQPMLSNGMATNVSTRNGVPENIRAPSYNPVSQTSSMLNFPMNHTSELPGNSFPL
VSTPGISSLAPKVAFQEDVNSEIKGSVGFMPSYDIFNDLNQHKPQNWELQNVGMMFNTSQHSN
SLQGNLDHTQSVLVQQGFSTGQNRNSTVISKAMFSAGDSTGHVNAQNGNQHLNSLFVDNTVR
VKSERVADAAPANLYPDHFGQEDLMSALLKQQDVIAPAENNDFDFDGYSIDNIPV
>GaARR28
MNVSSVKGSMSISSSTSTWKAGDTISDQFPAGLRVLVVDDDPTCLMILEKMLIACLYKVTKCN
RAETALSKLRENKNGYDIVLSDVHMPDMDGFKLLEHIGLEMDLPVIMMSADDGKQVVMKGV
THGACDYLIKPVRIEALKNIWQHVVRKRKNEWKEFEQSGSVEEGDRQPKQSDDADYSSSANE
GNWKGSKKRKDEEEETDERDDTSTLKKPRVVWSVELHQQFVAAVNQLGIDKAVPKKILELM
NVPGLTRENVASHLQKYRLYLRRLSGVSQHQSNLSTIISAQDPTFGSLSSLSGLDLQTLAATGQ
LPAQSLARLQAAGLGRATAKSGIPITLVDQRNIFSFENPKLRFGEGQQQHMTNKQQVNLLHGIP
TTMEPKQLVSLRHAAQSIGNMNMQVPPHGAQNSQNNPLLMQMGQQQQQSRGQILVDSTINH
APRLSSSMGQPILSNGIATNVSSRNGIPENIRAPGYSQTPSMLNFPVNHASELPGNSFALGSTPG
VSILTSKGAFQEDVNSEIKGSGGLMPSYDVFNDLNQHKPQSWELQNVGMAFDTSQHSNSLQG
NLDLTQSALGQQGFSSGQMNGHNRSAAVANKAMFSTGDVKELGSAQNVNQHLNNLLVDNTI
RIKSERVCDTSPANIFPDHFGQDDLMSALLKQQESVASSENEFDFDGYSMNNIPV
>GaARR29
MLSSGGGSGSCSKDMAVELGDEPHVLVVDDSLIDRKLVERLLKNSACKVTTAENALRAVEYL
RLGNDTLEGTVSEVNMIITDYCMPGMTGYELLKKIKESSILKEVPVIIMSSENIPTRISQCLEEGA
KMFMLKPLKRSDVKQLKWHLMKCRN
>GaARR30
MHSSGGGSGSCSKDMAMELGDEPHVLVMDDSLIDRKLVERLLKNSACKMTTAENALRAVNY
LRLGNDTLEGTVSEVNMIITDYCMPGMTGYELLKKIKESSVLKEVPVVIMSSENIPTRISQCLEE
GAKMFMLKPLKRSDVKQLKWHLMKCRN
>GaARR31
MLSCGGGSGGCSKDMAVELGDEPHVLAVDDNLIDRKLVERLLKNSSCKVTTAENALRALDY
LGLGNDRLEGTVSEVNMIITDYCMPGMTGYELLKKIKESSVLKEVPVVIMSSENIPTRISQCLEE
GAKMFMLKPLKQSDVKQLKWHLMKCRN
>GaARR32

MGMAIDRDDTQFHVLAVDDSLIDRKLIERLLKTSSFQVTAVDSGSKALEFLGLNNNDADDDEE
YEQRDSNTESVSSDEDQDHQDLGLGVNLIITDYCMPGMTGYDLLKKIKKSSSFKDIPVVIMSSE
NIPSRINRCLEDGAEEFFLKPVQLSDVNKLRPHLMKGRSKDIDSSHKTTSTRLITSGN
>GaARR33
MGEVLMSSDEVKTESEVDRENGEFEGNKRRKIMSGGSFVSGEAVNWESFLPTMALRVLLVEA
DDSTRQIVTALLRKCNYRVSAVPDGLQAWEMLKAKPHNIDLILTEVDLPSISGFALLTLIMEHE
ICKSIPVIMMSSQDSISTVYKCMLRGAADYLVKPLRRNELKNLWQHVWRRQQLIASGNTPQDE
SVGQKKVEAISENNAASNCSSGCLVGGERNKEQTEKGSEAQSTCTKPDMEAEDANMENMQE
FLLLPSNSQEHEVQTNFNQRLLVHEKKTGAVGACKDTEILVEAGDALGDSPRKAIDFMGTFDR
NCNSSLMNSTSKVGSLTHLDFSLGRCYPNAFENHATREKPTLWHPNSSAFTRYSSRPSQPLQST
LTSASDQKKESGTDSEKMLPNSISEYNSDTASPTLTPQRNTNPLTTGTTGQLKQTEVVASCTQQ
KLDQKMNSTEDRGHISPTTDQSATSSFCNGSLSQLNGIAYGSSAASNNSNIEQVAVVRASADS
KNDDVFPSPTGNSHRSIQREAALTKFRLKRKDRCFEKKVRYESRKKLAEQRPRVKGQFVRQPQ
ADPSHYGNSSDG
>GaARR34
MVEVGMEVDVEVGTAEETKKEEEEEEEEENDESSEVVRWEKFLPQMVLRVLLVEADDSTRQI
IAALLRKCSYRVVAAVPDGLMAWETLKDRPHNIDLILTEVELPSISGFALLTLVMEHDICKNIP
VIMMSSEDSFSMVLKCMLKGAADFLIKPVRRNELRNLWQHVWRRHMLAGGCANHSLPAKE
HNVEDTAENNAAENNQSSDYGSSTQKIKDESDTQGLSQWKCMSSNVSDTGREQQGNSVKPG
QESLQNEGQAEGISDKLFVSENSERFVEVAHCSGTCESIALKLEENSAFADEMAHNVVGLQSD
KRIAYVMIGVGCNDELRESSTGAIDLIDSFDKQPMDTFAISSLSDGANKLEFSPQLELSLRRPCS
RSAKSQGTNEKHILNHSDASPFSWYNNRKSLQPIFLTLDGNHEEGNSNPRQDLESNNGMRMD
DVHAGCNDVSPHFYHAKSSLPPACSPKQVGKQEYSPLPVSTSVHSDPDVDDSEQRYRWCDEA
TNSSNDRTLQGQNKQEPIEELRCSSLIDDQSACSHLCNDVENHEKSCTHGAVPSWSNASSVVT
LAAAIDKGTTMETFNNSNCFIHDGLKGMDTHRSSQREAALMKFRLKRKDRCFEKKVRYQNR
KRLAEQRPRVKGQFIRRMQHGTTLDNGDSHKI
>GaARR35
MAGEFPAGLKILIVDDDRTCLLVLERMLRKFSYQVTKCQQAREALALLRQDKNRFDIVLCDL
HMPDINGFELLQIIEVEMDLPVVMMSSDDGKGVVMKGIVHGACDYLVKPVRMEAIGLIWQH
VVRKKKKRFSGEITRSLPLQPADNAVPPMDKRSLKYRKRTSEDEDVAEDGESSEGKKPRMVW
TQELHDLFVAAVNELGRGNAVPKKILERMQAMNVTFLTRANIASHLQKYRMHLQKEGPVPSS
DSRDVNAYIDHRNLQFQPSPTTPYQLPMQNLITERANGNVLSIAPSHVDGGSNIFNSNIASESSC
SLPTQVTLYNLYRANLLYQNDFPPTSNGVAISNDNAFPPNDGVANSNDESFYCNVVDGTELSN
PFSAVAVDGTELSNPLSAVEELIHEPSFLVGQYDQQAFFRGPI
>GaARR36
MEEKRRSNCGEGFSVLVVDHDTTSLMLLASMLQLFSYQVTTTEVESVALSLIEEAKDRFKLV
MADVNMDDVNSLSFLRKLLKMNIPFILMSNEKGHKAAEKAVTYGASLHLEKPISKHDLKYLW
QYAYGRPAKRAKHLHDNELRTYDPNKNVGERDEQKKMLNIMPPTERKPRLLWNEELHQKFI
AAITALGYANARPKSILTMMDEPNLTQRQVASHLQKHKEHMKRLMSKASPSELASNANASTS
IYGKPGFLYMPQGYYSVLSQLGGHTHKYLNDSVAGTSQFSYLAQHSTAGRFSSDYHNEETLQ
FPNILESMHEKQNIIPDPSPSGNLVYENHNWEPWTEAARRMAEAEIGVGNAAGGVTWNQQSP
ELAQLLKHLEEEGDACGDCANQPNPDAVDQFCEWLKEAIQGNDNNP
>GaARR37
MAISYESNMNNVYAGVTVLLVDGDSTCLIILSKMLRSFGYKVVTTKQATDALCIIRDQQHKID

LVLMEACLHDMDKYELLETIRNISSIPIIVMSTDYDRNSVLGSLFKGATLHLEKPITMDDIKNL
WQFTLIKGQEINVPIVEAKSCTKEVPSMESALGVVVDGRRNLRDEKRKRPLEVENDKEGDNC
DQGSSTLKKPKLIWTNELHNRFLQAIDMLGSEAYPKKILQVMNVPGLRKENVSSHLQKHRLLL
KRQQEAIQNTISSTVSQAASHHALSEFSPRNGFHRFTDTAQTTSVAEQHGYINDLVQDNLNG
>GaARR38
MLSILAITTATQLGADVEHCENGNEALELVCDGLKAQRNYDYILMDCQMLPMNGYEATRRIR
IEEERYGVRIPIIALTAHTSGTEAMEAGMDAHLNKPLKTNELMEVIESIETKE
>GaARR39
MICTTNDLSEWKDFPKGLKVLLLDEDSNSAAELKSKLEAMDYIVYTFCNENEALSAVSSRPES
FHVAIVEINTNKKNGSFKFLETAKNLPTILTSNIHCISTMMKCIALGAVEFLKKPLSEEKLRNIW
QHVVRKAFNSVGTDLSESVKPVKESLVSMLYLQLENGEPKNKDLDKTQDASVIHENDPEPST
GSDKYPAPSTPQLEQGGRLSADGDCQDQANCSIEKESSEQDGESKSVETTSDNTIAEVTIPVGQ
PLGPRDTMVTEEADLVDGTKGESTTYSQTENGVNSKNSQAVAEKPSTVSGIHSSCLNKANRK
KSKVDWTPALHKKFVQAVDQLGIDQAIPSRILELMKVEGLTRHNVASHLQKYRMHRKHILPK
EDNRRWPQRDQTQRSCYPHKPIIAFPPHYSNHDVPVGPLYPMWGAPSSIQMWGSQGYPLWQP
TESWHWKPYPGAHADAWGCPVMPPPHGYSSAFTQVSSYQNASVFHCSGTMDNRSGMPQNS
VEHQPAEEVIDKVVKEAISKPWLPLPLGLKPPSTDTVLADLSKQGISTFPPHINGPNSSCHGTT
>GaARR40
MGDFVVMSCEEVKEVKGNSDTEMESGEFEGIKKKKRKQQQESCVYGEVVKWERWMALRVL
LIEADDSTRQIIAALLKKCNYKVSAVPDGLKAWEMLKGKPHNYDLILAEVDLPSISGFALLTLI
MEHELCKSIPVIMMSSQDSVSTVYKCMLRGAADYLVKPVRRNELRNLWQHAWRRQSSIIDGN
FPGDESIGRKQVEATSENDAARQTDKGSDTQSSCIKPDMETIQEFSHLIKGKSQPSGSQMHEAH
DGLNQNLLMHETKTSVNDCKDPNLTTAYKGVELECQRTNTNISAEAGNALADSPREAIDFMG
TFNRNFNFSSINSTCKFDSSPPLDLSLRRCNNNDFKDNVTRERPILWHPNSSAFTRYSNRLSLPQ
HSTLTSISNKKKESGSNSETILSNIVSEHDAATPSPMVTSQRNMIPSTAGATDQSRHTEVATSCM
EQRECPSSVNQHEPTFGVNPFHHSSLEMNSSRQFYDRLASGTNQMDQKLDSVEDKGHISPTVN
QSGTSSFCNGSISQLNGIAYGSSSASYSNVDQVIVRASTERKNDDNIPAPAGNSHRSIQREAALT
KFRLKRKDRCYEKKVRYESRKKLAEQRPRVKGQFVRQVQVDHPTQTIHPMASYNIEKCMD
>GaARR41
MVSTRSVNSPAMKITILVVDDDSTSLAIVSAMLREFRYEVTSVKTPAAALSVLRSNPSIDLVVT
DLHMPGMNGIELQKRINKEFKLPVIIMSSDDDENVMLESLAGGAVFFIVKPVDPIGLKNVWQY
AVAAKKGKSLLIEDMDRESSSSSPADGKLSLGGNTKSVSSVNNEKNDPKNGSKRKGASGKGR
DKDDNDDESKSPPPKKTKKPKIVWTNTLHNQFLEALRQIGLERAVPKKILERMNSTGLTRENV
ASHLQKYRIFLKRIAERGCFASKAVIDRFLKSNFAAGHPLLLKTAQEYSRLEHMERLRVLASYP
GLHESLMGHSSTRNVPLFYGHPGASSSNAAQQPLGYGQSRLLSNQTNQPLFPGSGNMLNNPN
LNNHLGYGNGNGIGIGSSSSVNGGGGFSSGLLNGGNSSLTYPNQVQARPDFYNAGPSSSSPFRF
GSAGFHSSGSTLGNGLFGSSSSSYPSLNSSSSGSIVVNGLFGSSSRSHPSLNSSSSGATLGNGLFG
SSSSSYPSLNNSSYPTLNPGYTNNAANSNRGMRFYEHLLNGSAPPITVDYGSMNQTRPTGDYG
SMNRTHNENINVPTMRTTPLDSLDFMREFPIPPVDNSTLIQGLGTGSTRLTEINSDQLLNNVPNL
DNEPRGGDGLLQDLVLESKKLANEVIHFYCSN
>GaARR42
MVMAASETKFHVLAVDDTLIDRKLIEKLLKTSSYQVTAVDSGTKALEFLGLNNNDHHDVEVN
LIITDYCMPGMTGYDLLRKIKESSSFKDIPVVIMSSDNIPSRINRCLEDGAEEFFLKPVQLSDVNK
LRPHLMKRRTQTNVNKRKAMDEIVSPDRTRARYNELEVK

>GaARR43
MTVEQIVSEGKDQFPIGMRVLAVDDDPTCLLLLGTLLRRCQYHVTTTSQAKTALKMLRENKN
KFDLVISDVHMPDMDGFKLLEHVGLEMDLPVIMLSANGDTKLVMKGITHGACDYLLKPVRIE
ELQNIWQHVVRRKKKDRCNSGSKDKPHPDSGEAAGIGNVDNNGKLNKKRKDQNEDEDDER
DENGHDNEDPSAQKKPRVVWSVELHRKFVAAVNQLGIDKAVPKKILELMNVEKLTRENVAS
HLQKFRLYLKRISCVANQQANMAAALGTADSAYLRMGSLNGLGNFHTLAGSDQLHNAAFRS
FPPSGVLGRLNTPAGLGIRGLPSPGTIQLGHVQNSGNPTNDLSKLQSFVPGNHNTNILQGMPMS
LELDRLQHNKNVGHIGELPTTDSMTVFPGSGNLVDARITGFSNNQLLGVTSNSLMLEGSSQQA
TSHTSVSAIGFQNGNALSDFTSIAPASNQLQDSKAGSQGQASPINCNAGQIIRSAPQEWNAPRK
DAPYQSHASINSSIPINGAMIQLGQCLDRNNSIFHRTTDLDSVGPLNFVDPLSIKHSEGDNSIMEP
SVIEKEGYLMFQPRPHGSHVPDNIGSLEDLASAMMKQEGGCNGYSLRTSI

G.Raimondii

>GrARR1
MKNSSGGKGSMSTASSITTWKAGDVVPVPDQFPAGLRVLVVDDDPTCLIILEKMLRNCSYDV
TKCNRAETALLKLRENRNGFDIVISDVHMPDMDGFKLLEHIGLEMDLPVIMMSADDGKDVV
MKGVTHGACDYLIKPVRIEALKNIWQHVVRKRKNEWKDLEQSGSVEEGDRQPKQSEDADYS
SSVNEGNWKSSKKRKDDEDETEDRDDTSTLKKPRVVWSVELHQQFVAAVNQLGIDKAVPKK
ILELMNVPGLTRENVASHLQKYRLYLRRLSGVSPHSCNPNNSFMNPQDETFGPLSSVHGLDLQ
TLTATGQLPAQCLATLQAAVLGRSTAKSSIPMPLVNQRNIFSFENPKLRFGEGQQQHVNNNNK
QVNLLHGIPTTMESKQLTSLRRTSQSIGNVSMQVAPHGAQSSQNNSSLIEMGQPLSRVQILNDS
TVPLSVGQPIVPNGIAANVSTRNGIPENIRAPGYNLVSQTSSILNFPMNHASELPVDSFSLRSTPG
MSDHTSKGAFHEDFNSEIKGSGGFLPTYDVFNDYQYKSQNWELQNAGMILEDSQHSNSLQGN
LDLAQSVLVQQGFPSGQINGQNRSVPIVSKAMFSAGDSTEPGNLLNVNHHLNTIRAENTVRVK
SGSVADGNPSNLFADHFGQEDLMIALLKQQQGIAPAENEFDFDGYSLDNIPV

>GrARR2
MNPSNGNGSMSTASSSGAWDGRDQFPAGLRVLVVDDDPTCLMIFEKMLKACLYQVTKCNKA
ETALSVLREHKNEFDIVISDVHMPDMDGFELLKHITSEMDLPVILISADARKQVVMEGVTYGA
CDYLIKPVRIETLKNIWQHVVRKKARNTV

>GrARR3
MVCTANDLSTWKDFPKGLKILLLDGDTNSANELKSKLEAMDYIVYAFHDENEAISAVSSRPES
FHVAIVEVSTDNNNGSFKFLETAKDLPTIMTSNIHCISTMMKCIARGAVEFLKKPLSEDKLRNI
WQHVVHKAFNTGGNDLTESLKPVKESVVSMLQQQPENLKPKNEDSETTEDASMIHENDSEPP
AGNDKYPAPSTPQLEQGGRLLDDRDCQDHTHCSIEKESGDQDGETESVETTGGNTTATGGQP
RGPSETNVKEEDDSVDGTMGESPQNRVDSKGSDVVAEKPSSCPDKANRKKSKVDWTPELHK
KFVQAVDQLGIDQAIPSRILELMEVEGLTRHNVASHLQKYRMQRRHILPKDDDRRWLQRPQT
QRICNYPHKPIMAFPPYNHVPVGPVYPMWRAPSHPPTIQMWGTQGYPPWQPTESWHWKPYP
GVQADAWGCPVMPPPQGYFSTFPQNASGFQPCNVDKRNGMPQTLAEHHPEEEVIDKMVKEAI
NKPGLPLPLGLKPPSTDSVLAELYRQGISTVPPHINASDR

>GrARR4
MESKELNLNKELKAGGAGNGFIDRSKVRILLCDNDTKSCEEVFSLLLKCSYQVTTVRSARQVI
DALNAEGPDIDIILTEVDLPMTKGMKLLKYIMRNNELRRIPVIMMSAQDEVSIVVKCLRLGAA
DYLVKPLRTNELLNLWTHMWRRRRELGLSEKNILNCDFDLVASDPSDANTNSTTLFSDDTDE
RSRKSSNPEMGVSTHQEDESAAATVEPPQSDSPECRPDVPGISDRRTGQFSSVPKKSELKIGESS
AFFTYVKSSAVKTSSTQVATPNHESAAQNKISEENLPQPGEQVVSDTRVHENGETWENNSQG

DDFRSSSSVPDSLSLERSSTPASMEFSQQRDFKEDKFSPALVPPRNETQHDVSGLPTQSAYLHY
MPGVLNQVMMPSSTQLFQNNLHDIHNHTSSPVVPQYNHLQQCLPHPHVSGMASFPYYPVNM
CMQPGQMPTGHSWPSFGNSSSNEVQPSKVDRREAALIKFRQKRKERCFDKKIRYVNRKRLAE
RRPRVRGQFVRKNGATVDLNGQPASADYDEDEEEEQASRDSSPEDDTSGC
>GrARR5
MVLAESGFSSPRNDAFPAGLRVLVVDDDPTWLKILEKMLKKCSYEVTTCCLAREALNLLRER
KDGYDIVISDVNMPDMDGFKLLEHVGLEMDLPVIMMSVDGETSRVMKGVQHGACDYLLKPI
RMKELRNIWQHVFRKKIHEVRDIESLEGFESIQMTRSGYDLFDDGHFLSGDDTTSGRKRKDAD
NKHDDRELSDPSSTKKARVVWTVDLHQKFVKAVNQIGFDKVGPKKILDLMNVPWLTRENVA
SHLQKYRLYLSRLQKESDIKNSFIGMKHSDLPSKDSTASFGPHKAMNMIPDDVPNGTYSFSAS
NSLAQNVDLKGQGDLKGITSAPVAEPKGALSIDIHDSHEAKSTQMSFDHSLGSVDSAVSFASF
NSTTPLQYPWTEIPEIQFKQECEPLHLENGFSQLPLPGPSMIENEANRSRIEVKPLLDECRSNFVE
HLGPVGAEDLFPIQSKSQSLNNQVFDLISATKSSMKTQDVGLNYLADSEFALRNLNASGVGVP
LATLSEDLQICWLQGDCYPMNYGLQDLECSTYDNPGLMAETPFHLYDVLRFDHEHLFDPTEY
YAIDQGLFA
>GrARR6
MATMHRVVQSSVSTSDATTTSYDGLTSCKAADIVISDQFPAGLRVLVVDDDITCLKILEQMLH
RCRYHVTTCPQAKVALNLLRERKGCFDVILSDVYMPDMDGYKLLEHVGLEMDLPVIMMSAD
GSTRAVMKGIRHGACDYLIKPIREEELKNIWQHVVRKKWNENKELEHSGSLDDTDQHKQRHD
DAEYASSVNDATETSLKPLKKRSNSKEEDDGEIDNDDPSTSKKPRVVWSVELHQQFVSAVNQ
LGIDKAVPKRILELMNVPGLTRENVASHLQKFRLYLKRISGVAQQGGIANPLCGPVEANVKIGS
LGSFNIQALAASGQIPPQTLAALHAELLGRSAGNLVVATDQPALLQATPQGPKCIQVDHGVAF
VQHSVKSESSSSKHFSQSFAPVEDVASGFRSWPSNNIGTAGPSNSGGLSSQNGNMLIDLLQQQQ
QLQKPQQRSTVSELRRSINVQPSCHVVPSQSSASFRAGNSPVSVTQNGSYSRTAVIDYSLLSSQS
NCPSLNIGQVSDVNLQTTGVLSGYIPPASVSPSVSSCSVNADNCASQQVQTSSMTFKASRRLPG
FVHSTSNIPDPYGSTKSGDLLNQEPFNNLGYINKGTCLPAKFAVDEFQSHLSSSSHGKVFSENIG
TRVKQEPSMEFGDNAKVGIPMLQQFPPNDLMSVFTE
>GrARR7
MIPCYFLFTVTTVKSSRQVIDALNAEGPDIDIILTEFDLQMTKGMKLLKYIMRNNELRRIPVIM
MSAQDEVSIVVKCLRLGAADYLVKPLRTNELLNLWTHMWRRRREQG
>GrARR8
MGEVVVTSEEVEEVKIDSETEEQENGEVEGNMRRRRRKKKKKKKKPGCSASGAVVNWER
FLPMPALRVLLVEADDSTRQIISALLRKCSYRVAAVPDGLKAWEMLKGRPHNVDLILTEVDLP
SISGFALLTLIMEHDICKSIPVIMMSSQDSVSTVYKCMLRGAADYLVKPIRRNELRNLWQHSIV
GGNSPQDESIGQKKVETTSENNAASNLSIGCLDGVRKNKEQTEKGSDAQSSCTKPEMEAESAQ
KENMQEFSHLIKVNSPPIESQKHEAHGGFNQNLLMHEMETEVVDSCKDAYTTLYKGVELENQ
RRDTRVLVEAGDALVDSPREAIDFMGTFNKNCTSSSINSAKKFDSSLFLDLSLRRSNPNVFENH
VTQERPTLWHPSSSAFTRYTSRVSQPLHSTLMSFSDQKKDSGTNSEKMLTNVMSENNSDTPSP
TLTSQRNTNPLTIGATVELKQTEVATPCTQHRLFPVPVPVKGIRLNNPCNGYNTIIPPMFCARSS
SSMAPSPSTANQQEPAFHLNLFRHSSFEANSSGQLYDRLASNTNQSTSQLLHKLDQKLDSIEDR
GHISPTTDQSASSSFCNGSLSQLNGVAYGSTGASNGNVDQAAVTRASTESKNDDSFPSPSGKPC
RSIQREAALMKFRLKRKDRCFEKKVRYESRKKLAEQRPRVKGQFVRQVQADPMHIETEHHYG
NSSDG
>GrARR9

MGNMATQTRFHVLAVDDSLIDRKLIERLLKTSSYQVTAVDSGSKALEFLGLNGENEDEERNSS
VESVSAADEDHQVGVNLIITDYCMPGMTGYDLLRKIKQSSSFKDIPVVIMSSENIPSRINRCLED
GAEEFFLKPVKLSDVNKLRPHLMKGITTKNEMQSNTNKRKGSEEIQSPDRTRPRYNELEVV
>GrARR10
MGMVQMNNNSPVTSGLVELNTHICDENTNIMDGVTGEGQGLSDEDESRINEDVENRNKGKK
AVVQRRGPLVCWERFLPLRSLKVLLVENDDSTRHVVCALLRNCGYEVTAVSNGLLAWKILED
LTNHIDLVLSEVVMPCLSGIGLLCKIMNHKTRKNIPVIMMSSHDSMNIVLKCLSKGAVDFLVKP
IRKNELKNLWQHVWRKCHSSSGSGSGSGIQTQKSTKSKSGDSDNNTGNNKEDDIGSVGLNAQ
DRSDNGSGTQSSWTKRAIEVDSSQPISARDQVMHSRSEVLGNIWVPVTITRECDGRDDELDNA
VKGKDLEIGVPKITALQLENPSEKVNTNVAGGNQEKLSELNPSKDDEKLEKAQLELTGEKLGV
DLVNQAADVIGVISKNTDAQIESAVFDIPDGLPQVSDTKGKVIYKTKEMPSLELSLKRLIDVGD
SGTSAPERNVLRHSDLSAFSRYNSGATANQAPVGNVGSCSPLDNSLEAANTDSMKNFHSNSN
NMPPNQQSNSNNNDMGSTTNNAFSKSAVLNDKPASKTSVPSSAFQPVQKGHATAMQPPAED
KADAAIGKKILAKAKGTDQQVRVQHHHHHYYHYHHHVHKMPQNQTLDNQDDLCQCGSSN
MSSAPHVEANAGNHSSNGSAPESNHGSNGQNGNITALNSRELNLESENGLLGKGGTVGGIGFG
SSNGADQNRFAQREAALNKFRQKRKERCFEKKVRYQSRKKLAEQRPRIRGQFVRQVPENKNK
DTNC
>GrARR11
MARNGGVAWMRTTEKIDGYDLSTSDTEEVHVLAVDDSLVDRKVIERLLRISSCKVTAVDSGR
RALQYLGLDEEIQKKETNGFDPFFYCDFQGLKVDLIITDYCMPGMTGYELLKKVKESSAFREIP
VVIMSSENVIARIDRCLEEGAEDFIVKPVKLSDVKRIIDYTTTELREGELREGARAKRRGINKRK
QREGDDDLSSSPPSTLSSSTSSSPSSSIQSATAPSSPSTLDSPTRRLKMTSSE
>GrARR12
MWKLELACLEYRIPSDNGVVQPKKVAFVGSVINKMEEIFLENLIDPQIDDDHTLKELTVFAFNT
QVLSLQYLCAVLHNCNYKVKSASSAVEVIEILRTNKHGIDIVLVDVDTADLNAFKVMETIGLE
TNLPVIMVTADSNLENITKGLAHGAVDCIIKPFEMEQIKNTIKSHVASNKTRGQNLNPLPGSNA
RMAVKDACCSSKLKKKRLAWSRELDAKFVKAVQILEKGSENVHPKRILDVMNEPGVTRAHIS
SHLQKYRLALKKRKADTNKQGLEVEPTNYCLKKREVGNFNADRRRLAVQSFNGVMSPSSSSP
DPKKQSQSLVYSCIDDHGPEFQTPDFYYNNHCLETNIQPRNVESEHVSGTTTLSPYFNGVDSKP
SILSSAAVYPFPNAVFLEPEANIAFHSHFDAISVPNPLYGFTIDCSTDVQYPSSTVFARNSETHAV
QNGTTISTSDTSSYHYVKPENEVSETYNSQIECSLFEEESFDWYSLLDDQYLSSLT
>GrARR13
MTLQEKRGGSNGEDGGKDRFPIGMHVLAVDDDPVCLKVLENLLRKCQYHVSTTNQATTALK
MLRENRNRYDLVITSVNMPDMDVFKLLELVGLEMDLPVIMLSTHGDTELVMKGITHGACDY
LLKPVRIEELKNIWQHVVRKNKPDFKYHVNALNQDNGHEDEDPSIQEKPRVVWSDEVHRKFV
SAVNELGLDSEFYAV
>GrARR14
MEEKLGGSNGEDGGRDRFPIGMRVLAVDDDPICLKVLENLLRKCQYHVTTTNQAITALKMLR
ENRNRYDLVISDVNMPDMDGFKLLELVGLEMDLPVIMLSAHSDTKLVMKGITHGACDYLLKP
VRIEELKNIWQHVVRKKKPDSKDQINASNQDKSRGGTGETGPTSSSSDQKVSKKRKDQSEDED
EEGDDNGHEDPSTQKKPRVVWSVELHRKFVSAVNQLGLEKAVPKKILDLMNVDGLTRENVA
SHLQKYRLYLKRLSSVATQQANMVAALGSKDPSYLRMGSLDGFGDFRTLTGPGRISSASLSSY
QPGGLFGRLNSSSALSLRGISSGVIQSGHSQTLNNPINGFGKIQPAVVPANQNQNGTLFQGIPTSI
NQLSQNKPTNHFGEFNRGNDPNAFGVATNFQDARVTVGGSSNTLPVSSGNPLLLQSNTQQTQ

HSGAFGNQPSSLGVTSLNQEPFDMNVRGSSNFLDHGRCSENWQSAVQLSSFPSNALSASETFS
HEQLPSNNLQESISWTSSHLSSSPLDLSSSMANPANLEDSRGNISQVGLNNNVIQNIDCTTKQQ
WGDSRHDYNGNMNNSFSRGDSLVPASGPMMDQSNVISDKMNDVSLFSQFSGDSTYVVPHLD
GEKSAFGTKSRSNDDFLFETKPQNGFNQNSFEPLENIMMSMIKSDQNNETPLMDGEFGFDAYS
LGSCI
>GrARR15
MRVERVISDENDSFPVGMRVLAVDDDPTCLLLLETLLTKCKYNVTTTSQAITALKMLRENKN
KFDLVISDVHMPDMDGFKLLELVGLEMDLPVIMLSANGDTKLVMEGITHGACDYLLKPVRIE
ELQNIWQHVIRRKKFDRKDRDGSGSQDKPYIDSSEAAGLGNVDHNGKFNKKRKDQNEDEDEE
RDENGHDNEDLSTQKKPRVVWSVELHRKFVAAVNQLGIEKAVPKKILELMNVENLTRENVAS
HLQKFRLYLKRISCVANQQANMAAALGSVDSTHLRMGTMNGLGNFHTLAGPDQIHNAAFRS
FQHSGVLRRLNTPAGVGISGLPSSGLVQLSPVQNLGNTSTYQRKLQSFVIPGNHNANILQGMP
MSLEFYQLQNNRAASHVGQFPSVDNTTVLPVSSISGSLIDAGCSNSPLLGVTSNSLLLEGSSQLP
TLHASNLRDSVSGFPNRSTLSDFTSIAPASNQLHGSKADIQDQAFPISCNAGKIISSAPQDWNAP
KKDAPYQSNVPSCSINSLRAVNGDMAQLGQCLDQNNPIFNRNVGFDFVDPLSIKCSEGENLAM
EPSVIEKEGYLMVQPSRQGIYIPDNEGENFAMEPSVIEKEGYLIVQPRRQGSYIPGNLGSLEDLA
SAMTKQDHDKGKPTDGDFGYNDYSLRTCI
>GrARR16
MCHEQKEARHGVERDGQGSGSIGENGSRIVERTLNVNNGSLEAIEVHDVSEIPQQQPRGSMIR
WERFLPFRTIKVLLVENEDLTRHLVSALLQNCSYEVVAVANGLQAWKLLEDPTNHIDIVLTEE
DMPVLSGSDLLCMIMNHKMLKNIPVIMMSSHDCINLVFKCLSEGAVDFLVKPIRKNELKNLW
QHVWRRCHCSSGSGSVSESGTLSKKSIKSKVNDEPENYAANTDEHDDDSDVLVGCNGSENGS
GTQSSWTKRAAEGESSQLMSSLNRFPDAPNSTCAQVVHVKHEKCGSPWTCVTQRKECQEQH
EQLFDATEGKDLEVRVESNHEWQCGNQCENSPTHLAEAASKLFDRGWFEHQDENITGNDRTP
DIITTLQQAECRASDAPGGPSDVPQLKDGACHGSEEKLSFELTLKRWQGASDGRNAANDGHN
VLRHSDSSAFSKYSTASSAKQALTGNVGSCSPLDHSSVTKKTEVMCTFPSHSNGILLNQSSVGS
NNKNDMTTTAKFVGPKPKALVDKSGSISTFKCLHSSSFQPMHGCCICSSQEVSPETVGHDTSLK
IMAITDKQCRSSSSVNGSASESKYGSNGHNGSETGLRAEHAVTEDGNGTASGRSGGSGADEDR
VAQRAAALTKFRQKRKERCFEKKVRYQSRKKLAEQRPRVKGQFVRRTVSDWGGKDSLSYDF
TSEDRYSDSLR
>GrARR17
MELMKSENIVQDRQPQPQEEEEIMQQEEEEEQRFHVLAVDDSVIDRKLLEKLLKASSYQVTCV
ESGEKALEYLGLLHHSSPASSSSSQHHHQGHKVNLIMTDFSMPGTSGYDLLKRIKGSSWKDVP
VVVMSSENVPSRISMCLEGGAEEFMLKPLQLSDLHKIQAHLLKSLPHS
>GrARR18
MDGDYGGSCGGCSEDMTVEFGYEPHVLAVDDNLIDRTLVEKLLKNSSCKVTTAENGLRALEF
LGLGSDERNSLEGTVSKVNLVITDYCMPGMTGYELLKKIKESSVLKEVPVVIMSSENIPTRINQ
CLEEGAQMFMLKPLKQSDVKQLKRHLMKYRS
>GrARR19
MELMKSSDNIIQDRTQQQHKQEPEMQAQEGQQEQGHHFHVLAVDDSVIDRKLLEKLLKVSSY
QVTCVDSGDKALEYLGLLNNLDSDSTASSSSSSSSSNSSSCSQSSQREGLKVNLIMTDFCMPG
MSGYDLLKRLKGSSWKDVPVVVMSSENVPSRISMCLEGGAEEFMLKPLQLSDLEKIEAYLLKS
LHLSCTNIDKDDDDNNADHNSDNNNDKVVNNDSNIDKGNNSTSMNNNFSKRKALSSEDTESR
RPKIKGLAVAI

>GrARR20
MEEKMGGSNREDEALDRFPVGMRVLAVDDDPICLKVLGTLLLKCQYQVTTTNQAISALKML
RENRNRYDLVISDVNMPDMDGFKLLELVGLEMDLPVIMLSAHSDTKLVMKGITHGACDYLL
KPVRIEELKNIWQHVVRRKKPDSKDRVNALNQDKASGGIGEAVQTSTSSSDQKFNKKRKDQN
EDEEDDGEDNEHENDDPSTQKKPRVVWSVELHRKFVAAVNHLGLDKAVPKKILDLMNVEGL
TRENVASHLQKYRLYLKRLSSVATQQANMVAALGGKDPSYLRMGSLDGFGDFRTLTGSGRL
SSATLSSYQPSGMFGRLNSSAALNLRGISSGVIQQGHPQTLSNSINGLGKIQPVGLPANQNQNG
TLFQGIPTSIELNQLLQNKSANHFGELNPVNNPNVFGVATNFPDARMTVGSSSSSLSTPSGSPLL
LQANTQQAQRSGAFGNQSSLDVASLNQESFDMGVHGFSNFVDNGRCHENWQNTVQLSSFPS
NSLSTCEAFSHEQLHTNNLQESISWRSSHLNDNPIDLSASMANAGLEDSRGDMQCQISPSNNVI
HNIDYAAKQQWGENSSFAGVDSLVAGAPYVQHLEAGKLAFNTKLRSNEDILFEQRKPQNEFS
QNNFETLDVISSPMIKPEQYNETGMMEGELGYDAFPLGSCI
>GrARR21
MRVERIINETNDEFPIGIRVLAVDDDPTCLLLLETLLRRCKYNVTTTNQAITALKLLRQNKFDL
VICDVHMPDMDGFKLLELVGLEMDLPVIMLSANGDTNVVMKGITHGACDYLLKPVRIEELNN
IWQHVVRRKRFERKDRYNSDSQDKLRADSGEAVGMGSIGNNGKLKKKRKDQDEDEERDENE
DPSIRKKPRIVWSAELHSKFVAAVNQLGMKNATPKKILELMNVEKLTTQNVGSHLQKFRLYL
KRISCAANQQVNIAAAVRMRSPNGLGNFHTLAGSNQLHNAAFRSFPPRGVLGRLNTHAGLLIR
SLPSPGTIRSGHGQSAVNSGNDQSKLQSFVSGIHNANILQGLPMSLELDQVQTNNGVSHIGELPI
ADSTTVFPVSSSLIDATITGFSGNPLLGVTSHSLMLEASSQQAGNSRDIVPAIGFRNGNTLSDFA
PLAPASNRESNADLQCEPIPINCNAGELITSAPQEWNSPYQSNVTPCSMNSSIPVSGTMVQFGLC
LDQNNSMDSDSIGPSSFIDNSAMEQSIIDKEGYLMLEPWEQGSHIPYNIGLLEDLWELQ
>GrARR22
MMDSRLQKKKSLALRHLLMWMRSLNLLELKKLGVEEGFIKFLGPYYMLLITKRRRIGALFRH
NVYAVSKRKRTVTTVKSARQVIDALNAEEPDIDIILTEVDLPMTKGMKLLKYIMWNNELRRIP
VIMMSAQDEVSIVVKCLRLGAADYLVKPLRTNELLNLWTHMWRRRREG
>GrARR23
MDGGFDGDFPAGLKVLVVDENRTCLLVLETMLRKLSYEVTTCQLARHALALLREDKNRFDIV
LCDLHMPEMDGLKLLEIIGLEMDLPVVMMSSDDGKGVIMKGIIHGACDYLVKPVQMEAVRLI
WQHVVRKRQRALGDFQQLRGNIHATGRTLLLKQAKNAVDQMPARERRILKRARENDDEDED
DEDDEGELSEEVTTAKKPRVIWTQELHDIFVIAVNQLRQQAVPKKILERMQAMNVTGLSRANI
ASHLQKYRLHLRKGGGQPLADNRDVNLNPSIGQASSFNQFNLQFQQPTATGSSCNQLPMQNL
MITPQPCTINDSVVPDSTQCFPTELSTNDLSQPNLLFQNDVPTSNVEHLYRNVGVNVSELSNPIS
SVDDSSVNQLIYEPSVLVRQYDQGDFFHGGFPRSDSYVSVHSNLIFFMIMKNTNK
>GrARR24
MADSSVFSLVSFNGLMFLHNRVIWKDGLTYDQLLECATECSPKLGQIVLVACSPPLILLFKGEE
DGGMHRRHASPSVRDRNVKMPISSQSSASAHLPGYQTSAIVAMDRTSTLSSGTPLASGLNLSQ
SKSLGKGAGRTLESVLHASKQKVPMEVCSEVWTYPKNRGVDPPSSRDPPFPAVVPASNSLTSS
LGLESTTSTVGKGSNHNGGLIMSDIISQIQASKDSGKLSYRTSATTESLPAFISYSAKRASERQER
GSLEEKIDIMEARRSVNPHVDRQYLDTPYRDVNSRNLQNNQVPNFQRPLLRKHIAGRMSAGR
RKSFDDCQLSLGEMSSYVEVPASLNDALSEGLSLSSDWSAREVVQNFEKVMKLFFQHLDDPH
HKVAQAALSTLVDIIPSCQEPFESYMERILTHVFLRLIDPKELDSGRLARKRQLLTKIAPRNRLIA
FVVNGISTYRVLEQGLLRSYGVKTQGVDNGKGAVDLIAFGAFGAKFDLTMTDMILLILNGLK
ATLHIKNKCFMFCVESTCVIIWFFLLLMGRQQGRFMKWGCVAFLSASVDVFIEKPLDPQHLVPI

LRELDGQ
>GrARR25
MSGNGASSSSLNMISGEDVVNNNLSALVVDDSPLLRLLHDIHLKKYGLKVQVAENGKVAVDL
FHLGASFDLVLMDKEMPVMNGVEATKELRAMGVTSMIVGVTSKDGPGEQQAFMEAGLDYC
FEKPLTPEIISFLLEELNKHNNKN
>GrARR26
MVCTRNDLSAWKDFPKGLRVLLLDEDTNSAAEIKSKLEAMDYIVYTFCNENEALSAVSSRPES
FHVAIVEVCTNSNNGGFKFLETAKDLPTIMTSNIHCISTMMKCIALGAVEFLRKPLSEDKLRNI
WQHVVHKAFSAGGDDLSESLKPVKDYVASMLHQQLENGECKHEDLDKIEDASMIHENDHEP
SAVNNKYPAPSTPQLQQGGRLIVNGDCQEHTNCSMEKESGEPDGESKSVETTSGHTIAEVIAPV
GQHQKPRETMVKEEADSVDGAKGERTLDSHPQDRVNSEDSRAGAEKPNTVSGLHSSRPNKA
NRKKLKVDWTTELHKKFVQAVDQLGIDQAIPSRILELMKVEGLTRHNVASHLQKYRMHKRHI
LPKEDDRRWPQRDQTQRSCHPHKPIMAFPPYHSNPVPVGPVYPMWGTPHPASIQTWGSQGYY
HPWQPTESWQWKPYPGVHADAWGCPVMPIPQGNCSTFTQNGYGFHCSRTMDNRSGMPQNSI
QHQPAEEVIDEVVKEAINKPWLPLPLGLKPPSTESVLAELSRQGISTIPPRNQRL
>GrARR27
MAQSKHGGRKLGQSNKMTALVVDDNMINRTIHHRLLDNLGVENEVVSNGKEAVDIHYSGK
MFDLILMDMDMPIMNGIEATKKLREMGIRSVIAGVSSRAMEEEIREFMEAGLDDYQEKPLTMS
KLVSIIHKING
>GrARR28
MEEKMGGSNGEDSGEDQFPVGMRVLAVDDDPICLKILANLLCKCQYQVTTTNQAIIALKMLR
GNKNRYDLVISDVNMPDMDGFKLLELVGLEMDLPVIMLSAHSDTKLVMKGITHGACDYLLK
PVRIEELKNIWQHVVRRKKPDSKDELAAPNHDKSRGGTGEAGQTWVACSSDQKVNKKRKDQ
SEDEEEETEDNGHENEDSSSQKKPRVVWSVDLHRKFVAAVNQLGLGDQAVPKKILDLMNVE
GLTRENVASHLQKYRLYLRRLSSVATQQANMVVALGSRGPSHLRTGSLDGFGDFRSFTGPRR
FSSASLPSYQSRGTFGRLNSSAALTLSGISSGVIQPGNSVNGLGKIQPVVLPANQNQNEALFQGI
TASIELNQLSQTKSTNHFGEYNCVNERNVFRISSSFPDARVVVGSSSNSLSTASGNALVLHGST
QEVQCSAAFENLPSFGMTSLNRESNDLSVRGSFNCLEHGRCSENWQGTVQLSNFPPSNEQLPS
NNLQESIPWRNSSPSNSRIALSSMASSAVLGDSRADMLCKAGLNNSYSHVDSRVSASGSMMD
QNNAVSSNTNDVSLFSHLNGEAPFAVRHSEGDKSSSDTDLRSNDNFLFLQSKPQNGFSQNNFE
SLEDIMSPVFKLEQNNETAFMDGGFGFDVAYPLGSCM
>GrARR29
MNVDGDGDKGLRELNHRLYDGSKRTTNGVVAEEHVMLEDVKVNKIAQNVKDGHVVAVQA
PAMRQITQQQPQNAMCYWQRFLHLTTVTVLLVENDDSTRHVVTALLRNCCYDVVEAANVLQ
AWKILEDLTNHIDLILAEVGMPSLSGLVLLSKIMSHKTRKNVPVIMMSSQDSMNLVFKCLSKG
AVDFLVKPIRKNELKNLWQHVWRRCHSSSGSGSESGTQTQKSVRSKSVEKSDNNSGSNDEEN
NGSIGLNIGDGSDDGSGTQNSWTKQAVEVDSPRLVSPSDQVAECPDSTCAQVVHSNAELSGN
KWVPVAAAKGCQEQDEQLDNVAVGKDLDIGMPRNLDLQLECPVEVPIRTVGAKQINLLDMS
SSKFSEQIEKRQLDLNSESPSNKQKSEAANQTGTTSKTTDLKKEIAENEVSNRLSKIPDVNDKTI
NDSKEPPSVELGLKRLRGVKDAVTVVRDERNVLRRSDSSAFSRYNTASNANKVPVVNIGSSSA
LDSNLELTRQGSVCNNESHLVNELPNQSSNVGSNNIDRGSTTNNAFAKAAVDKNKSAASSTV
RSLHPSSIFQPMKNDLLRATQKVVFDKADDVSTTAGLAQARGIHHELQMQHPSNHYDQHHHL
THGMQQQQPPEHDDLSLKKLAADAPHCGSSNVLGGLVEGNAANYSVNGSASGSNHGSNGPN
GSSNAVNTVGTNMESDNGIAGKSGSGDVSGSGSGSGSGGKADQSKSAHREAALTKFRQKRKE

RCFQKKVRYQSRKRLAEQRPRIRGQFVRQTVNNDPASNSCDE
>GrARR30
MEVAGEVLASESQLHVLAVDDSHVDRKVIEKLLKISSCKVTTVESGARALQFLGLDEEKGSLG
FNGLKVNLIMTDYSMPGMTGYELLKKIKRSSAFREIPVVIMSSENILTRIDSCLEEGAEEFLVKP
VKLSDVKRVMSDCIMRGETEGNNRRRVDKRKFEDDNHASSSSSSSSSIPSSPSSPLPSSPSTAAT
SSPLHSLKRPKLRDHN
>GrARR31
MNSSSGKSYMSTASSSVLWKAGGDVVADQFPAGLRVLLVDDDPTCLMILEKMLKACQYNVT
KCNRAETALSMLRENRNGFDIVISDVHMPDMDGFKLLEHIGLEMDLPVIMMSADDGKNVVM
KGVTHGACDYLIKPVRIEALKNIWQHVVRKRKNEWKDFEQSGSVEEGDRQPKQSEDADYSSS
ANEGNWKSSKRRKDDEDENDERDDTSTLKKPRVVWSVELHQQFVAAVNQLGIEKAVPKKIL
ELMNVPGLTRENVASHLQKYRLYLRRLSGVSQHPSNLNNSFMNPQDATFGPLDLQALAATGQ
LPAQSLATLQAAGLGRSTAKSGIPMPLIDQRNIFSFENPKLRFGEAQQQHVNNNKQTNLLHGIP
TTMEPKQLAGLHHTAQSMGNMNMPVLSHGSQSSQNNPLLMQMAQPQARGQMLNDSTVGLA
PRLLSPRGQSMLSNGMATNVSTRNGVPENIRAPSYNPVSQTSSMFNFPMNHTSELPGNSFPLVS
TPGISSLAPKVAFQEDVNSEIKGSVGFMPSYDIFNDLNQHKPQNWELQNVGMMFNSSRPSNSL
QGNLDHTQSILVQQGFLSGQNRNSTVISKAMFSAGDSTGHVNAQNSNQHLNSLLDNTVRVKF
ERVADAAPANLYPDHFGQEDLMSALLKQQDIIAPAENNDFDFDGYSIDNIPV
>GrARR32
MSVQFFYYVCKVPDQSGGSTSKAKGKEKQWASFLYIMYVFFSAVSITNQETTALKMLRENRS
RYDLVITSVNMPDMDAFKLLELVGLEMDLPGITHGTCDYLLNPVCIDELKNIWQHVVRKKIDF
KDHINALNQDNKQKDEDPSIQEKLCIGLNNVAPLGSKDSSYLRRGPLDGFGYLRILTRPGKISS
AFLPYYQPGGMFGILNSSIDLSPRGIYSSIIQPRHSQTSNNPINGSRKIQPAVNKPTKGCGEFNHV
NDLNFQDARVAVGGSCNTLPVSSSNPLLLQSNTQQTKHSGAFGNQFSLSTDLLDLYSFKAMSA
NLEDFKGDISNVGLNNYIIVNMDYATKQQWGDSRHDYNGNTNHSVSQANA
>GrARR33
MNVSSVKGSMSISSSTSTWKAGDTISDQFPAGLRVLVVDDDPTCLMILEKMLTACLYKVTKC
NRAETALSKLRENKNGYDIVLSDVHMPDMDGFKLLEHIGLEMDLPVIMMSADDGKQVVMKG
VTHGACDYLIKPVRIEALKNIWQHVVRKRKNEWKEFEQSGSVEEGDRQPKQSDDADYSSSAN
EGNWKGSKRRKDEEEETDERDDTSTLKKPRVVWSVELHQQFVAAVNQLGIDKAVPKKILEL
MNVPGLTRENVASHLQKYRLYLRRLSGVSQHQSNLSTIISAQDPTFGSLSSLSGLDLQTLAATG
QLPAQSLARLQAAGLGRATAKSGIPITLVDQRNIFSFENPKLRFGEGQQQHMTNKQQVNLLHG
IPTTMEAKQLVSLRHAAQSVGNMNMQVPPPGPQSSQNNPLLMQMGQQQQQQQSRGQILVDS
TINHAPRLSSMGQPILSNGMATNVSSRNGIPENIRAPGYSQTPSMLNFPMNHASELPGNCFPLG
STPGVSNLTSKGAFQEDVNSEIKGSGGFMPSYDVFNDLNQHKPQSWELQNVGIAFDSSQHSNS
LQGNLDLTQSALGQQGFSSGQMNGHNRSAAVASKAVFSTGDVKELRSAQNVNQHLNNLLVD
NTIRVKSERVCDTSPANIFPDHFGQDDLMSALLKQQESVASSENEFDFDGYSMNNIPV
>GrARR34
MLGSGGGSGSCSKDMAVELGDEPHVLVVDDSLIDRKLVERLLKNSSCKVTTAENALRAVEYL
RLGNDTLEGTVSEVNMIITDYCMPGMTGYELLKKIKESSVLKEVPVIIMSSENIPTRISQCLEEG
AKMFMLKPLKRSDVKQLKWHLMKCRN
>GrARR35
MLGSGGGSGSCSKDMAVELGDEPHVLVVDDSLIDRKLVERLLKNSSCKAENALRAVNYLRLG
NDTLEGTVSEVNMIITDYCMPGMTGYELLKKIKESSVLKEVPVVIMSSENIPTRINQCLEEGAK

MFMLKPLKRSDVKQLKWHLMKCRS
>GrARR36
MAHSVLSDWIYLPAQTPPSLLLNYFSFFPLKSNQIFLISFLTFSPSKQSLLYKMLSCGGGSGGCS
KDMAVELGDEPHVLAVDDNLIDRKLVERLLKNSSCKVTTAENALRALDYLGLGNDTLEGTVS
EVNMIITDYCMPGMTGYELLKKIKESSVLKEVPVVIMSSENIPTRISQCLEEGAKMFMLKPLKQ
SDVKQLTWHLMKCRN
>GrARR37
MGMAIDRDDTQFHVLAVDDSLIDRKLIERLLKTSSFQVTAVDSGSKALEFLGLNNNDADDDEE
DEQRDSNTESVSSDEDQDHQDLGLGVNLIITDYCMPGMTGYDLLKKIKKSSSFKDIPVVIMSSE
NIPSRINRCLEDGAEEFFLKPVQLSDVNKLRPHLMKGRSKDIDSSHKTTSTRLITSGN
>GrARR38
MGEVLMSSDEVKTESEIDRENGEFEGNKRRKKMSGGSFVSVEAVNWESFLPTMALRVLLVEA
DDSTRQIVTALLRKCNYRVSAVSDGLQAWEMLKAKPHNIDLILTEVDLPSISGFALLTLIMEHK
ICKSIPVIMMSSQHSISTVYKCMLRGAADYLVKPLRRNELRNLWQHVWRRQSLIASGNTPQDE
SVGQKKIEAISDNNAASNRSSGCLVGGERNKEQTEKGSEAQSTCTKPDVEAEDANMENMQEF
LLLPSNSQKHEVQMDFNQRLLVHEKETGVDGACKDTKISVEAGDAVGDSPRKAIDFMGTFDR
NCNSSSMNSTSKVGSSTHLDFSLGRCSPNAFENHATREKPTLWHPNSSAFTRYSSRPSQPLQST
LTSVSDQKKESGTDSEKMLPNSIDEYNSDTPSPKLTPQRNTNPLTTGSTGQLRQTEVAASCTQQ
VVLPVLVPSPSLANQKELACCVNPFHHPSFESNSSGQFYDRLASNTNQLTNQPLQKLDQKMNS
TEDRGHISPTTDQSATSSFCNGSLSQLNGIAYGSTAASNNSNIDQEAVVRASADSKNDDVFPSP
TGNSHRSIQREAALTKFRLKRKDRCFEKKVRYESRKKLAEQRPRVKGQFVRQPQADLSHYGN
SSDE
>GrARR39
MVEVGMEVDVEVGTAEETKKKKEEEEENDESSEVVQWEKFLPQMVLRVLLVEADDSTRQIIA
ALLRKCSYRVVAAVPDGLMAWETLKDRPHNIDLILTEVELPSISGFALLTLVMEHDICKNIPVI
MMSSEDSFSMVLKCMLKGAADFLIKPVRRNELRNLWQHVWRRHMLAGGCAHHNLPAKEHN
VEDTAENNAAENNQSSDYGSSTQKIKDESDTQGLSQLKCMSSNVSDTGREQQGNFVKPGQES
LQNEGQAEGISDKLFVSENSKRFVEVAHCSGTCESIASKLEENSAFVDEMAHNVVGLQSDKRI
AYVMIGVGCNDELRESSTGAIDLIDSFDKQPMGTFAISSLSDGANKLEFSPQLELSLRRPCSRSA
KSQGTNEKHILNHSDASPFSWYNNRKSLLPIFLTLDGNREEGNSNPRQDLESNNGMRMDDVH
AGCNDVSPHFYHAKSSLPPACGPKQVGKQEYSPLPVSTLVHSDPDVDDSEQCYGWCDEATNS
SNDQTLQGQNKQEPIEELRCSSLIDYQSACSRLCNDVENHEKSCTHGAVPSWSNASSVVTLAA
AIDKGTTMETFNNSNCFIHDGLKGMDTHRSSQREAALMKFRLKRKDRCFEKKVRYQNRKRL
AEQRPRVKGQFIRQVQHGTPLDNGDSHKI
>GrARR40
MTMAGEFPAGLKILIVDDDRTCLLVLERMLRKFSYQVTKCQLAREALALLRQDKNRFDIVLC
DLHMPDINGFELLQIIEVEMDLPVVMMSSDDGKGVVMKGIVHGACDYLVKPVRMEAIGLIWQ
HVVRKKKKRFSGEITRSLPLQRADNAVPAMDKRSLKYRKRTSEDEDVAEDGESSEGKKPRMV
WTQELHDLFVAAVNELGRGNAVPKKILERMQAMNVTFLTRANIASHLQKYRMHLQKEGAVP
SSDSRDVNAYIDHRNLQFQPSPTTPYQLPMQNLITERANENILSIAPSHVDGGSNIFNSNIASESS
CSLPTQVTLYDLYRANLLYQNDFPPISNGVAISNDNEFPPNDGVANSNDESLYCNVVDGTELS
NPFSAVAVDGTELSNPLSAVAVDGTELSNPLSAVEDLIHEPSFLVGQYDQQAFFRGPI
>GrARR41
MKNSSGGKVSMSTASLLTTWKVRDVVPVPDQFPTSLRVLVVDDDPTCLRFLEKMLRNCSYD

VTKCNRVKTALSKLRENRNGFDIITSDVYMPDMDGFKLLEHIGLDIDLPIIKVVMKGVRHGAC
DYLIKPVRIEALKNIW
>GrARR42
MASTTKTEGSIFLDNRRPFVAHILFQVYEGLDIGAREKQGNPKVDDGFKAPKEKITCIAASVDV
DEEFESSGVEETRMTKTLDEKKSEEEELKDKGNEERRKRTVTTVKSARQVIDALNAEGPDIDII
LTEVDLPMTKGMKLLKYIMRNNELRRIPDIMMSAQDEVSIVVKCLRLGAADYLVKPLRTNEL
LNLWTHMWRRRCEG
>GrARR43
MMEMRNTNGVVAEELGTLEGDDLKVDKIAQNVKDCHVGVVQAPAVLQIQQQQPQSASGCW
ERFLHQASIKVLLVENDDSTRHVVAALLRNCRYEVIEAASGLQAWKILEDLTNHIDLVLTEVF
MPCFSGIFLLSKIMSHKTRKNVPVIMMSSHDSIGIVFKCLSKGAVDFLVKPIRKNELKNLWQHV
WRRCHSSSGSGSGSESGTQTQKSEKSGSVENSDNSGSNDEEDNENNGLNVGDGSDDGSGTQS
SWTKQAAEIKSPSPVSPQDRVAECPDSTCAQVIHSNAEASGNKGVPATAPRGCQELDEQLDDV
PMGKGLDIRMAGSVDLQRERPVEVPIKTIGANQINLLEMSFNKLNEPIDKRQLDLNTKSSSGEL
NSEAAHQTDITSKTNDLKKESTEYEASNRISKISDGNDKTTDDSKEVLPSTELGFKRLRGAEDS
EAMLRDERNVLRRSNSSAFSRYNMASNANKFSFVNTGSSSARDSKLELTRKRSVCDVQSPLV
NDLPNQYSNVGSNNINMASTTDNAFAKPAVLKNKSASSSTFRLGHPSSAFQPMKNDLLNAAR
KPVLDKADGVTTKAGLKQPRLTHQELDMQDRLQHQQPTDHDTLSLKKMAADAPHCGSSNVL
GGPVPVEGNAGNYSVNGSNSGSNHASNGPHGSSTLADTVGTNIESDNGIAGKSGSGGSGDAS
GTGSGRGSGSKVDQSKSACREAALTKFRQKRKDRCFRKKVRYQSRKRLAQQRPSIRGQFV
>GrARR44
MEEKRRSNCGEGFSVLVVDHDTTSLMLLTSMLQLFSYQVTTTEVESVAISLIEEAKDRFKLVM
ADVNMDDVNSLSFLRKLLKMNIPFILMSKEKGHKAAEKAVAYGASLHLEKPISKHDLKYLWQ
YAYGRPAKRAKYLHGNELRTYDPNKNVGERDEQKKMLNIMPPTEKKPRLLWNEELHQKFIA
AITALGYANARPKSILTMMDEPNLTQRQVASHLQKHKEHMKRLMSKASPSELVSNANASTSI
YGKPGFLYMPQGYYSVLSQLGGHTHKYLNDSVAGTCQFSYLPQHSTADHFSSEYHNEETLQF
PNILESMHEKQNIIPGPSPSGNLVYENHNWEPWTEAARGVAEAEIGVGNAAGGVTWNQQSPE
LAQLLKHLEEEGDDCGDCANQPHPDAVDQFCEWLKEAIQGNDNNP
>GrARR45
MAISVESNMNNVYAGVTVLLVDGDSTCVIILSKMLRSFGYKVVTTKRATDALCIIRDQQHKID
LVLTEACLHDMDKYELLETIRNISSLPIIVMSTDYDRNAVLGSLFKGAALHLEKPITMDDIKNL
WQFTLIKGREINVPIIEAKSCIKEVSSMESALGVVVDGRRNLRDEKRKRPLEVENDKEGDNWD
QGSSTLKKPKLIWTNELHNRFLQAIDMLGSEAYPKKILQLMNVPGLRKENVSSHLQKHRLSLK
RQQEAILNTISSTESQAASHHALSEFSPRNGFHLFTDTTQTTSVAEQHGYINGLVQDNLNG
>GrARR46
MLLTNLSRVRIPKPTNRIYKQECCLSFDSPLYGSQAILRQEVSIPVRFYSKEASPLHKPKSCEFQ
AHKVAAIPNGLKAWEMLKGRPHDVDLILTEVDLPSISGFALLTLIMEHDICKSIPVISMI
>GrARR47
MICTTNDLSAWKDFPKGLKVLLLDEDSNSASELKSKLEAMDYIVYTFCNENEALSAVSSRPES
FHVAIVEINTNKKNGSFKFLETAKDLPTIMTSNIHCISTMMKCIALGAVEFLKKPLSEEKLRNIW
QHVVRKAFNAVGTDLSESVKPVKESLVSMLYLQLENGEPKNKDLDKTQDASVIHENDPEPST
GSDKYPAPSTPQIEQGGRLSANGDCQDHANCSIEKESSEEDGESKSVETTSDNTIAEVTIPVGQP
QGPRDTMVTEEADLVDGTKGKSTTYSQTENGVNSKNSQAVAEKPSTVSGIHSSCLNKANRKK
SKVDWTPALHKKFVQAVDQLGIDQAIPSRILELMKIEGLTRHNVASHLQQKYRMHRKHILPKE

DDRRWPQRDQTQKSCYPHKPIIAFPSHYSNHVVPVGPLYPMWGAPPYPSSIQMWGSQGYPLW
QPTESWQWKPYPGVHADAWGCPVMPPPHGYSSAFTQVSSYQNASVFHCSGTMDNRSGMPQ
NSVEHQPAEEVIDKVVKEAINKPWLPLPLGLKPPSTDTVLAELSKQGISTFPPHINGLNSSCHGT
T
>GrARR48
MGDFVVMSCEEVKEVKGNSDTEMENGEFEGIKKKKKKQQQESCVYGEVVKWERWMALRV
LLIEADDSTRQIIAALLKKCNYKVMSSQDSVSTVYKCMLRGAADYLVKPVRRNELRNLWQHA
WRRQSSIIGGNCPGDESIGRKRVEATSENDAARQTDKGSDTQSSCIKPDMEAESADMETIQEFS
DLIKGKSQPSGSQMHEAHDCLNQNLLMHETKTSVNACKDPNLTTAYKGVELECQRTNMNISV
EAGNALADSPREAIDFMGTFNRNFNFSSINSTSKFDSSPPLDLSLRRCNNNDFENNVTRERPILW
HPNSSAFTRYSNRLSQPQHSTLTSISNKKKESGSNSETILSNIVSEHDAATPSPTVTSQRNMIPST
AGATDKSRHTEAATSCMEQRECPSSVNQHEPTFGVNPFHHSSLEMNSSRQFYDRLASGTNQM
DQKLDSVEDKGHISPTVNQSGTGSFCNGSISQLNGIAYGSSSASNSNVDQVIVRASTERKNDDN
VPAPAGNSHRSIQREAALTKFRLKRKDRCYEKKVRYESRKKLAEQRPRVKGQFVRQTQVDHP
TQIQAECHYHNSPDG
>GrARR49
MVSTRSVNSPAMKITILVVDDDSTSLAIVSAMLREFRYEVTSVKTPAAALSVLRSNPSIDLVVT
DLHMPGMNGIELQKRINKEFKLPVIIMSSDDDENVMLESLAGGAVFFIVKPVDPVGLKNVWQ
YAVAAKKGKSLLIEDMDRESSSSSPADGKLSLGGNTKSVSSVNNEKNDPKNGSKRKGASGKG
RDKDDNDDESKSPPPKKTKKHKIVWTNTLHNQFLEALRQIGLERAVPKKILERMNSTGLTREN
VASHLQKYRIFLKRLAERGCFASKAVIDRFLKSNFAAGHPLLLKTAQEYSRLEHMERLRVLAS
YPGLRESLMGHSSTGSVPLFYGHPGASSSNAAQQPLGYGQSRLLSNQTNQPLFPGSGNMLNNP
YLNNRVGYGNGIGIGIGIGIGSSSSVNGGGGFSSGLLNGGNSSLTYPNQVQARPDFYNAGPSSSSP
FRFGSAGFHSSGSTLGNGLFGSGSSSYPSLNSSSSGSTVVNGLFGSSSRSHPSLNSSSSGATLGN
GLFGSSSSSYPSLNNSSYPSLNPGYTNNAANSYRGMRFYEHLLNGSAPPLTGDYGSMNQTRPT
GDYGSMNRTHNENINVPTMRTTPLDSLDFMREFPIPPVDSSTLIQGLGTGNTRLTEINSDQLLN
NVPNLGNEPRGGDGLLQDLVLESKKLANKEKAGEQSVENSDYCLPSLFPEIYPTLDELLNCDF
PDPLSDEDNHPWSEEAIGQVQSELEELINNPNPAAGDGSNESNPVTNKQQTGGEQVTVTHPLS
NVLQVMTAPAPAPASAPAPAPAPAPAPAPALAVYNSANNANNGAPVGANSSSYEDDEDFLDS
LLNFNDEFE
>GrARR50
MVMAASETKFHVLAVDDTLIDRKLIEKLLKTSSYQVTAVDSGTKALEFLGLNNNDHQDVKVN
LIITDYCMPGMTGYDLLRKIKESSSFKDIPVVIMSSDNIPSRINRCLEGGAEEFFLKPVQLSDVNK
LRPHLMKRTQTNVNKRKAMDEIVSPDRTRARYNELEVN
>GrARR51
MIDIECFSPISLGFRSLNPPIVSTSKNAASPSILRISPNHASFKLTKVIESKCTIGYVGFSLIFYFCTIS
ARLTLKFVAIPDGLKACEMLKGRPHNVDLVLTEVDLPSISGFALFTLIMEHDICKSIPVISMI
>GrARR52
MFRNKNLSVSIVDDCRLTRRFYEMHIKKFGVKVQAVENGKQAVDLFRSGTSFNPNIKDQDMP
VMDGLEATKQLRGMGVNCRIDGVTSISSQDESSLAKCKVSWCR
>GrARR53
MTVEQIVSEGKDQFPIGMRVLAVDDDPTCLLLLGTLLRRCQYHVTTTSQAKTALKMLRENKN
KFDLVISDVHMPDMDGFKLLEHVGLEMDLPVIMLSANGDTKLVMKGITHGACDYLLKPVRIE
ELQNIWQHVVRRKKKDRCNSGSKDKPHPDSGEAAGIGNVDNNGKLNKKRKDQNEDEDDER

DENGHDNEDPSAQKKPRVVWSVELHRKFVAAVNQLGIDKAVPKKILELMNVEKLTRENVAS
HLQKFRLYLKRISCVANQQANMAAALGTADSAYLRMGSLNGLGNFHTLAGSDQLHNAAFRS
FPPSGVLGRLNTPAGLGIRSLPSPGTIQLGHVQNSGNPTNDLSKLQSFVPGNHNTNILQGMPMS
LELDRLQHNKSVGHIGELPTTDSTTVFPGSGSLVDARITGFSNNPLLGVTSNSLMLEGSSQQAT
SHTSVSAIGFQNGNALSDFTSIAPASNQLQDSKADSQGQASPINCNAGQIIRSAPQEWNAPRKD
APYQSHALINSSIPINSAMIQLGQCLDRNNSIFHRTTDLDSVGPLNFVDPLSIKHSEGDNYIMEPS
VIEKEGYLMFQPRPHGSHVPDNTGSLKDLASAMMKQEDDFGCNGYSLRTSI

G.barbadense

>GbARR1
MKNSIGGKGSMSTASSITTWKAGDVVSVPDQFPAGLRVLVVDDDPTCLIILEKMLRNCSYDVT
KCNRAETALLKLRENRNGFDIVISDVHMPDMDGFKLLEHIGLEMDLPVIMMSADDGKDVVM
KGVTHGACDYLIKPVRIEALKNIWQHVVRKRKNEWKDLEQSGSVEEGDWQPKQSEDADYSS
SVNEGNWKSSKKRKDDEDETDDRDDTSTLKKPRVVWSVELHQQFVAAVNQLGIDKAVPKKI
LELMNVPGLTRENVASHLQKYRLYLRRLSGVSPHSCNPNNSFMNPQDETFGPLSSVHGFDLQT
LTATGQLPAQCLATLQAAVLGRSTAKSSIPMPLVNQRNIFSFENPKLRFGEGQQHVNNNNKQV
NLLHGIPTTMESKQLTSLRHTSQSIGNLNMQVAPHGAQSRQNNSSLIEMGQPLSRVQILNDSTV
PLSVGQPIVPNGIAANVSTRNGIPENIRAPGYNLVSQTSSILNFPMNHASELPVDTFSLRSTLG
MSDHTSKGAFHEDFNSEIKGSGGFLPSYDVFNDYQYKSQNWELQNAGMILDDSQHSNSLQGN
LDLTQSVLVQQGFPSGQINGQSRSVPIVSKAMFSAGDSTEPGNLLNVNHHLNTIRADNTVRVK
SESVADGNPSNLFTDHFGQEDLMIALLKQQQGIAPVENEFDFDGYSLDNIPV
>GbARR2
MNSSNGNGSMSTASSSGAWNGRDQFPAGLRVLVVDDDQTCLMVFEKMLKTCLYQVTKCNK
AETALSVLREHKNEFDIVISDVHMPDMDGFELLKHIN
>GbARR3
MVCTANDLSTWKDFPKGLKILLLDGDTNSANELKSKLEAMDYIVYAFHDENEAILAVSSRPES
FHVAIVEVSTDNNNGSFKFLETAKDLPTIMTSNIHCISTMMKCIALGAVEFLKKPLSEDKLRNI
WQHVVHKAFNTGGNDLTESLKPVKESVVSMLQQQPENLKPKNEDSEKTEDASMIHENDSEPP
AGNDKYPAPSTPQLEQGGRLLDDRDCQDHTHCSIEKESGEQDGETESVETTSGNTTATGGQ
PQGPSETNVKEEDDSVDGTMGESPQNRVDSKGSDVVAEKPSSCPDKANRKKLKVDWTPELH
KKFVQAVDQLGIDQAIPSRILELMKVEGLTRHNVASHLQKYRMQRRHMLPKEDDRRWLQRP
QMQRICNYPHKPIMAFPPYHHVPVGPVYPMWGAPSHPPTIQMWGTQGYPPWQPTESWHWKP
YPGVHADAWGCPVMPPPQGYFSTFPQNASGFQPCNVDKRNGMPQTLVEHHPEEEVIDKMVK
EAINKPWLPLPLGLKPPSTDSVLAELYRQGISTVPPHINASGR
>GbARR4
MESKELNLNKELKAGGAGNGFIDRSKVRILLCDNDTKSCEEVFSLLLKCSYQVTTVRSARQVI
DALNAEGPDIDIILTEVDLPMTKGMKLLKYIMRNNELRRIPVIMMSAQDEVSIVVKCLRLGAA
DYLVKPLRTNELLNLWTHMWRRRRELGLSEKNILNCDFDLVASDPSDANTNSTTLFSDDTDE
RSRKSSNPEMGISTHQEDESAAATVEPPQSDSPECRPDVPGISDRRTGQFSSVPKKSELKIGESS
AFFTYVKSSAVKTSSTQVATPNHESAAENKIGEEHLPQPGEQVVSDTRVHENGETWENNSQG
DEFRSSSSVPDSLSLERSSTPASMEFSQQRDFKEDKFSPALVPPSNETQHDVSGLPTQSPYLHYM
PGVLNQVMMPSSTQLFQNNLHDIHNHTSSPLVPQYNHLQQCLPHPHVSGMASFPYYPVNMC
MQPGQMPTGHSWPSFGNSSSNEVQPSKVDRREAALIKFRQKRKERCFDKKIRYVNRKRLAER
RPRVRGQFVRKNGATVDLNGQPASADYDEDEEEEQASRDSSPEDDTSGC
>GbARR5

MATMHRVVQSSVSTSDATTTSYGGLTSCKAADIVISDQFPAGLRVLVVDDDITCLKILEQMLH
RCRYHVTTCPQAKVALNLLRERKGCFDVILSDVYMPDMDGYKLLEHVGLEMDLPVIMMSAD
GSTRAVMKGIRHGACDYLIKPIREEELKNIWQHVVRKKWNENKELEHSGSLDDTDQHKRGHD
DAEYGSSANDATDTSLKPLKKRSNSKEGDDGEIDNDDPSTSKKPRVVWSVELHQQFVSAVNQ
LGIDKAVPKRILELMNVPGLTRENVASHLQKFRLYLKRISGVAQQGGIANPLCGPVEANVKIGS
LGSFNIQPLAASGQIPPQTLAALHAELLGRPAGNLVVAMDQSALLQATPHGPKCIQVDHGVAF
IQHLVKSESSSSKHFSQSFAPVEDVASGFRSWPSNNIDTAGPSNSGGLSTQNGNMLIDLLQQQQ
QLQKPQQRSTVSELRRSINVQPSCHVVPSQSSASFQAGNSPVSVTQNGSYSRTAVIDYNFLSSQ
SNCPSLNIGQVSDGNLQTTGVLSGYIPPASVSPSVSSCSVNADNCASQQVQTSSITFKASRRLPG
FVHSTSNIPDPYGSTKSGDLLNQEPFNNLGYINKGTCLPAKFAVDEFQSHLSSSSHGKVFSENIG
SRVKQEPSMEFGDNAKVGIPMLQQFPPNDLMSVFTE
>GbARR6
MVLAESGFSSPRNDAFPAGLRVLVVDDDPTWLKILEKMLKKCSYEVTTCCLAREALNLLRER
KDGYDIVISDVNMPDMDGFILLEHVGLEMDLPVIMMSVDGETSRVMKGVQHGACDYLLKPIR
MKELRNIWQHVFRKKIHEVRDIESLEGFESIQMTRSGYDLFDDGHFLSGDDTTSGRKRKDADN
KHDDRELSDPSSTKKARVVWTVDLHQKFVKAVNQIGFDKVGPKKILDLMNVPWLTRENVAS
HLQKYRLYLSRLQKESDIKNSFIGMKHSDLPSKDSTASFGPHKAMNMIPDDVPNSTYSFSASNS
QAQNVDLKGQGDLKGITSAPMAEPKGALSVDIRDSHEAKSTQMSFDHSLGSVDSAVSFASFNS
TTPLQYPWTEIPEIQFKQECESLHLENGFSQLPLPGPSMIENEANRSRIEVKPLLDDCRSNFVEHL
GPVGAEDLFPIQSKSQSLNNQVFDLISATKSSMKTQDVGLNYLADSEFALRNLNASGVGVPLA
TLSEDLQICWLQGDCYPMNYGLQDLECSTYDNPALMAETPFHLYDVLRFDHEHLFDPAEYYA
IDQGLFA
>GbARR7
MARNGGVAWMRTTEKIDGYDLSTSDTEEVHVLAVDDSLVDRKVIERLLRISSCKVTAVDSGR
RALQYLGLDEEIQKKETNGFDGLKVDLIITDYCMPGMTGYELLKKVKESSAFREIPVVIMSSEN
VIARIDRCLEKGAEDFIVKPVKLSDVKRIIDYTTTELREGEQRGARARRRGINKRKQREGDDDL
SSSPPSTLSSSTSSSPSSSIQSATAPLSPSTLVSPTRRLKMTSSE
>GbARR8
MEEIFLENLIEPQIDDDHTLKELTVFAFNTQVLSLQYLCAVLHNCNYKVKSASSAVEVIEILRTN
KHGIDIVLVDVDTADLNAFKVMETIGLETNLPVIMVTADSNLENITKGLAHGAVDCIIKPFEME
QIKNTIQSHVASNKTRGQNLNPLPGSNARMAVKDACCSSKRKKKRLVWSRELDAKFVKAVQI
LEKGSENVHPKRILDVMNEPGLSRAHISSHLQKYRLALKKRKADTNKQGLEVEPTNYCLKKR
EIGNFNADRRRLAIQSFNGVMSPSSSSPDQKKQSQSLVYSCIDGHGPEFQTPDFYYNNHRLETN
IQPHNVESEHVSGTTTLSPYFNGVDSEPIILSSAAVYPSPNAVFLEPEANIAFHSHFDVISVPNPL
YGFTIDCSTEVQYPSSTVLGRNSETYAVQNGTTISPSDTSSYHYVEPENEVSGTYNSQIECSLFE
EESIDWYSLLDDQ
>GbARR9
MTLQEKRGGSNGEDGGKDRFPIGMHVLAVDDDPVCLKVLENLLRKCQYHVSTTNQATTALK
MLRENRNRYDLVITGVNMPDMDAFKLLELVGLEMDLPVIMLSTHGDTELVMKGITHGACDY
LLKPVRIEELKNIWQHVVRKNKPDFKDHINALNQDNGHEDEDRSIQEKPRVVWSDEVHRKFV
SAVNELGLDKAVPKKVLDLMNVEGLKRETVASHLQKYRLYIGHSIVATLGSKDPSYLRMGPL
GGFGYFHTLTGPGRISSASLPSYQPGGMFGRLNTSATLSLHGISSSVIQPGHCQTSNNPINGSGKI
QPAVVPANQKKNGTLFQGIPTSVDLNQPSQNKPTNGCGEFNRVNDPNFQDARMVVGGSCNTL
HVSSGNPLLLQSNTQQTKHSGAFGNQASLSNSLLDLCSFKAKSANLEDSKGDISNVGLNNYIIL

NMEYATKQQRGDSRHDNNGNTNHSFCRADSLVPFLFVCFVLFCCVYKICQKIYDAWC
>GbARR10
MTMEEKLGGSNGEDGGRDRFPIGMRVLAVDDDPICLKVLENLLRKCQYHVTTTNQAITALKM
LRENRNRYDLVISDVNMPDMDGFKLLELVGLEMDLPVIMLSAHSDTKLVMKGITHGACDYLL
KPVRIEELKNIWQHVVRKKKPDSKDQINASNQDKSRGGTGETGPTSSSSDQKVSKKRKDQSED
EDEEGDDNGHEDPSTQKKPRVVWSVELHRKFVSAVNQLGLEKAVPKKILDLMNVEGLTREN
VASHLQKYRLYLKRLSSVATQQANMVAALGSKDPSYLRMGSLDGFGDFRTLTGPGRISSASLS
SYQPGGLFGRLNSSAALSLRGISSGVIQSGHSQTLSNPINGFGKIQPAVVPANQNQNGTLFQGIP
TSINQLSQNKPTNHFGEFNRGNDPNAFGVATNFQDARVTVGGSSNTLPVSSGNPLLLQSNTQQ
TQHSGAFGNQSSSLGVTSLNQEPFDMNVRGSSNFLDHGRCSENWQSAVQLSSFPSNALSTSEA
FSHEQLPNNNLQESISWTSSHLSSSPLDLSSSMANPANLEDSRGNISQVSLNNNVIQNIDYTTKQ
QWGDSRHDYNGNMNNSFSRGDSLVPASGPMMDQSNLISDKMNDVSLFSQFSGDSTYVVPHL
DGEKSAFDTKPRSNEDFLFETKPQNGFNQNSFEPLENIMMSMIKSDQNNETPLMDGEFGFDAY
SLGSCI
>GbARR11
MRVERVISDENDSFPVGMRVLAVDDDPTCLLLLETLLTKCKYNVTTTSQAITALKMLRENKN
KFDLVISDVHMPDMDGFKLLELVGLEMDLPVIMLSANGDTKLVMKGITHGACDYLLKPVRIE
ELQNIWQHVIRRKKFDRKDRNGSGSQDKPYIDSSEAAGLGNVDQNGKFNKKRKDQNEDEDEE
RDENGHDNEDLSTQKKPRVVWSVELHRKFVAAVNQLGIEKAVPKKILELMDVENLTRENVAS
HLQKFRLYLKRISCVANQQANMVAALGSVDSTHLRMGTMNGLGNFHTLAGPDQIHNAAFRS
FQHSGVLRRLNTPSGVGISGLPSSGLVQLSPVQNLGNTSTNQSKLQSFVIPGNHNANILQGMPM
SLEFDQLQNNRAASHVGQFPSVDNTTVLPVSSISASLIDAGCSSSPLLGVTSNSLLLEGSSQLPT
LHARNLRDIVSGFPNRSTLSDFTSIAPASNQLHGSKADIQDQASPISCNAGKIISSAPQDWNAPK
KDAPYQSNVPSCSINSLRAVNGDMAWLGQCLDQNNPIFNRNVGFDFVDPLSIKFSEGENLAME
PSVIEKEGYLMVQPSRQGSYIPDNEDENFAMEPSVIEKEGYLIVQPRRQGSYIPGNLGSLEDLAS
AITKQDHDKGKPTDGDFGYNDYSLRTCI
>GbARR12
MCHEQKEARHGVVRDGQGSGSIGENGSRIVERTLNVNNGSLEAIEVHDVSEIPQRQPRGSMIR
WERFLPFRTIKVLLVENEDLTRHLVSALLQNCSYEVVAVANGLQAWKLLEDPTNHIDIVLTEE
DMPVLSGSDLLCMIMNHKMLKNVPVIMMSSHDCINLVFKCLSKGAVDFLVKPIRKNELGNLW
QHVWRRCHSSSGSVSESGTLSKKSIKLKVNDEPENYAANSDEHDDDSDVPVGCNGSENGSGT
QSSWTKRAAEGESSQPMSSLNRFPGAPNSTCAQVVHVKHEKRRSPWTCVTQRKECQEQHEQL
LDATEGKDLEVRVESNHEWQCGNQCKNSPTHLAEAASKTFDRGWFEHQDENITGKDRTPDII
ATLQQAECRASDAPGGPSDVPQLKDGACHGSEEKLSFELTLKRWQGASDGRNAANDGHNVL
RHSDSSAFSKYSTASSAKQALTGNVGSCSPLDHSSVTKKTEIMCTFPSHSNGILLNQSSVGSNN
KNDMTTTAKFVGPKPKALVDKSGSISTFKCLHSSSFQPMHGCCICSSQEVSPETVGHDTSLKIM
AITDKQCRSSSSVNGSASGSNYGSNGHNGSETGLRAEHAVMEDGNGTASGRSGGSGANEDRV
AQRAAALTKFRQKRKDVLKRRYGFRAGRSWQSNDLV
>GbARR13
MELMKSENIVQDRQPQPQEEEEIMQQEEEEEQRFHVLAVDDSVIDRKLLEKLLKASSYQVTCV
ESGEKALEYLGLLHHSSPASSSSQHHHQGHKVNLIMTDFSMPGTSGYDLLKRIKGSSWKDVPV
VVMSSENVPSRISMCLEGGAEEFMLKPLQLSDLHKIQAHLLKSLPHS
>GbARR14
MLYQIMDGDYGGSCGGCSEDMTVEFGYEPHVLAVDDNLIDRTLVEKLLKNSSCKVTTAENGL

RALEFLGLGSDQRNSLEATVSKVNLVITDYCMPGMTGYELLKKIKESSVLKEVPVVIMSSENIP
TRINQCLEEGAQMFMLKPLKQSDVKQLKRHLMKYRS
>GbARR15
MRNEDRSETSYERRTKKLKFLESQAEFTAVSNGLLAWKILEDLTNHIDLVLSEVVMPCLSGIGL
LCKIMNHKTRKNIPVIMMSSHDSMNIVLKCLSKGAVDFLVKPIRKNELKNLWQHVWRKCHSS
SCSGSGSGIQTQKSTKSKSGGSDNNTGNNKEDDIGSVGLNAQDRSDNGSGTQSSWTKRAIEVD
SSQPISARDQVVHSRSEVLGNSWVPVTITRECDSRDDELDNAVKGKDLEIGVPKITALQLENPS
EKVNTNAAGGNQEKLSELNPSKDDEKLEKAQLELTGEKPGVDLVNRAADVIGVISKNTDAQI
ESAVFDIPDGLPKVSDTKGKVIYKTKEMPSLVLSLKRLIDVGDSGTSAHERNVLRHSDLSAFSR
YNSGSTANQAPIGNVGSCSPLDNSLEATNTDSMKNFHSNSNNMPPNQQSNGSSNNNDMGSTT
NNAFSKSAVLNDKPASKTSGPSSAFQPVQKGHATAMQSPAEDKADAAIGKKILTKAKGTDQQ
VQVQHHHHHYYHYHHHVHKMPQNQKLDNQDDLCQCGSSNMSSAPHVEANAGNHSSNGSA
LESNHGSNGQNGNITALNSRGLNLESENGLLGKGGTVGGIGFGGSNGADQNRFSQREAALNK
FRQKRKERCFEKKVRYQSRKKLAEQRPRIRGQFVRQVPENKNKDTNC
>GbARR16
MGNMATQTQFHVLAVDDSLIDRKLIERLLKTSSYQVTAVDSGSKALEFLGLNGENEDEERNSS
VESVSAADEDHQVGVNLIITDYCMPGMTGYDLLRKIKQSSSFKDIPVVIMSSENIPSRINRCLED
GAEEFFLKPVKLSDVNKLRPHLMKGITTKTEMQSNTNKRKGSEEIQSPDRTRPRYNELEVV
>GbARR17
MGEVVVTSEEVEEVKIDSETEEQENGEVEGNMRRRRRKKKKKKKKPGCSASGAVVNWERF
LPMPALRVLLVEADDSTRQIISALLRKCSYRVAAVPDGLKAWEMLKGRPHNVDLILTEVDLPS
ISGFALLTLIMEHDICKSIPVIMMSSQDSVSTVYKCMLRGAADYLVKPIRRNELRNLWQHVWR
RKSSIVGGNSPQDESIGQKKVETTSENNAASNHSIGCLDGVRKNKEQTEKGSDAQSSCTKPEM
EAESAQKENMQEFSHLIKVNSPPIESQKHEAHGSFNQNLLMHEMETEVVDSCKDAYTTLYKG
VELENQRRDTRVLVEAGDALVDSPREAIDFMGTFNKNCTSSSINSAKKFDSSLFLDLSLRRSNP
NVFENHVTQERPTLWHPSSSAFTRYTSRVSQPLHSTLMSFSDQKKDSGTNSEKMLTNVMSEN
NSDTPSPTLTSQRNTNPLTIGATVELKQTEVATPCTQHRLFPVPVPVKGIRLNNPCNGYNTIIPP
MFCARSSSSMAPSPSTANQQEPAFHLNLFRHSSFEANSSGQLYDRLASNTNQSTSQLLHKLDQ
KLDSIEDRGHISPTTDQSASSSFCNGSLSQLNGVAYGSTGASNGNVDQAAVTRASTESKNDDSF
PSPSGKPCRSIQREAALMKFRLKRKDRCFEKKVRYESRKKLAEQRPRVKGQFVRQVQADPMH
IETEHHYGNSSDG
>GbARR18
MELMKSSDNIIQDRTQQQHKQEPEMQAQEGQQEQGHHFHVLAVDDSVIDRKLLEKLLKVSSY
QVTCVDSGDKALEYLGLLNNLDSDSTASSSSSSSSSSSSSSSSSSSSSSNSSSCSQSSQREGLKVNL
IMTDFCMPGMSGYDLLKRVKGSSWKDVPVVVMSSENVPSRISMCLEGGAEEFMLKPLQLSDL
EKIQVYLLKSLHISCTNIDKDDDDNNTDDNSDNNNDKGVNNDSNIDKGNNSTSMNNNFSKRK
ALSSEDTESRRPKIKGLAVAI
>GbARR19
MTMEEKMGGSNREDEAMDRFPVGMRVLAVDDDPICLKVLGTLLLKCQYQVTTTNQAISALK
MLRENRNRYDLVISDVNMPDMDGFKLLELVGLEMDLPVIMLSAHSDTKLVMKGITHGACDY
LLKPVRIEELKNIWQHVVRRKKPDSKDRVNALNQDKASGGIGEAVQTSTSCSDQKFNKKRKD
QNEDEEDDGEDNEHENDDPSTQKKPRVVWSVELHRKFVAAVNHLGLDKAVPKKILDLMNVE
GLTRENVASHLQKYRLYLKRLSSVATQQANMVAALGGKDPSYLRMGSLDGFGDFRTLTGSG
RLSSASLSSYQPSGMFGRLNSSAALNLRGISSGVIQQGHPQTLSNSINGRGKIQPVGLPANQNQ

NGTLFQGIPTSIELNQLSQNKSANHFGELNPVNNPNVFGVATNFPDARMTVGSSSSSLSTPSGSP
LLLQANTQQAQHSGAIGNQSSLGVASLNQESFDMGVHGFSNFVDNGRCHENWQNTVQLSSFP
SNSLSTCEAFSHEQLHTNNLQESISWRSSHLSDNPIDLSASMANAGLEDSRGDMQCQISPSNNV
IHNIDYAAKQQWGENSSCAGADSLVSGAPYVQHLEAGKLAFNTKLRSNEDFLFEQRKPRNEFS
QNNFETLDIISSSMIKPEQYNETGMMEGELGYDAFPLGSCI
>GbARR20
MRVERIINDTNDEFPIGIRVLAVDDDPTCLLLLETLLRRCKYNVTTTNQAITALKLLRQNKFDL
VISDVHMPDMDGFKLLELVGLEMDLPVIMLSANGDTNVVMKGITHGACDYLLKPVRIEELNN
IWQHVVRRKRFERKDRYNSDSQDKLRADSGEAVGMGSIGSNGKLKKKRKDQDEDEERDENG
HHNNEDPSFRKKSRVVWSAELHSKFVAAVNQLGMKNATPKKILELMNVQKLTTQNVGSHLQ
AFIRTFSAHSYDEPKKFRLYLKRISCAANQEVNIAATVRMRSPNGLGNFHTLAGSNQLHNAAF
RSFPPRGVLRRLNTHAGLLIRDLPSPGTIRSGHGQSSVNSGNDQCKLQSFVSGIHNANILQGLP
MSLELDQVQTNNGVSHIGELQIADSTTVFPVSSSLIDATITGFSGNPLLGVTSNSLMLEASSQQA
GNSRDIVPAIGFRNGNTLSDLAPIAPASNRESNADLQREPISINCNARELITSAPQEWNSPYQSN
VTPCSMNSSIPVSGTMVQFGLCLDQNNSMDSDSIGPSSFIDNSAMEQSIIDKEGYLMLEIWGQG
SHIPYNIGLLEDLWELQ
>GbARR21
MMYVVGIKVNATTYTVCFFNAMNGGFDGDFPAGLKVLVVDDNRTCLLVLETMLRKLSYEVT
TCQLARHALALLREDKNRFDIVLCDLHMPELDGLKLLEIIGLEMDLPVVMMSSDDGKGVIMK
GIIHGACDYLVKPVQMEAVRLIWQHVVRKRQRALEDSQQLRGNIHATGRTLLLKQAKNAVD
QMPARERRTLKRARENEDEDDEGELSEEVTTAKKPRVIWTQELHDIFVIAVNQLRQRMNNSLL
PYLYHSLMEINMLITCLPEAVPKKILERMQAMNVTGLSRANIASHLQVNISYIYTVFKNHIIINE
VTYNLQKYRLHLRKGGGQPLADNRDVNLNPSIGQASSFNQFNLQFQQPTAIGSSCNQLPMQN
LMITPQPCTINDSVVPDSTQCFPTELSTNDLSQPNLLFQNDVPTSNVEHLYRNVGVNVSELSNPI
SSVDDSSVDQLIYEPSFLVRQYDPEDFFHGGFPRSDSYVSVRDIEYLTSEEAPMKRISFFLR
>GbARR22
MSGNGASSSSLNMISGEDVVNNNLTALVVDDSPLLRLLHEIHLKKYGLKVQVAENGKVAVDL
FHLGASFDLVLMDKEMPVMNGVEATKELRAMGVTSMIVGVTSKDGPGEQQAFMEAGLDYC
FEKPLTPEIISILLEELNKHNKKD
>GbARR23
MVCTRNDLSAWKDFPKGLRVLLLDEDTNSAAEIKSKLEAMDYIVYTFCNENEALAAVSSRPE
SFHVAIVEVCTNSNNGGFKFLETAKDLPTIMTSNIHCISTMMKCIALGAVEFLRKPLSEDKLRNI
WQHVVHKAFSVGGDDLSESLKPVKDYVASMLHQQLENGECKHEDLDKIEDASMIYEKDHEP
SAVNNKYPAPSTPQLQQGGRLIVNGDCQEHTNCSMEKESGEPDGESKSVETTSGHTIAEVIA
LVGQHQKPRETIVTEEADSIDGAKGERTVDSHPQDRVHSEDSCAGADKPNAVSGLHSSRPNKA
NRKKLKVDWTTELHKKFVQAVDQLGIDQAIPSRILELMKVEGLTRQNVASHLQKYRMHKRHI
LPKEDDPRWPRRDQTQRSCHPHKPIMAFPPYHSNPVPVGPVYPMWGAPHPASIQTWGSHGYY
HPWQPTESWQWKPYPGVHADAWGCPVMPIPQGNCSTFTQNGYGFHCSSTMDNRSGMPQNSI
QHQPAEEVIDEVVKEAINKPWLPLPLGLKPPSTDSVLAELARQGISTIPPRNQRL
>GbARR24
MAQSKHGGRKLGQSNKMTALVVDDNMINRTIHHRLLDNLGIENEVVSNGKEAVDIHYSGKM
FDLVLMDMDMPIMNGIEATKKLREMGIRSVIAGASSRAMEEEIQEFMEAGLDDYQEKPLTMP
KLVSIIHKING
>GbARR25

MEEKMGGSNGEDSGEDQFPVGMRVLAVDDDPICLKILANLLCKCQYQVTTTNQAIIALKMLR
ENKNRYDLVISDVNMPDMDGFKLLELVGLEMDLPVIMLSAHSDTKLVMKGITHGACDYLLKP
VRIEELKNIWQHVVRRKKPDSKDELAAPNHDKARGGTGEAGQTWVACSSDQKVNKKRKDQS
EDEEEETEDNGRENEDSSCQKKPRVVWSVDLHRKFVAAVNQLGLGDQAVPKKILDLMNVEG
LTRENVASHLQKYRLYLRRLSSVATQQANMVVALGSRGPSHLRTGSLDGFGDFRSFTGPGRFS
SASLPSYQSRGTFGRLNSSAALTLSGISSGVIQPGNSVNGLRKIQPVVFPANQNQNGVLFQGITA
SIELNQLSQTKSTNHFGEYNCVDERNVFRISSSFPDARVVVGSSSNSLSTASGNALVLQGNTQQ
VQCSAAFENLPSFGMTSLNRESNDLSVRGSSNCLEHGRCSENWQGTVQLSNFPPSNEQLPSN
NLQESIPWRNSSPSNGCIALSSMASSAFLGDSRADMLCKAGLNNSYSHVDSRVSASGSMMDQS
NAVSSNTNDVSLFSHLNGEAPFAVWLSEGDKSSFDTDLRSNDNFLFLQSKPQNGFSQNNFESL
EDIMSPVFKLEQNNETAFMDGGFGFDVAYPLGSCM
>GbARR26
MNVDGDGDKGLREQNHRLYDGSKRTTNGVVAEEHVMLEDVKVNKIAQNVKDGHVGAVQA
PAMRQIAQQQSQNAMCYWQRFLHLTTVTVLLVENDDSTRHVVTALLRNCCYDVVEAANVL
QAWKILEDLTNHIDLILAEVGMPSLSGLVLLSKIMSHKTRKNVPVIMMSSQDSMNLVFKCLSK
GAVDFLVKPIRKNELKNLWQHVWRRCHSSSGSGSESGTQTQKSVRSKSVEKSDNNSGSNDEE
NNGSIGLNIGDGSDDGSGTQNSWTKQAIEVDSPRLVSPPDQVAECPDSTCAQVVHSNAELSGK
KWVPVAAAKGCQEQDEQLDNVAVGKDLDIGMPRNLDLQLECPVEVPMRTVGAKQINLLDM
SSSKFSEQIGKRQLDLNSESPSNKQNSEAANQTGITSKTTDLKKEIAENEDSNRLSKIPDGNDKT
INDSKEAPSVELGLKRIRGVKDAGTVVRDERNVLRRSGSSAFSRYNTASNANKVPVVNIGSSS
ALDSNLELTRKGSVCDNQSHLVNYLPNQSSNVGSNNIDMGSTTNNAFAKAAVDKNKSAASST
VRSLHPSSIFQPMKNDLLSATQKVVFDKADDVTTTAELAQARGIHHELQMLHPSNHYDQHHH
LTHGMQKQQQRQPPEHDDLSLKKLAADAPLCGSSNVLGGLVEGNAANYSVNGSASGSNHGS
NGPNGSSNAVNTVGTNMESDNGIAGKSGSGDVSGSGSGSGSGSGSGSGSKADQSKSAHREAALT
KFRQKRKERCFQKKVRYQSRKRLAEQRPRIRGQFVRQTVNNNDPASNVNSCDE
>GbARR27
MVTTVESGARALQFLGLDEEKGSLGFNGLKVNLIITDYSMPGMTGYELLKKIKRSSTFREIPVV
IMSSENILTRIDSCLEEGAEEFLVKPVKLADVKRVMNDCIMRGETEGNKRRRVDKRKFEDDNH
ASSSSSSSSSSIPSSPSSPLPSSPSTAATSSPLHSLKRAKLRDHN
>GbARR28
MNSSSGKSYMSTASSSVIWKAGGDVVADQFPAGLRVLVVDDDPTCLMILEKMLKACQYNVT
KCNRAETALSMLRENRNGFDIVISDVHMPDMDGFKLLEHIGLEMDLPVIMMSADDGKNVVM
KGVTHGACDYLIKPVRIEALKNIWQHVVRKRKNEWKDFEQSGSVEEGDRQPKQSEDADYSSS
ANEGNWKSSKRRKDDEDENDERDDTSTLKKPRVVWSVELHQQFVAAVNQLGIEKAVPKKIL
ELMNVPGLARENVASHLQKYRLYLRRLSGVSQHPSNLNNSFMNPQDATFGPLDLQALAATGQ
LPAQSLATLQAAGLGRSTAKSGIPMPLVDQRNIFSFENPKLRFGEAQQHVNNNKQMNLLHGIP
TTMEPKQLAGLHHTAQSMGNMNMPVLSHGSQSSQNNPLLMQMAQPQARGQMLNDSTVGLA
PRLLSPMGQPMLSNGMATNVSTRNGVPENIRAPSYNPVSQTSSMLNFPMNHTSELPGNSFPLV
STPGISSLAPKVAFQEDVNSEIKGSVGFMPSYDIFNELTQHKPQNWELQNVGMMFNTSQHSNS
LRGNLDHTQSVLVQQGFSTGQNRNSTVISKAMFSAGDSTGHVNAQNGNQHLNSLFVDNTVR
VKSERVADAAPANLYPDHFGREDLMSALLKQQDVIAPAENNDFDFDGYSIDNIPV
>GbARR29
MNVSSVKGSMSISSSTSTWKAGDTISDQFPAGLRVLVVDDDPTCLMILEKMLIACLYKVTKCN
RAETALSKLRENKNGYDIVLSDVHMPDMDGFKLLEHIGLEMDLPVIMMSADDGKQVVMKGV

THGACDYLIKPVRIEALKNIWQHVVRKRKNEWKEFEQSGSVEEGDRQPKQSDDADYSSSANE
GNWKGSKKRKDEEEETDERDDTSTLKKPRVVWSVELHQQFVAAVNQLGIDKAVPKKILELM
NVPGLTRENVASHLQKYRLYLRRLSGVSQHQSNLSTIISAQDPTFGSLSSLSGLDLQTLAATGQ
LPAQSLARLQAAGLGRATAKSGIPITLVDQRNIFSFENPKLRFGEGQQQHMTNKQQVNLLHGIP
TTMEPKQLVSLRHAAQSIGNMNMQVPPHGAQSSQNNPLLMQMGQQQQQQQQSRGQILVDST
INHAPRLSSSMGQPILSNGIATNVSSRNGIPENIRAPGYSQTPSMLNFPMNHASELPGNSFALGS
TPGVSILTSKGAFQEDVNSEIKGSGGLMPSYDVFNDLDQHKPQSWELQNVGMAFDTSQHSNS
LQGNLDLTQSALGQQGFSSGQMNGHNRSAAVASKAMFSTGDVKELGSAQNVNQHLNNLLV
DNTIRVKSERVCDTSPANIFPDHFGQDDLMSALLKQQESVASSENEFDFDGYSMNNIPV
>GbARR30
MHSSGGGSGSCSKGMAMELGDEPHVLVVDDSLIDRKLVERLLKNSSCKVTTAENALRAVNY
LRLGNDTLEGTVSEVNMIITDYCMPGMTGYELLKKIKESSVLKEVPVVIMSSENIPTRISQCLEE
GAKMFMLKPLKRSDVKQLKWHLMKCRN
>GbARR31
MLSCGGGSGGCSKDMAVELGDEPHVLAVDDNLIDRKLVERLLKNSSCKVTTAENALRALDY
LGLGNDRLEGTVSEVNMIITDYCMPGMTGYELLKKIKESSVLKEVPVVIMSSENIPTRISQCLEE
GAKMFMLKPLKQSDVKQLKWHLMKCRN
>GbARR32
MGMAIDRDDTQFHVLAVDDSLIDRKLIERLLKTSSFQVTAVDSGSKALEFLGLNNNDADDDEE
YEQRDSNTESVSSDEDQDHQDLGLGVNLIITDYCMPGMTGYDLLKKIKKSSSFKDIPVVIMSSE
NIPSRINRCLEDGAEEFFLKPVQLSDVNKLRPHLMKGRSKDIDSSHKTTSTRLISSGN
>GbARR33
MGEVLMSSDEVKTESEVDRENEEFEGNKRRKIMSGGSFVSGEAVNWESFLPTMALRVLLVEA
DDSTRQIVTALLRKCNYRVSAVPDGLQAWEMLKAKPHNIDLILTEVDLPSISGFALLTLIMEHE
ICKSIPVIMMSSQDSISTVYKCMLRGAADYLVKPLRRNELKNLWQHVWRRQQLIASGNTPQDE
SVGQKKVEAISENNAASNCSSGCLVGGERNKEQTEKGSEAQSTCTKPDMEAEDANMENMQE
FLLLPSNSQEHEVQTNFNQRLLVHEKKTGAVGACKDTEILVEAGDALGDSPRKAIDFMGTFDR
NCNSSLMNSTSKVGSLTHLDFSLGRCYPNAFKNHATREKPTLWHPNSSAFTRYSSRPSQPLQS
ALTSASDQKKESGTDSEKMLPNSITEYNSDTASPTLTPQRNTNPLTTGTTGQLKQTEVAASCTQ
QVILPVLVPSPSLANQKELACCVNPFHHPSFKSNSSGQFYDRLASNANQLTNQPLQKLDQKMN
STEDRGHIYPTTDQSATSSFCNGSLSQLNGIAYGSSAASNNSNIEQVAVVRASADSKNDDVFPS
PSGNTHRSIQREAALTKFRLKRKDRCFEKKVRYESRKKLAEQRPRVKGQFVRQPQADPSHYG
NSSDG
>GbARR34
MVEVGMEVDVEVGTAEETKKEEEEEEEENDESSEVVRWEKFLPQMVLRVLLVEADDSTRQII
AALLRKCSYRVVAAVPDGLMAWETLKDRPHNIDLILTEVELPSVSGFALLTLVMEHDICKNIP
VIMMSSEDSFSMVLKCMLKGAADFLIKPVRRNELRNLWQHVWRRHMLAGGCANHSLPAKE
HNVEDTAENNAAENNQSSDYGSSTQKIKDESDTQGLSQWKCMSSNVSDTGREQQGNSVKPG
QESLQNEGQAEGISDKLFVSENSERFVEVAHCSGTCESIALKLEENSAFVDEMAHNVVGLQSD
KRIAYVMIGVGCNDELRESSTGAIDLIDSFDKQPMDTFAISSLSDGANKLEFSPQLELSLRRPCS
RSAKSQGTNEKHILNHSDASPFSWYNNRKSLQPIFLTLDGNHEEGNSNPRQDLESNNGMRMD
DVHAGCNDVSPHFYHAKSSLPPACSPKQVGKQEYSPLPVSTSVHSDPDVDDSEQRYRWCDEA
TNSSNDQTLQGQNKQEPIEELRCSSLIDDQSACSHLCNDVENHEKSCTHGAVPSWSNASSVVT
LAAAIDKGTTMETFNNSNCFIHDGLKGMDTHRSSQREAALMKFRLKRKDRCFEKKVRYQNR

KRLAEQRPRVKGQFIRQMQHGTPLDNGDSHKI
>GbARR35
MTMAGEFPAGLKILIVDDDRTCLLVLERMLRKFSYQVTKCQQAREALALLRQDKNRFDIVLC
DLHMPDINGFELLQIIEVEMDLPVVMMSSDDGKGVVMKGIVHGACDYLVKPVRMEAIGLIWQ
HVVRKKKKRFSGEITRSLPLQPADNAVPPMDKRSLKYRKRTSEDEDVAEDGESSEGKKPRMV
WTQELHDLFVATVNELGRGNAVPKKILERMQAMNVTFLTRANIASHLQKYRMHLQKEGPVP
SSDSRDVNAYIDHRNLQFQPSPTTPYQLPMQNLITERANENVLSIAPSHVDGGSNIFNSNIASES
SCSLPTQVTLYNLYRANLLYQNDFPPTSNGVPISNDNEFPPNDGVANSNDESFYCNIVDGTELS
NPFSAVAVDGTELSNPLSAVEELIHEPSFLVGQYDQQAFFRGPI
>GbARR36
MMEMRNTNGVVAEELGTLEGDDLKVDKIAQNVKDCHTGVVRTPAVLQIQQQQPQSASGCW
ERFLHQASIKVLLVENDDSTRHVVAALLRNCRYEVIEAASGLQAWKILEDLTNHIDLVLTEVF
MPCFSVIFLLSKIMSHKTHKNVPVIMMSSHDSIGIVFKCLSKGAVDFLVKPIRKNELKNLWQHV
WRRCHSSSGSGSGSESGTRTQKSEKSESVENSDNSGSNDEEDNENIGLNVGDGSDDGSGTQSS
WTKQAAEIESPRPGSPQDRVAECPDSTCAQVIHSNAEASGNKGVPKTAARGCQELDEQLDNV
AMGKGLDIRMAGSVDLQREHPVEVPIKIIGAKQINLLEISFNKLNEPIDKRQLDLNTKSLSGELN
SEAAHQTGITSKTNDLKKESTEYEASNRISKISDGNDKATDDSKEVLPSAELGFKRLRGAEDTE
AMLRDERNVLRRSNSSAFSRYNTASNANKVSVVNTGSSSARDSKLELTRKGSVCDVQSPLVN
DLPNQCSNVGSNNIDMASTTDNAFAKPAVLKNKSASSSAFRSGHPSSAFQPMKNDLLNAARK
PILDKADGITTKAGLKQPRLTHQELDMQDCPQHQQVTDHDTLSLKKMAADAPHCGSSNVLG
GPVPVEGNAGNYSVNGSNSGSNHASNGPHGSSTIADTVGTNIESDNGIAGKSGSGGSGDASGA
GSGSGSGSKVDQSKSASREAALTKFRQKRKD
>GbARR37
MEEKRRSNCGEGFSVLVVDHDTTSLMLLASMLQLFSYQVTTTEVGSVALSLIEEAKDRFKLV
MADVNMDDVNSLSFLRKLLKMNIPFILMSNEKGHKAAEKAVAYGASLHLEKPISKHDLKYL
WQYAYGRPAKRAKHLHDNELRTYDPNKNVGERDEQKKMLNIMPPTERKPRLLWNEELHQK
FIAAITALGYANARPKSILTMMDEPNLKQRQVASHLQKHKEHMKRLMSKASPSELASNANAS
TSIYGKPGFLYMPQGYYSVLSQLGGHTHKYLNDSVAGTSQFSYLAQHSTAGRFSSEYHNEETL
QFPNILESMHEKQNIIPDPSPSGNLVYENHNWEPWTEAARGVAEAEIGVGNAAGGVTWNQQS
PELAQLLKHLEEEGDACGDCANQPNPDAVDQFCEWLKEAIQGNDNNP
>GbARR38
MAISYESNMNNVYAGVTVLLVDGDSTCLIILSKMLRSFGYKVVTTKQATDALCIIRDQQHKID
LVLVEACLHDMDKYELLETIRNISSIPIIVMSTDYDRNSVLGSLFKGATLHLEKPITMDDIKNLW
QFTLIKGQEINVPIVEAKSCIKEVPSMESALGVVVDGRRNLRDEKRKRPLEVENDKEGDNCDQ
GSSTLKKPKLIWTNELHNRFLQAIDMLGSEAYPKKILQVMNVTGLRKENVSSHLQKHRLLLKR
QQEAIQNTISSTVSQAASHHALSEFSPRNGFHRFTDTAQTTSVAEQHGFINDLVQDNLNG
>GbARR39
MICTTNDLSEWKDFPKGLKVLLLDEDSNSAAELKSKLEAMDYIVYTFCNENEALSAVSSRPES
FHVAIVEINTNKKNGSFKFLETAKNLPTIMTSNIHCISTMMKCIALGAVEFLKKPLSEEKLRNIW
QHVVRKAFNSVGTDLSESVKPVKESLVSMLYLQLENGEPKNKDLDKTQDASVIHENDPEPST
GSDKYPAPSTPQLEQGGRLSADGDCQDQANCSIEKESSEQDGESKSVETTSDNTIAEVTIPVGQ
PLGPRDTMVTEEADLVDGTKGESTSYSQTENGVNSKNSQAVAVKPSTVSGIHSSCLNKANRK
KSKVDWTPALHKKFVQAVDQLGIDQAIPSRILELMKVEGLTRHNVASHLQKYRMHRKHILPK
EDNRRWPQRDQTQRSCYPHKPIIAFPPHYSNHDVPVGPLYPMWGAPSSIQMWGSQGYPLWQP

TESWHWKPYPGAHADAWGCPVMPPPHGYSSAFTQVSSYQNASVFHCSGTMDNRSGMPQNS
VEHQPAEEVIDKVVKEAISKPWLPLPLGLKPPSTDTVLADLSKQGISTFPPHINGPNSSCHGTT
>GbARR40
MGDFVVMSCEEVKEVKGNSDTEMESGEFEGIKKKKRKQQQESCVYGEVVKWERWMALRVL
LIEADDSTRQIIAALLKKCNYKVSAVPDGLKAWEMLKGKPHNYDLILAEVDLPSISGFALLTLI
MEHELCKSIPVIMMSSQDSVSTVYKCMLRGAADYLVKPVRRNELRNLWQHAWRRQSSIIDGN
FPGDESIGRKQVEATSENDAARQTDKGSDTQSSCIKPDMEAESADMETIQEFSHLIKGKSQPSG
SQMHEAHDGLNQNLLMHETKTSVNDCKDPNLTTAYKGVELECQRTNMNISAEAGNALADSP
REAIDFMGTFNRNFNFSSINSTCKFDSSPPLDLSLRRCNNNDFKDNVTRERPILWHPNSSAFSRY
SNRLSLPQHSTLTSISNKKKESGSNSETILSNIVSEHDAATPSPMVTSQRNMIPSTAGATDQSRH
TEVATSCMEQRECPSSVNQQEPTFGVNPFHHSSLEMNSSRQFYDRLASGTNQMDQMLDSVED
KGHISPTVNQSGTSSFCNGSISQLNGIAYGSSSASYSNVDQVIVRASTERKNDDNIPAPAGNSHR
SIQREAALTKFRLKRKDRCYEKKVRYESRKKLAEQRPRVKGQFVRQVQVDHPTQTIHPMASY
NIEKCMD
>GbARR41
MVSTRSVNSPAMKITILVVDDDSTSLAIVSAMLREFRYEVTSVKTPAAALSVLRSNPSIDLVVT
DLHMPGMNGIELQKRINKEFKLPVIIMSSDDDENVMLESLAGGAVFFIVKPVDPIGLKNVWQY
AVAAKKGKSLLIEDMDRESSSSSPADGKLSLGGNTKSVSSVNNEKNDPKNGSKRKGASGKGR
DKDDNDDESKSPPPKKTKKPKIVWTNTLHNQFLEALRQIGLERAVPKKILERMNSTGLTRENV
ASHLQKYRIFLKRIAERGCFASKAVIDRFLKSNFAAGHPLLLKTAQEYSRLEHMERLRVLASYP
GLHESLMGHSSTRNVPLFYGHPGASSSNAAQQPLGYGQSRLLSNQTNQPLFPGSGNMLNNPN
LNNHLGYGNGIGIGIGSSSSVNGGGGFSSGLLNGGNSSLTYPNQVQARPDFYNAGQSSSSPFRF
GSAGFHSSGSTLGNGLFGSSSSSYPSLNSSSSGSIVVNGLFGSSSRSHPSLNSSSSGATLGNGLFG
SSSSSYPSLNNSSYPTLNPGYTNNAANSNRGMRFYEHLLNGSAPPLTVDYGSMNQTRPTGDYG
SMNRTHNENINVPTMRTTPLDSLDFMREFPIPPVDNSTLIQGLGTGNTRLTEINSDQLLNNVPN
LDSEPRGGDGLLQDLVLESKKLANEEKAGEQSVENSDYCLPSLFPEIYPTLDELLNCDFPDPLS
DEDNHPWSEEAIGQVQSELEELINNPNPAAGDGRSNKSNPITNKQQTGGEQVTVTHPLSNVLQ
VMTAPAPAPAPAPAPAPAPAPAPALAVYNSANNANNGAPVGANSSSYEDDEDFLDSLLNFND
EFE
>GbARR42
MVMAASETKFHVLAVDDTLIDRKLIEKLLKTSSYQGSFLCSQTGFSFSTKHGFVLILIWVVFAV
TAVDSGTKALEFLGLNNNDHHDVEVNLIITDYCMPGMTGYDLLRKIKESSSFKDIPVVTMSSD
NIPSRINRCLEDGAEEFFLKPVQLSDVNKLRPHLMKRRTQTNVNKRKAMDEIVSPDRTRARYN
ELEVK
>GbARR43
MTVEQIGSEGKDQFPIGMRVLAVDDDPTCLLLLGTLLRRCQYHVTTTSQAKTALKMLRENKN
KFDLVISDVHMPDMDGFKLLEHVGLEMDLPVIMLSANGDTKLVMKGITHGACDYLLKPVRIE
ELQNIWQHVVRRKKKDRCNSGSKDKPHPDSGEAVGIGNVDNNGKLNKKRKDQNEDEDDER
DENGHDNEDPSAQKKPRVVWSVELHRKFVAAVNQLGIDKAVPKKILELMNVEKLTRENVAS
HLQKFRLYLKRISCVANQQANMAAALGTADSAYLRMGSLNGLGNFHTLAGSDQLHNAAFRS
FPPSGVLGRLNTPAGLGIRGLPSPGTIQLGHVQNSGNPTNDLSKLQSFVPGNHNTNILQGMPMS
LELDRLQHNKSVGHIGELPTTDSTTVFPGSGNLVDARITGFSNNPLLGVTSNSLMLEGSSQQAT
SHTSVSAIGFQNGNALSDFTSIAPASNQLQDSKAGSQGQASPINCNAGQIIRSAPQEWNAPRKD
APYQSHALINSSIPINSAMIQLGQCLDRNNSIFHRTTDLDLVGPLNFVDPLSIKHGEGDNSIMEPS

VIEKEGYLMFQPRPHGSHVPDNIGSLEDLASAMMKQEGGCNGYSLRTSI
>GbARR44
MKNSSGGKGSMSTASSITTWKADVVPVPDQFPAGLRVLVVDDDPTCLIILEKMLRNCSYDVT
KCNRAETALLKLRENRNGFDIVISDVHMPDMDGFKLLEHIGLEMDLPVIMMSADDGKDVVM
KGVTHGACDYLIKPVRIEALKNIWQHVVRKRKNEWKDLEQSGSVEEGDRQPKQSEDADYSSS
VNEGNWKSSKKRKDDEDETEDRDDTSTLKKPRVVWSVELHQQFVAAVNQLGIDKAVPKKIL
ELMNVPGLTRENVASHLQKYRLYLRRLSGVSPHSCNPNNSFMNPQDETFGPLSSVHGLDLQTL
TATGQLPAQCLATLQAAVLGRSTAKSSIPMPLVNQRNIFSFENPKLRFGEGQQHVNNNNKQVN
LLHGIPTTMESKQLTSLRHTSQSIGNVNMQVAPHGAQSSQNNSSLIEMGQPLSRVQILNDSTVP
LSVGQPIVPNGIAANVSTRNGIPENIRAPGYNLVSQTSSILNFPMNHASELPVDSFSLRSTPGMS
DHTSKGAFHEDFNSEIKGSGGFLPTYDVFNDYQYKSQNWELQNAGMILEDSQHSNSLQGNLD
LAQSVLVQQGFPSGQINGQNRSVPIVSKAMFSAGDSTEPGNLLNVNHHLNTIRAENTVRVKSG
SVADGNPSNLFADHFGQEDLMIALLKQQQGIAPAENEFDFDGYSLDNIPV
>GbARR45
MNPSNGNGSMSTASSSGAWDGRDQFPAGLRVLVVDDDPTCLMIFEKMLKACLYQVTKCNKA
ETALSVLREHKNEFDIVISDVHMPDMDGFELLKHITSEMDLPVILISADARKQVVMEGVTYGA
CDYLIKPVRIETLKNIWQHVVRKKARNTV
>GbARR46
MATMHRVVQSSVSTSDATTTSYDGLTSCKAADIVISDQFPAGLRVLVVDDDITCLKILEQMLH
RCRYHVTTCPQAKVALNLLRERKGCFDVILSDVYMPDMDGYKLLEHVGLEMDLPVIMMSAD
GSTRAVMKGIRHGACDYLIKPIREEELKNIWQHVVRKKWNENKELEHSGSLDDTDQHKQRHD
DAEYASSVNDATETSLKPLKKRSNSKEEDDGEIDNDDPSTSKKPRVVWSVELHQQFVSAVNQ
LGIDKAVPKRILELMNVPGLTRENVASHLQKFRLYLKRISGVAQQGGIANPLCGPVEANVKIGS
LGSFNIQALAASGQIPPQTLAAVHAELLGRSAGNLVVATDQPALLQATPQGPKCIQVDQGVAF
VQHSVKSESSSSKHFSQSFAPVEDVASGFRSWPSNNIGTAGPSNSGGLSSQNGNMLIDLLQQQQ
QLQKPQQRSTVSELRRSINVQPSCHVVPSQSSASFRAGNSPVSVTQNGSYSRTAVIDYSLLSSQS
NCPSLNIGQVSDVNLQTTGVLSGYIPPASVSPSVSSCSVNADNCASQQVQTSSMTFKASRHLPG
FVHSTSNIPDPYGSTKSGDLLNQEPFNNLGYINKGTCLPAKFAVDEFQSHLSSSSHGKVFSENIG
TRVKQEPSMEFGDNAKVGIPMLQQFPPNDLMSVFTE
>GbARR47
MVLAESGFSSPRNDAFPAGLRVLVVDDDPTWLKILEKMLKKCSYEVTTCCLAREALNLLRER
KDGYDIVISDVNMPDMDGFKLLEHVGLEMDLPVIMMSVDGETSRVMKGVQHGACDYLLKPI
RMKELRNIWQHVFRKKIHEVRDIESLEGFESIQMTRSGYDLFDDGHFLSGDDTTSGRKRKDAD
NKHDDRELSDPSSTKKARVVWTVDLHQKFVKAVNQIGFDKVGPKKILDLMNVPWLTRENVA
SHLQKYRLYLSRLQKESDIKNSFIGMKHSDLPSKDSTASFGPHKAMNMIPDDVPNGTYSFSAS
NSLAQNVDLKGQGDLKGITSAPVAEPKGALSIDIHDSHEAKSTQMSFDHSLGSVDSAVSFASF
NSTTPLQYPWTEIPEIQFKQECEPLHLENGFSQLPLPGPSMIENEANRSRIEVKPLLEESRSNFVE
HLGPVGAEDLFPIQSKSQSLNNQVFDLISATKSSMKTQDVGLNYLADSEFALRNLNASGVGVP
LATLSEDLQICWLQGDCYPMNYGLQDLECSTYDNPALMAETPFHLYDVLRFDHEHLFDPTEY
YAIDQGLFA
>GbARR48
MESKELNLNKELKAGGAGNGFIDRSKVRILLCDNDTKSCEEVFSLLLKCSYQVTTVRSARQVI
DALNAEGPDIDIILTEVDLPMTKGMKLLKYIMRNNELRHIPVIMMSAQDEVSIVVKCLRLGAA
DYLVKPLRTNELLNLWTHMWRRRRELGLLEKNILNCDFDLVASDPSDANTNSTTLFSDDTDE

RSRKSSNPEMGVSTHQEDESAAATVEPPQSDSPECRPDVPGISDRRTGQFSSVPKKSELKIGESS
AFFTYVKSSAVKTSSTQVATPNHESAAQNKISEENLPQPGEQLVSDTRVHENGETWENNSQGD
DFRSSSSVPDSLSLERSSTPASMEFSQQRDFKEDKFSPALVPPRNETQHDVSGLPTQSAYLHYM
PGVLNQVMMPSSTQLFQNNLHDIHNHTSSPVVPQYNHLQQCLPHPHVSGMASFPYYPVNMC
MQPGQMPTGHSWPSFGNSSSNEVQPSKVDRREAALIKFRQKRKERCFDKKIRYVNRKRLAER
RPRVRGQFVRKNGITVDLNGQPASADYDEDEEEEQASRDSSPEDDTSGC

>GbARR49

MVCTANDLSTWKDFPKGLKILLLDGDTNSANELKSKLEAMDYIVYAFHDENEAISAVSSRPES
FHVAIVEVSTDNNNGSFKFLETAKDLPTIMTSNIHCISTMMKCIARGAVEFLKKPLSEDKLRNI
WQHVVHKAFNTGGNDLTESLKPVKESVVSMLQQQPENLKPKNEDSETTEDASMIHENDSEPP
AGNDKYPAPSTPQLEQGGRLLDDRDCQDHTHCSIEKGSGDQDGETESVETTGGNTTATGGQP
RGPSETNVKEEDDSVDGTMGESPQNRVDSKGSDVVAEKPSSCPDKANRKKSKVDWTPELHK
KFVQAVDQLGIDQAIPSRILELMEVEGLTRHNVASHLQKYRMQRRHILPKDDDRRWLQRPQT
QRICNYPHKPIMAFPPYNHVPVGPVYPMWRAPSHPPTIQMWGTQGYPPWQPTESWHWKPYP
GVQADAWGCPVMPPPQGYFSTFPQNASGFQPCNVDKRNGMPQTLVEHHPEEEVIDKMVKEAI
NKPWLPLPLGLKPPSTDSVLAELYRQGISTVPPHINASDR

>GbARR50

MGEVVVTSEEVEEVKIDSETEEQENGEVEGNMRRRRRKKKKKKKKPGCSASGAVVNWERF
LPMPALRVLLVEADDSTRQIISALLRKCSYRVAAVPDGLKAWEMLKGRPHNVDLILTEVDLPS
ISGFALLTLIMEHDICKSIPVIMMSSQDSVSTVYKCMLRGAADYLVKPIRRNELRNLWQHVWR
RKSSIVGGNSPQDESIGQKKVETTSENNAASNHSIGCLDGVRKNKEQTEKGSDAQSSCTKPEM
EAESAQKENMQEFSHLIKVNSPPIESQKHEAHGSFNQNLLMHEMETEVVDSCEDAYTTLYKG
VELENQRRDTRVLVEAGDALVDSPREAIDFTGTFNKNCTSSSINSAKKFDSSLFLDLSLRRSNP
NVFENHVTQERPTLWHPSSSAFTRYTSRVSQPLHSTLMSFSDQKKDSGTNSEKMLTNVMSEN
NSDTPSPTLTSQRNTNPLTIGATVELKQTEVATPCTQHRLFPVPVPVKGIRLNNPCNGYNTIIPP
MFCARSSSSMAPSPSTANQQEPAFHLNLFRHSSFEANSSGQLYDRLASNTNQSTSQLLHKLDQ
KLDSIEDRGHISPTTDQSASSSFCNGSLSQLNGVAYGSTGASNGNVDQAAVTRASTESKNDDSF
PSPSGKPCRSIQREAALMKFRLKRKDRCFEKKVRYESRKKLAEQRPRVKGQFVRQVQADPMH
IETEHHYGNSSDG

>GbARR51

MGNMATQTQFHVLAIDDSLIDRKLIERLLKTSSYQVTAVDSGSKALEFLGLNGENEDEERNSS
VESVSAADEDHQVGVNLIITDYCMPGMTGYDLLRKIKQSSSFKDIPVVIMSSENIPSRINRCLED
GAEEFFLKPVKLSDVNKLRPHLMKGITTKNEMQSNTNKRKGSEEIQSPDRTRPRYNELEVV

>GbARR52

MGMVQMNNNSPVTSGLVELNTHICDENTNIMDGVTGEGQGLSDEDESRINEDVENRNKGKK
AVVQRRGPLVCWERFLPLRSLKVLLVENDDSTRHVVCALLRNCFFFSILAVTAVSNGLLAWKI
LEDLTNHIDLVLSEVVMPCLSGIGLLCKIMNHKTRKNIPVIMMSSHDSMNIVLKCLSKGAVDFL
VKPIRKNELKNLWQHVWRKCHSSSGSGSGSGIQTQKSTKSKSGDSDNNTGNNKEDDIGSVGL
NAQDRSDNGSGTQSSWTKRAIEVDSSQPISARDQVLHSRSEVLGNIWVPVTITRECDGRDDEL
DNAVKGKDLEIGVPKITALQLENPSEKVNTNVAGGNQEKLSELNPSKDDEKLEKAQLELTGEK
LGVDLVNQAADVIGVISKNTDAQIESAVFDIPDGLPQVSDTKGKVIYKTKEMPSLELSLKRLID
VGDSGTSAPERNVLRHSDLSAFSRYNSGATANQAPVGNVGSCSPLDNSLEAANTDSMKNFHS
NSNNMPPNQQSNSNNNDMGSTTNNAFSKSAVLNDKPASKTSVPSSALQPVQKGHATAMQPP
AEDKADAAIGKKILTKAKGTDQQVRVQHHHHHYYHYHHHVHKMPQNQKLDNQDDLCQCG

SSNMSSAPHVEANAGNHSSNGSAPESNHGSNGQNGNITALNSRGLNLESENGLLGKGGTVGGI
GFGGSNGAHQNRFAQREAALNKFRQKRKERCFEKKVRYQSRKKLAEQRPRIRGQFVRQVPEN
KNKDTNC
>GbARR53
MARNGGVAWMRTTEKIDGYDLSTSDTEEVHVLAVDDSLVDRKVIERLLRISSCKVTAVDSGR
RALQYLGLDEEIQKKETNGFDGLKVDLIITDYCMPGMTGYELLKKVKESSAFREIPVVIMSSEN
VIARIDRCLEEGAEDFIVKPVKLSDVKRIIDYTTTELREGEQSEGARAKRRGINKRKQREGDDD
LSSSPPSTLSSSTSSSPSSSIQSATAPSSPSTLDSPTRRLKMTSSE
>GbARR54
MEEIFLENLIDPQIDDDHTLKELTVFAFNTQVLSLLYLCAVLHNCNYKVKSASSAVEVIEILRTN
KHGIDIVLVDVDTADLNAFKVMETIGLETNLPVIMVTADSNLENITKGLAHGAVDCIIKPFEME
QIKNTIQSHVASNKTRGQYLNPLPVSNARMAVKDACCSSKLKKKRLAWSRELDAKFVKAVQI
LEKGSENVHPKRILDVMNEPGVTRAHISSHLQKYRLALKKRKADTNKQGLEVEPTNYCLKKR
EVGNFNADRRRLAVQSFNGVMSPSSSSPDPKKQSQSLVYSCIDGHGPEFQTPDFYYNNHCLET
NIQPHNVESEHVSGTTTLSPYFNGVDSKASILSSAAVYPFPNAVFLEPEANIAFHSHFDAISVPNP
LYGFTIDCSTDVQYPSSTVFARNSETHAVQNGTTISTSDTSSYHYVKPENEVSETYNSQIECSLF
EEESFDWYSLLDDQYLSSLT
>GbARR55
MTLQEKRGGSNGEDGGKDRFPIGMHVLAVDDDPVCLKVLENLLRKCQYHVSTTNQATTALK
MLRENRNRYDLVITSVNMPDMDVFKLLELVGLEMDLPVIMLSTHGDTELVMKGITHGACDY
LLKPVRIEELKNIWQHVVRKNKPDFKDHVNALNQDNGHEDEDPSIQEKPRVVWSDEVHRKFV
SAVNELGLDKAVPKKVLDLMNVEGLKRETVASHLQKYRLYIGHSIVAALGSKDPSYFRMGPL
DGFGYFHTLTGRISSASLPSYQPGGMFGRLNSSAALSLHGISSSVIQPGHCQTLNNPINGSGKIQ
PAVVPANQKKNGTLFHGIPTSVDLNQPTQNKPTNGCGEFNRVNDPNFQDARMAVGGSCNTLH
VSSGNPLLLQSNTQQTKHSGAFGNQASLSNSLLDLYSFKAKSANLGDSKGDISNVGLNNYIILN
IDYATKQQWGDSRYDNNGNTNHSFCRADSLVLFLFVCFVLFCCVYKICQKTYDAWC
>GbARR56
MTMEEKLGGSNGEDGGRDRFPIGMRVLAVDDDPICLKVLENLLRKCQYHVTTTNQAITALKM
LRENRNRYDLVISDVNMPDMDGFKLLELVGLEMDLPVIMLSAHSDTKLVMKGITHGACDYLL
KPVRIEELKNIWQHVVRKKKPDSKDQINASNQDKSRGGTGETGPTSSSSDQKVSKKRKDQSED
EDEEGDDNGHEDPSTQKKSRVVWSVELHRKFVSAVNQLGLEKAVPKKILDLMNVDGLTREN
VASHLQKYRLYLKRLSSVATQQANMVAALGSKDPSYLRMGSLDGFGDFRTLTGPGRISSASLS
SYQPGGLFGRLNSSAALSLRGISSGVIQSGHSQTLNNPINGFGKIQPAVVPANQNQNGTLFQGIP
TSINQLSQNKPTNHFGEFNRCNDPNAFGVATNFQDARVMVGGSSNTLPVSSGNPLLLQSNTQQ
TQHSGGAFGNQPSSLGVTSLNQEPFDMNVRGSSNFLDHGRCSENWQSAVQLSSFPSNALSASE
TFSHEQLPSNNLQESISWTSSHLSGSPLDLSSSMANPANLEDSRGNISQVGLNNNVIQNIDYTTK
QQWGDSRHDYNGNMNNSFSRGDSLVPASGPMMDQSNVISDKMNDVSLFSQFSGDSTYVVPH
LDGEKSAFDTKSRSNDDFLFETKPQNGFNQNSFEPLENIMMSMIKSDQNNETPLMDGEFGFDA
YSLGSCI
>GbARR57
MRVERVISDENDSFPVGMRVLAVDDDPTCLLLLETLLTKCKYNVTTTSQAITALKMLRENKN
KFDLVISDVHMPDMDGFKLLELVGLEMDLPVIMLSANGDTKLVMKGITHGACDYLLKPVRIE
ELQNIWQHVIRRKKFDRKDRDGSGSQDKPYIDSSEAAGLGNVDQNGKFNKKRKDQNEDEDE
GRDENGHDNEDLSTQKKPRVVWSVELHRKFVAAVNQLGVEKAVPKKILELMNVENLTRENV

ASHLQKFRLYLKRISCVANQQANMAAALGSVDSTHLRMGTMNGPGNFHTLAGPDQIHNAAF
RSFQHSGVLRRLNTPAGVGISGLPSSGLVQLSPVQNLGNTSTYQRKLQSFVIPGNHNANILQGM
PMSLEFDQLQNNRAASHVGQFPSVDNTTVLPVSSISGSLIDAGCSNSPLLGVTSNSLLLEGSSQL
PTLHASNLRDSVSGFPNRSTLSDFTSIAPASNQLHGSKADIQDQAFPISCNAGKIISSAPQDWNA
PKKDAPYQSNVPSCSINSLRAVNGDMAQLGQCLDQNNPIFNRNVGFDFVDPLSIKCSEGENLA
MEPSVIEKEGYLMVQPSRQGIYIPDNEGENFAMEPSVIEKEGYLIVQPRRQGSYIPGNLGSLEDL
ASAMTKQDHDKGKPTDGDFGYNDYSLRTCI

>GbARR58

MCHEQKEARHGVERDGQGSGSIGENGSRIVERTLNVNNGSLEAIEVHDVSEIPQQQPRGSMIR
WERFLPFRTIKVLLVENEDLTRHLVSALLQNCSYEVVAVANGLQAWKLLEDPTNHIDIVLTEE
DMPVLSGSDLLCMIMNHKMLKNIPVIMMSSHDCINLVFKCLSEGAVDFLVKPIRKNELKNLW
QHVWRRCHGSSGSGSVSESGTLSKKSIKSKVNDEPENYAANTDEHDDDSDVLVGCNGSENGS
GTQSSWTKRAAEGESSQLMSSLNRFPDAPNSTCAQVVHVKHEKCGSPWTCVTRRKECQEQHE
QLLDATEGKDLEVRVESNHEWQCGNQCENSPTHLAEAASKPFDRGWFEHQDENITGNDRTPD
IITTLQQAECRASDAPGGPSDVPQLKDGACHGSEEKLSFELTLKRWQGASDGRNAANDGHNV
LRHSDSSAFSKYSIASSAKQALTGNVGSCSPLDHSSVTKKTEIMCTFPSHSNGILLNQSSVGSNN
KNDMTTTAKFVGPKPKALVDKSGSISTFKCLHSSSFQPMHGCCICSSQEVSPETVGHDTSLKIM
AITDKQCRSSSSVNGSASGSKYGSNGHNGSETGLRAEHAVTEDSNGTASGRRGGSGADEDRV
AQRAAALTKFRQKRKERCFEKKVRYQSRKKLAEQRPRVKGQFVRRTVSDWGGKDSSSYDFT
SEDRYSDSLR

>GbARR59

MELMKSENIVQDRQPQPQEEEEIMQQEEEEEQRFHVLAVDDSVIDRKLLEKLLKASSYQVTCV
ESGEKALEYLGLLHHSSPASSSSQHHHQGHKVNLIMTDFSMPGTSGYDLLKRIKGSSWKDVPV
VVMSSENVPSRISMCLEGGAEEFMLKPLQLSDLHKIQAHLLKSLPHS

>GbARR60

MFYQIMDGDYGGSCGGCSEDMTVEFGYEPHVLAVDDNLIDRTLVEKLLKNSSCKVTTAENGL
RALEFLGLGSDERNSLEGTVSKVNLVITDYCMPGMTGYELLKKIKESSVLKEVPVVIMSSENIP
TRINQCLEEGAQMFMLKPLKQSDVKQLKRHLMKYRS

>GbARR61

MELMKSSDNIIQDRTQQQHKQEPEMQAQEGQQEQGHHFHVLAVDDSVIDRKLLEKLLKVSSY
QVTCVDSGDKALEYLGLLNNLDSDSTASSSSPSFSSSSSSSSSNSSSCSQSSQREGLKVNLIMTD
FCMPGMSGYDLLKRLKGSSWKDVPVVVMSSENVPSRISMCLEGGAEEFMLKPLQLSDLEKIE
AYLLKSLHLSCTNIDKDDDDNNANHNSDNNNDKVVNNDSNIDKGNNSTSMNNNFSKRKALS
SEDTESRRPKIKGLAVAI

>GbARR62

MTMEEKMGGSNREDEALDRFPVGMRVLAVDDDPICLKVLGTLLLKCQYQVTTTNQAISALK
MLRENRNRYDLVISDVNMPDMDGFKLLELVGLEMDLPVIMLSAHSDTKLVMKGITHGACDY
LLKPVRIEELKNIWQHVVRRKKPDSKDRVNALNQDKASGGIGEAVQTSTSSSDQKFNKKRKD
QNEDEEDDGEDNEHENDDPSTQKKPRVVWSVELHRKFVAAVNHLGLDKAVPKKILDLMNVE
GLTRENVASHLQKYRLYLKRLSSVATQQANMVAALGGKDPSYLRMGSLDGFGDFRTLTGSG
RLSTASLSSYQPSGMFGRLNSSAALNLRGISSGVIQQGHPQTLSNSINGLGKIQPVGLPANQNQ
NGTLFQGIPTSIELNQLLQNKSANHFGELNPVNNPNVSGVATNFPDARMTVGSSSSSLSTPSGSP
LLLQANTQQAQRSGAFGNQSSLDVASLNQESFDMGVHGFSNFADNGGCHENWQNTVQLSSF
PSNSLSTCEAFSHEQLHTNNLQESISWRSSHLNDNPIDLSASMANAGLEDSRGDMQCQISPSNN

VIHNIDYAAKQQWGENSSFAGADSLVAGAPYVQHLEAGKLAFNTKLRSNEDILFEQRKPQNE
FSQNNFETLDVISSPMIKPEQYNETGMMEGELGYDAFPLGSCI
>GbARR63
MVLTAFLQRILDTNKLVQGAACSALAILEEEAAEKLAPIKLENLGTARLVVDFDRWWTGEES
VNVLFESTCQVTTVKSARQVIDALNAEGPNIDIILTKVDLPMTKGMKLLKYIMRNNELRRIPVI
MMSAQDEVSIVVKCLRLGAADYLVKPLRTNELLNLWTHMWRRRRE
>GbARR64
MRVERIINETNDEFPIGIRVLAVDDDPTCLLLLETLLRRCKYNVTTTNQAITALKLLRQNKFDL
VICDVHMPDMDGFKLLELVGLEMDLPVIMLSANGDTNVVMKGITHGACDYLLKPVRIEELNN
IWQHVVRRKRFERKDWYNSDSQDKLRADSGEAVGMGSIGNNGKLKKKRKDQDEDEERDEN
GHHNNEDPSIRKKPRIVWSAELHSKFVAAVNQLGMKNATPKKILELMNVEKLTTQNVGSHLQ
AFIRTFSANSYDDPKKFRLYLKRISCAANQQVNIAAAVRMRSPNGLGNFHTLAGSNQLHNAAF
RSFPPRGVLGRLNTHAGLLIRGLPSPRTIRSGHGQSSVNSGNDQSKLQSFVSGIHNANILQGLPM
SLELDQGQTNNGVSHIGELPIADSTTVFPVSSSLIDATITGFSGNPLLGVTSHSLMLEASSQQAG
NSRDIVPAIGFRNENTLSDFAPLAPASNRESNADLQCEPIPINCNAGELITSAPREWNSPYQSNV
TPCSMNSSIPVSGTMVQFGLCLDQNNSMDSDSIGPSSFIDNSAMEQSIIDKEGYLMLEPWEQGS
HIPYNIGLLEDLWELQ
>GbARR65
MTYVVGIKVIATTYTVCFFNAMDGGFDGDFPAGLKVLVVDDNRTCLLVLETMLRKLSYEVTT
CQLARHALALLREDKNRFDIVLCDLHMPEMDGLKLLEIIGLEMDLPVVMMSSDDGKGVIMKG
IIHGACDYLVKPVQMEAVRLIWQHVVRKRQRALGDFQQLRGNIHATGRTLLLKQAKNAVDQ
MPARERRILKRARENDDEDEDDEDDEGELSEEVTTAKKPRVIWTQELHDIFVIAVNQLRQRMN
NSLLLYLYHSLMEINMLITCLPEAVPKKILERMQAMNVTGLSRANIASHLQKYRLHLRKGGGQ
PLADNRDVNLNPSIGQASSFNQFNLQFQQPTATGSSCNQLPMQNLMITPQPCTINDSVVPDSTQ
CFPTELSTNDLSQPNLLFQNDVPTSNVEHLYRNVGVNVSELSNPISSLDDSSVNQLIYEPSVLVR
QYDQGDFFHGGFPRLETLSI
>GbARR66
MSGNGASSSSLNMISGEDVVNNNLSALVVDDSPLLRLLHDIHLKKYGLKVQVAENGKVAVDL
FHLGASFDLVLMDKEMPVMNGVEATKELRAMGVTSMIVGVTSKDGPGEQQAFMEAGLDYC
FEKPLTPEIISFLLEELNKHNNKN
>GbARR67
MVCTRNDLSAWKDFPKGLRVLLLDEDTNSAAEIKSKLEAMDYIVYTFCNENEALSAVSSRPES
FHVAIVEVCTNSNNGGFKFLETAKDLPTIMTSNIHCISTMMKCIALGAVEFLRKPLSEDKLRNI
WQHVVHKAFSAGGDDLSESLKPVKDYVASMLHQQLENGECKHEDLDKIEDASMIHENDHEP
SAVNNKYPAPSTPQLQQGGRLIVNGDCQEHTNCSMEKESGEPDGESKSVETTSGHTIAEVIAPV
GQHQKPRETMVKEEADSVDGAKGERTLDSHPRDRVNSEDSRAGAEKPNTVSGLHSSRPNKA
NRKKLKVDWTTELHEKFVQAVDQLGIDQAIPSRILELMKVEGLTRHNVASHLQKYRMHKRHI
LPKEDDRRWPQRDQTQRSCHPHKPIMAFPPYHSNPVPVGPVYPMWGTPHPASIQTWGSQGYY
HPWQPTESWQWKPYPGVHADAWGCPVMPIPQGNCSTFTRNGYGFHCSSTMDNRSGMPQNSI
QHQPAEEVIDEVVKEAINKPWLPLPLGLKPPSTDSVLAELSRQGISTIPPRNQRL
>GbARR68
MAQSKHGGRKLGQSNKMTALVVDDNMINRTIHHRLLENLGVENEVVSNGKEAVDIHYSGKM
FDLILMDMDMPIMNGIEATKKLREMGIRSVIAGVSSRAMEEEIREFMEAGLDDYQEKPLTMSK
LVSIIHKING

>GbARR69
MEEKMGGSNGEDSGEDQFPVGMRVLAVDDDPICLKILANLLCKCQYQVTTTNQAIIALKMLR
GNKNRYDLVISDVNMPDMDGFKLLELVGLEMDLPVIMLSAHSDTKLVMKGITHGACDYLLK
PVRIEELKNIWQHVVRRKKPDSKDELAAPNHDKSRGGTGEAGQTWVACSSDQKVNKKRKDQ
SEDEEEETEDNGHENEDSSSQKKPRVVWSVDLHRKFVAAVNQLGLGDQAVPKKILDLMNVE
GLTRENVASHLQKYRLYLRRLSSVATQQANMVVALGSRGPSHLRTGSLDGFGDFRSFTGPRR
FSSASLPSYQSRGTFGRLNSSAALTLSGISSGVIQPGNSVNGLGKIQPVVLPANQNQNEALFQGI
TASIELNQLSQTKSTNHFGEYNCVNERNVFRISSSFPDARVVVGSSSNSLSTASGNALVLHGST
QEVQCSAAFENLPSFGMTSLNRESNDLSVRGSFNCLEHGRCSENWQGTVQLSNFPPSNEQLPS
NNLQESIPWRNSSPSNSRIALSSMASSAVLGDSRADMLCKAGLNNSYSHVDSRVSASGSMMD
QNNAVSSNTNDVSLFSHLNGEAPFAVRHSEGDKSSSDTDLRSNDNFLFLQSKPQNGFSQNNFE
SLEDIMSPVFKLEQNNETAFMDGGFGFDVAYPLGSCM
>GbARR70
MNVDGDGDKGLRELNHRLYDGSKRTTNGVVAEEHVMLEDVKVNKIAQNVKDGHVVAVQA
PAMRQITQQQPQNAMCYWQRFLHLTTVTVLLVENDDSTRHVVTALLRNCCYDVVEAANVLQ
AWKILEDLTNHIDLILAEVGMPSLSGLVLLSKIMSHKTRKNVPVIMMSSQDSMNLVFKCLSKG
AVDFLVKPIRKNELKNLWQHVWRRCHSSSGSGSESGTQTQKSVRSKSVEKSDNNSGSNDEEN
NGSIGLNIGDGSDDGSGTQNSWTKQAVEVDSPRLVSPSDQVAECPDSTCAQVVHSNAELSGN
KWVPVAAAKGCQEQDEQLDNVAVGKDLDIGMPRNLDLQLECPVEVPIRTVGAKQINLLDMS
SSKFSEQIEKRQLDLNSESPSNKQKSEAANQTGTTSKTTDLKKEIAENEVSNRLSKIPDVNDKTI
NDSKELPSVELGLKRLRGVKDAGTVVRDERNVLRRSDSSAFSRYNTASNANKVPVVNIGSSSA
LDSNLELTRKGSVCNNESHLVNELPNQSSNVGSNNIDMGSTTNNAFAKAAVDKNKSAASSTV
RSLHPSSIFQPMKNDLLSATQKVVFDKADDVSTTAGLAQARGIHHELQMQHPSNHYDQHHHL
THGMQQQQPPEHDDLSLKKLAADAPHCGSSNVWGGLVEGNAANYSVNGSASGSNHGSNGP
NGSSNAVNTVGTNMESDNGIAGKSGSGDVSGSGSGSGSGSKADQSKSAHREAALTKFRQKRK
ERCFQKKVRYQSRKRLAEQRPRIRGQFVRQTVNNDPASNGNSCDE
>GbARR71
MEVAGEVLASESQLHVLAVDDSHVDRKVIEKLLKISSCKVTTVESGARALQFLGLDEEKGSLG
FNGLKVNLIMTDYSMPGMTGYELLKKIKRSSAFREIPVVIMSSENILTRIDSCLEEGAEEFLVKP
VKLSDVKRVMNDCIMRGETEGNNRRRVDKRKFEDDNHASSSSSSSSSIPSSPSSPLPSSPSTAAT
SSPLHSLKRAKLRDHN
>GbARR72
MNSSSGKSYMSTASSSVLWKAGGDVVADQFPAGLRVLLVDDDPTCLMILEKMLKACQYNVT
KCNRAETALSMLRENRNGFDIVISDVHMPDMDGFKLLEHIGLEMDLPVIMMSADDGKNVVM
KGVTHGACDYLIKPVRIEALKNIWQHVVRKRKNEWKDFEQSGSVEEGDRQPKQSEDADYSSS
ANEGNWKSSKRRKDDEDENDERDDTSTLKKPRVVWSVELHQQFVAAVNQLGIEKAVPKKIL
ELMNVPGLTRENVASHLQKYRLYLRRLSGVSQHPSNLNNSFMNPQDATFGPLDLQALAATGQ
LPAQSLATLQAAGLGRSTAKSGIPMPLIDQRNIFSFENPKLRFGEAQQQHVSNNKQTNLLHGIP
TTMEPKQLAGLHHTAQSMGNMNMPVLSHGSQSSQNNPLLMQMAQPQARGQMLNDSTVGLA
PRLLSPRGQSMLSNGMATNVSTRNGVPENIRAPSYNPVSQTSLMLNFPMNHTSELPGNSFPLVS
TPGISSLAPKVAFQEDVNSEIKGSVGFMPSYDIFNDLNQHKPQNWELQNVGMMFNSSQHSNSL
QGNLDHTQSILVQQGFLSGQNRNSTVISKAMFSAGDSTGHVNAQNSNQHLNSLLDNTVRVKS
ERVADTAPANLYPDHFGQEDLMSALLKQQDIIAPAENNDFDFDGYSIDNIPV
>GbARR73

MNVSSVKGSMSISSSTSTWKAGDTISDQFPAGLRVLVVDDDPTCLMILEKMLIACLYKVTKCN
RAETALSKLRENKNGYDIVLSDVHMPDMDGFKLLEHIGLEMDLPVIMMSADDGKQVVMKGV
THGACDYLIKPVRIEALKNLWQHVVRKRKNEWKEFEQSGSVEEGDRQPKQSDDADYSSSANE
GNWKGSKRRKDEEEETDERDDTSTLKKPRVVWSVELHQQFVAAVNQLGIDKAVPKKILELM
NVPGLTRENVASHLQKYRLYLRRLSGVSQHQSNLSTIISAQDPTFGSLSSLSGLDLQTLAATGQ
LPAQSLARLQAAGLGRATAKSAVPITLVDQRNIFSFENPKLRFGEGQQQHMTNKQQVNLLHGI
PTTMEPKQLVSLRHTAQSVGNMNMQVPPHGPQSSQNNPLLMQMGQQQQQQKSRGQILVDST
INHAPRLSSMGQPILSNGMATNVSSRSGIPENIRAPGYSQTPSMLNFPMNHASELPGNCFPLGST
PGVSNLTSKGAFQEDVNSEIKGSGGFMPSYDVFNDLNQHKPQSWELQNVGIAFDSSQHSNSLQ
GNLDLTQSALGQQGFSSGQMNGHNRSAAVASKAMFSTGDVKELRSAQNVNQHLNNLLVDN
TIRIKSERVCDTSPANIFPDHFGQDDLMSALLKQQESVASSENEFDFDGYSMNNIPV
>GbARR74
MLGSGGGSGSCSKDMAVELGDEPHVLVVDDSLIDRKLVERLLKNSSCKVTTAENALRAVEYL
RLGNDTLEGTVSEVNMIITDYCMPGMTGYELLKKIKESSVLKEVPVIIMSSENIPTRISQCLEEG
AKMFMLKPLKLSDVKQLKWHLMKCRN
>GbARR75
MLGSGGGSGSCSKDMAVELGDEPHVLVVDDSLIDRKLVERLLKNSSCKVTTAENALRALDYL
GLGNDTLEGTVSEVNMIITDYCMPGMTGYELLKKIKESSVLKEVPVVIMSSENIPTRISQCLEEG
AKMFMLKPLKQSDVKQLKWHLMKCRN
>GbARR76
MGMAIDRDDTQFHVLAVDDSLIDRKLIERLLKTSSFQVTAVDSGSKALEFLGLNNNDADDDEE
DEQRDSNTESVSSDEDQDHQDLGLGVNLIITDYCMPGMTGYDLLKKIKKSSSFKDIPVVIMSSE
NIPSRISRCLEDGAEEFLLKPVQLSDVNKLRPHLVKGRSKDIDSSHKTTSTRLITSGN
G.hirsutum
>GhARR1
MKNSIGGKGSMSTASSITTWKAGDVVSVPDQFPAGLRVLVVDDDPTCLIILEKMLRNCSYDVT
KCNRAETALLKLRENRNGFDIIISDVHMPDMDGFKLLEHIGLEMDLPVIMMSADDGKDVVMK
GVTHGACDYLIKPVRIEALKNIWQHVVRKRKNEWKDLEQSGSVEEGDRQPKQSEDADYSSSV
NEGNWKSSKKRKDDEDETDDRDDTSTLKKPRVVWSIELHQQFVAAVNQLGIDKAVPKKILEL
MNVPGLTRENVASHLQKYRLYLRRLSGVSPHSCNPNNSFMNPQDETFGPLSSVHGFDLQTLTA
TGQLPAQCLATLQAAVLGRSTAKSSIPMPLVNQRNIFSFENPKLRFGEGQQQHVNNNNKQVNL
LHGIPTTMESKQLTSLRHTSQSIGNLNMQVAPHGAQSRQNNSSLIEMGQPLSRVQILNDSTVPL
SVGQPIVPNGIAANVSTRNGIPENIRAPGYNLVSQTSSILNFPMNHASELPVDTFSLRSTPGMSD
HTSKGAFHEDFNSEIKGSGGFLPSYDVFNDYQYKSQNWELQNAGMILDDSQHSNSLQGNLDL
TQSVLVQQGFPSGQINGQNRSVPIVSKAMFSAGDSTEPGNLLNVNHHLNTIRADNTVRVKSES
VADGNPSNLFTDHFGQEDLMIALLKQQQGIAPVENEFDFDGYSLDNIPV*
>GhARR2
MNSSNGNGSMSTASSSGAWNGRDQFPAGLRVLVVDDDQTCLMVFEKMLKTCLYQVTKCNK
AETALSVLREHKNEFDIVISDVHMPDMDGFELLKHINSEMDLPVIRNNLLFIILFWIHRVWIKLL
NFDPNLKN*
>GhARR3
MVCTANDLSTWKDFPKGLKILLLDGDTNSANELKSKLEAMDYIVYAFHDENEAILAVSSRPES
FHVAIVEVSTDNNNGSFKFLETAKDLPTIMTSNIHCISTMMKCIALGAVEFLKKPLSEDKLRNI
WQHVVHKAFNTGGNDLTESLKPVKESVVSMLQQQPENLKPKNEDSEKTEDASMIHENDSEPP

AGNDKYPAPSTPQLEQGGRLLDDRDCQDHTHCSIEKESGEQDGETESVETTSGNTTATGGQPQ
GPSETNVKEEDDSVDGTMGESPQNRVDSKGSDVVAEKPSSCPDKANRKKLKVYWTPELHKK
FVQAVDQLGIDQAIPSRILELMKVEGLTRHNVASHLQKYRMQRRHMLPKEDDRRWLQRPQM
QRICNYPHKPIMAFPPYHHVPVGPVYPMWGAPSHPPTIQMWGTQGYPPWQPTESWHWKPYP
GVHADAWGCPVMLPPQGYFSTFPQNASGFQPCNVDKRNGMPQTLVEHHLEEEVIDKMVKEA
INKPWLPLPLGLKPPSTDSVLAELYRQGISTVPPHINASGR*
>GhARR4
MESKELNLNKELKAGGAGNGFIDRSKVRILLCDNDTKSCEEVFSLLLKCSYQVTTVRSARQVI
DALNAEGPDIDIILTEVDLPMTKGMKLLKYIMRNNELRRIPVIMMSAQDEVSIVVKCLRLGAA
DYLVKPLRTNELLNLWTHMWRRRRELGLSEKNILNCDFDLVASDPSDANTNSTTLFSDDTDE
RSRKSSNPEMGISTHQEDESAAATVEPPQSDSPECRPDVPGISDRRTGQFSSVPKKSELKIGESS
AFFTYVKSSAVKTSSTQVATPNHESAAENKIGEEHLPQPGEQVVSDTRVHENGETWENNSQG
DEFRSSSSVPDSLSLERSSTPASMEFSQQRDFKEDKFSPALVPPSNETQHDVSGLPTQSPYLHYM
PGVLNQVMMPSSTQLFQNNLHDIHNHTSSPLVPQYNHLQQCLPHPHVSGMASFPYYPVNMC
MQPGQMPTGHSWPSFGNSSSNEVQPSKVDRREAALIKFRQKRKERCFDKKIRYVNRKRLAER
RPRVRGQFVRKNGATVDLNGQPASADYDEDEEEEQASRDSSPEDDTSGC*
>GhARR5
MATMHRVVQSSVSTSDATTTSYGGLTSCKAADIVISDQFPAGLRVLVVDDDITCLKILEQMLH
RCRYHVTTCPQAKVALNLLRERKGCFDVILSDVYMPDMDGYKLLEHVGLEMDLPVIMMSAD
GSTRAVMKGIRHGACDYLIKPIREEELKNIWQHVVRKKWNENKELEHSGSLDDTDQHKRGHD
DAEYGSSANDATDTSLKPLKKRSNSKEGDDGEIDNDDPSTSKKPRVVWSVELHQQFVSAVNQ
LGIDKAVPKRILELMNVPGLTRENVASHLQKFRLYLKRISGVAQQGGIANPLCGPVEANVKIGS
LGSFNIQPLAASGQIPPQTLAALHAELLGRPAGNLVVAMDQSALLQATPHGPKCIQVDHGVAF
IQHLVKSESSSSKHFSQSFAPVEDVASGFRSWPSNNIDTAGPSNSGGLSTQNGNMLIDLLQQQQ
QLQKPQQRSTVSELRRSINVQPSCHVVPSQSSASFQAGNSPVSVTQNGSYSRTAVIDYNFLSSQ
SNCPSLNIGQVSDVNLQTTGVLSGYIPPASVSPSVSSCSVYADNCASQQVQTSSITFKASRRLPG
FVHSTSNIPDPYGSTKSGDLLNQEPFNNLGYINKGTCLPAKFAVDEFQSHLSSSSHGKVFSENIG
TRVKQEPSMEFGDNAKVGIPMLQQFPPNDLMSVFTE*
>GhARR6
MVLAESGFSSPRNDAFPAGLRVLVVDDDPTWLKILEKMLKKCSYEVTTSCLAREALNLLRER
KDGYDIVISDVNMPDMDGFKLLEHVGLEMDLPVIMMSVDGETSRVMKGVQHGACDYLLKPI
RMKELRNIWQHVFRKKIHEVRDIESLEGFESIQMTRSGYDLFDDGHFLSGDDTTSGRKRKDAD
NKHDDRELSDPSSTKKARVVWTVDLHQKFVKAVNQIGFDKVGPKKILDLMNVPWLTRENVA
SHLQKYRLYLSRLQKESDIKNSFIGMKHSDLPSKDSTASFGPHKAMNMIPDDVPNSTYSFSASN
SQAQNVDLKGQGDLKGITSAPMAEPKGALSVDIRDSHEAKSTQMSFDHSLGSVDSAVSFASFN
STTPLQYPWTEIPEIQFKQECESLHLENGFSQLPLPGPSMIENEANRSRIEVKPLLDDCRSNFVEH
LGPVGAEDLFPIQSKSQSLNNQVFDLISATKSSMKTQDVGLNYLADSEFALRNLNASGVGVPL
ATLSEDLQICWLQGDCYPMNYGLQDLECSTYDNPALMAETPFHLYDVLRFDHEHLFDPAEYY
AIDQGLFA*
>GhARR7
MARNGGVAWMRTTEKIDGYDLSTSDTEEVHVLAVDDSLVDRKVIERLLRISSCKVTAVDSGR
RALQYLGLDEEIQKKETNGFDGLKVDLIITDYCMPGMTGYELLKKVKESSAFREIPVVIMSSEN
VIARIDRCLEEGAEDFIVKPVKLSDVKRIIDYTTTELREGEQREGARARRRGINKRKQREGDDD
LSSSPPSTLSSSASSSPSSSIQSATAPLSPSTLDSPTRRLKMTSSE*

>GhARR8
MEEIFLENLIEPQIDDDHTLKELTVFAFNTQVLSLQYLCAVLHNCNYKVKSASSAVEAIEILRTN
KHGIDIVLVDVDTADLNAFKVMETIGLETNLPVIMVTADSNLENITKGLAHGAVDCIIKPFEME
QIKNTIQSHVASNKTRGQNLNPLPGSNARMAVKDACCSSKRKKKRLVWSRELDAKFVKAVQI
LEKGSENVHPKRILDVMNEPGLSRAHISSHLQKYRLALKKRKADTNKQGLEVEPTNYCLKKR
EIGNFNADRRRLAIQSFNGVMSPSSSSPDQKKQSQSLVYSCINGHGPEFQTPDFYYNNHRLETN
IQPHNVESEHVSGTTTLSPYFNGVDSEPIILSSAAVYPSPNAVFLEPEANIAFHSHFDVISVPNPL
YGFTIDCSTEVQYPSSTVLGRNSETYAVQNGTTISPSDTSSYHYVEPENEVSGTYNSQIECSLFE
EESIDWYSLLDDQ*
>GhARR9
MTLQEKRGGSNGEDGGKDRFPIGMHVLAVDDDPVCLKVLENLLRKCQYHVSTTNQATTALK
MLRENRNRYDLVITGVNMPDMDAFKLLELVGLEMDLPVIMLSTHGDTELVMKGITHGACDY
LLKPVRIEELKNIWQHVVRKNKPDFKDHINALNQDNGHEDEDRSIQEKPRVVWSDEVHRKFV
SAVNELGLDKAVPKKVLDLMNVEGLKRETVASHLQKYRLYIGHSIVATLGSKDPSYLRMGPM
GGFGYFHTLTGPGRISSASLPSYQPGGMFGRLNTSATLSLHGISSSVIQPGHCQTSNNPINGSGKI
QPAVVPANQKKMELCFKGSQHR*
>GhARR10
MTMEEKLGGSNGEDGGRGRFPIGMRVLAVDDDPICLKVLENLLRKCQYHVTTTNQAITALKM
LRENRNRYDLVISDVNMPDMDGFKLLELVGLEMDLPVIMLSAHSDTKLVMKGITHGACDYLL
KPVRIEELKNIWQHVVRKKKPDSKDQINASNQDKSRGGTGETGPTSSSSDQKVSKKRKDQSED
EDEEGDDNGHEDPSTQKKPRVVWSVELHRKFVSAVNQLGLEKAVPKKILDLMNVEGLTREN
VASHLQKYRLYLKRLSSVATQQANMVAALGSKDPSYLRMGSLDGFGDFRTLTGPGRISSASLS
SYQPGGLFGRLNSSAALSLRGISSGVIQSGHSQTLSNPINGFGKIQPAVVPANQNQNGTLFQGIP
TSINQLSQNKPTNHFGEFNRGNDPNAFGVATNFQDARVTVGGSSNTLPVSSGNPLLLQSNTQQ
TQHSGAFGNQSSSLGVTSLNQEPFDMNVRGSSNFLDHGRCSENWQSAVQLSSFPSNALSTSEA
FSHEQLPNNNLQESISWTSSHLSSSPLDLSSSMANPANLEDSRGNISQVSLNNNVIQNIDYTTKQ
QWGDSRHDYNGNMNNSFSRGDSLVPASGPMMDQSNLISDKMNDVSLFSQFSGDSTYVVPHL
DGEKSAFDTKPRSNEDFLFETKPQNGFNQNSFEPLENIMMSMIKSDQNNETPLMDGEFGFDAY
SLGSCI*
>GhARR11
MRVERVISDENDSFPVGMRVLAVDDDPTCLLLLETLLTKCKYNVTTTSQAITALKMLRENKN
KFDLVISDVHMPDMDGFKLLELVGLEMDLPVIMLSANGDTKLVMKGITHGACDYLLKPVRIE
ELQNIWQHVIRRKKFDRKDRNGSGSQDKPYIDSSEAAGLGNVDQNGKFNKKRKDQNEDEDEE
RDENGHDNEDLSTQKKPRVVWSVELHRKFVAAVNQLGIEKAVPKKILELMDVENLTRENVAS
HLQKFRLYLKRISCVANQQANMAAALGSVDSTHLRMGTMNGLGNFHTLAGPDQIHNAAFRS
FQHSGVLRRLNTPSGVGISGLPSSGLVQLSPVQNLGNTSTNQSKLQSFVIPGNHNANILQGMPM
SLEFDQLQNNRAASHVGQFPSVDNTTVLPVSSISASLIDAGCSSSPLLGVTSNSLLLEGSSQLPT
LHARNLRDSVSGFPNRSTLSDFSSIAPASNQLHGSKADIQDQASPISCNAGKIISSAPQDWNAPK
KDAPYQSNVPSCSINSLRAVNGDMAWLGQCLDQNNPIFNRNVGFDFVDPLSIKFSEGENLAME
PSVIEKEGYLMVQPSRQGSYIPDNEDENFAMEPSVIEKEGYLIVQPRRQGSYIPGNLGSLEDLAS
AITKQDHDKGKPTDGDFGYNDYSLRKCI*
>GhARR12
MCHEQKEARHGVVRDGQGSGSIGENGSRIVERTLNVNNGSLEAIEVHDVSEIPQRQPRGSMIR
WERFLPFRTIKVLLVENEDLTRHLVSALLQNCSYEVVAVANGLQAWKLLEDPTNHIDIVLTEE

DMPVLSGSDLLCMIMNHKMLKNVPVIMMSSHDCINLVFKCLSKGAVDFLVKPIRKNELRNLW
QHVWRRCHSSSGSVSESGTLSKKSIKLKVNDEPENYAANSDEHDDDSDVPVGCNGSENGSGT
QSSWTKRAAEGESSQPMSSLNRFPGAPKSTCAQVVHVKHEKRRSPWTCVTQRKECQEQHEQL
LDATEGKDLEVRVESNHEWQCGNQCKNSPTHLAEAASKTFDRGWFEHQDENITGKDRTPDII
ATLQQAECRASDAPGGPSDVPQLKDGACHGSEEKLSFELTLKRWQGASDGRNAANDGHNVL
RHSDSSAFSKYSTASSAKQALTGNVGSCSPLDHSSVTKKTEIMCTFPSHSNGILLNQSSVGSNN
KNDMTTTAKFVGPKPKALVDKSGSISTFKCLHSSSFQPMHGCCICSSQEVSPETVGHDTSLKIM
AITDKQCRSSSSVNGSASGSNYGSNGHNGSETGLRAEHAVMEDGNGTASGRSGGSGANEDRV
AQRAAALTKFRQKRKERCFEKKVRYQSRKKLAEQRPRVKGQFVRRTISDWEGGKDNSSYDFT
SEDRYSDSLR*

>GhARR13

MELMKSENIVQDRQPQPQEEEEIMQQEEEEEQRFHVLAVDDSVIDRKLLEKLLKASSYQVTCV
ESGEKALEYLGLLHHSSPASSSSQHHHQGHKVNLIMTDFSMPGTSGYDLLKRIKGSSWKDVPV
VVMSSENVPSRISM*

>GhARR14

MDGDYGGSCGGCSEDMTVEFGYEPHVLAVDDNLIDRTLVEKLLKNSSCKVTTAENGLRALEF
LGLGSDQRNSLEATVSKVNLVITDYCMPGMTGYELLKKIKESSVLKEVPVVIMSSENIPTRINQ
CLEEGAQMFMLKPLKQSDVKQLKRHLMKYRS*

>GhARR15

MSSHDSMNIVFKCLSKGAVDFLVKPIRKNELKNLWQHVWRKCHSSSCSGSGSGIQTQKSTKS
KSGGSDNNTGNNKEDDIGSVGLNAQERSDNGSGTQSSWTKRAIEVDSSQPISARDQVMHSRSE
VLGNSWVPVTITRECDSRDDELDNAVKGKDLEIGVPKITALQLENPSEKVNTNAAGGNQEKLS
ELNPSKDDEKLEKAQLELTGEKPGVDLVNRAADVIGVISKNTDAQIESAVFDVPDGLPKVSDT
KGKVIYKTKEMPSLVLSLKRLIDVGDSGTSAHERNVLRHSDLSAFSRYNSGSTANQAPIGNVG
SCSPLDNSLEATNTDSMKNFHSNSNNMPPNQQSNGSSNNNDMGSTTNNAFSKSAVLNDKPAS
KTSSPSSAFQPVQKGHATAMQSPAEDKADATIGKKILAKAKGTDQQVQVQHHHHYYHYHH
HVHKMPQNQKLDNQDDLCQCGSSNMSSAPHVEANAGNHSSNGSAPESNHGSNGQNGNITAL
NSRGLNLESENGLLGKGGTVGGIGFGGSNGADQNRFSQREAALNKFRQKRKERCFEKKVRYQ
SRKKLAEQRPRIRGQFVRQVPENKNKDTNC*

>GhARR16

MGNMATQTQFHVLAVDDSLIDRKLIERLLKTSSYQVTAVDSGSKALEFLGLNGENEDEERNSS
VESVSAADEDHQVGVNLIITDYCMPGMTGYDLLRKIKQSSSFKDIPVVIMSSENIPSRINRCLED
GAEEFFLKPVKLSDVNKLRPHLMKGITTKTEMQSNTNKRKGSEEIQSPDRTRPRYNELEVV*

>GhARR17

MGEVVVTSEEVEEVKIDSETEEQENGEVEGNMRRRRRKKKKKKKKKPGCSASGAVVNWER
FLPMPALRVLLVEADDSTRQIISALLRKCSYRVAAVPDGLKAWEMLKGRPHNVDLILTEVDLP
SISGFALLTLIMEHDICKSIPVIMMSSQDSVSTVYKCMLRGAADYLVKPIRRNELRNLWQHVW
RRKSSIVGGNSPQDESIGQKKVETTSENNAASNHSIGCLDGVRKNKEQTEKGSDAQSSCTKPE
MEAESAQKENMQEFSHLIKVNSPPIESQKHEAHGSFNQNLLMHEMETEVVDSCKDAYTTLYK
GVELENQRRDTRVLVEAGDALVDSPREAIDFMGTFNKNCTSSSINSAKKFDSSLFLDLSLRRSN
PNVFENHVTQERPTLWHPSSSAFTRYTSRVSQPLHSTLMSFSDQKKDSGTNSEKMLTNVMSEN
NSDTPSPTLTSQRNTNPLTIGATVELKQTEVATPCTQHRLFPVPVPVKGIRLNNPCNGYNTIIPP
MFCARSSSSMAPSPSTANQQEPAFHLNLFRHSSFEANSSGQLYDRLASNTNQSTSQLLHKLDQ
KLDSIEDRGHISPTTDQSASSSFCNGSLSQLNGVAYGSTGASNGNVDQAAVTRASTESKNDDSF

PSPSGKPCRSIQREAALMKFRLKRKDRCFEKKVRYESRKKLAEQRPRVKGQFVRQVQADPMH
IETEHHYGNSSDG*
>GhARR18
MELMKSSDNIIQDRTQQQHKQEPEMQAQEGQQEQGHHFHVLAVDDSVIDRKLLEKLLKVSSY
QVTCVDSGDKALEYLGLLNNLDSDSTASSSSSSSSSSSSSSSSSNSSSCSQSSQREGLKVNLIMT
DFCMPGMSGYDLLKRVKGSSWKDVPVVVMSSENVPSRISMCLEGGAEEFMLKPLQLSDLEKI
QVYLLKSLHISCTNIDKDDDDNNTDDNSDNNNDKVVNNDSNIDKGLNMPLLLLVIMEWELKV
VSGGTGKDEDRGITSAKMEKGIVVLVEEKKRASGVAKVGEGRLDDDNVIGEVKKEVEKRVG
EAEEEK*
>GhARR19
MTMEEKMGGSNREDEAMDRFPVGMRVLAVDDDPICLKVLGTLLLKCQYQVTTTNQAISALK
MLRENRNRYDLVISDVNMPDMDGFKLLELVGLEMDLPVIMLSAHSDTKLVMKGITHGACDY
LLKPVRIEELKNIWQHVVRRKKPDSKDRVNALNQDKASGGIGEAVQTSTSCSDQKFNKKRKD
QNEDEEDDGEDNEHENDDPSTQKKPRVVWSVELHRKFVAAVNHLGLDKAVPKKILDLMNVE
GLTRENVASHLQKYRLYLKRLSSVATQQANMVAALGGKDPSYLRMGSLDGFGDFRTLTGSG
RLSSASLSSYQPSGMFGRLNSSAALNLRGISSGVIQQGHPQTLSNSINGRGKIQPVGLPANQNQ
NGTLFQGIPTSIELNQLSQNKSANHFGELNPVNNPNVFGVATNFPDARMTVGSSSSSLSTPSGSP
LLLQANTQQAQHSGAIGNQSSLGVASLNQESFDMGVHGFSNFVDNGRCHENWQNTVQLSSFP
SNSLSTCEAFSHEQLHTNNLQESISWRSSHLSDNPIDLSASMANAGLEDSRGDMQCQISPSNNV
IHNIDYAAKQQWGENSSFAGADSLVSGAPYVQHLEAGKLAFNTKLRSNEDFLFEQRKPQNEFS
QNNFETLDIISSSMIKPEQYNETGMMEGELGYDAFPLGSCI*
>GhARR20
MRVERIINDTNDEFPIGIRVLAVDDDPTCLLLLETLLRRCKYNVTTTNQAITALKLLRQNKFDL
VISDVHMPDMDGFKLLELVGLEMDLPVIMLSANGDTNVVMKGITHGACDYLLKPVRIEELNN
IWQHVVRRKRFERKDRYNSDRQDKLRADSGEAVGMGSIGNNGKLKKKRKDQDEDEERDEN
GHHNNEDPSFRKKSRVVWSAELHSKFVAAVNQLGMKNATPKKILELMNVQKLTTQNVGSHL
QAFIRTFSAHSYDEPKKFRLYLKRISCAANQEVNIAATVRMRSPNGLGNFHTLAGSNQLHNAA
FRSFPPRGVLRRLNTHAGLLIRDLPSPGTIRSGHGQSSVNSGNDQCKLQSFVSGIHNANILQGLP
MSLELDQIQTNNGVSHIGELQIADSTTVFPVSSSLIDATITGFSGNPLLSVTSNSLMLEASSQQAG
NSRDIVPAIGFRNGNTLSDLAPIAPASNRESNADLQREPIPINCNARELITSAPQEWNSPYQSNVT
PCSMNSSIPVSGTMVQFGLCLDQNNSMDSDSIGPSSFIDNSAMEQSIIDKEGYLMLEIWGQGSHI
PYNIGLLEDLWELQ*
>GhARR21
MMYVVGIKVNATTYTVCFFNAMDGGFDGDFPAGLKVLVVDDNRTCLLVLETMLRKLSYEVT
TCQLARHALALLREDKNRFDIVLCDLHMPELDGLKLLEIIGLEMDLPVVMMSSDDGKGVIMK
GIIHGACDYLVKPVQMEAVRLIWQHVVRKRQRALEDFQQLRGNIHATGRTLLLKQAKNAVD
QMPARERRTLKRARENEDEDDEGELSEEVTTAKKPRVIWTQELHDIFVIAVNQLRQRMNNSLL
PYLYHSLMEINMLITCLPEAVPKKILERMQAMNVTGLSRANIASHLQKYRLHLRKGGGQPLAD
NRDVNLNPSIGQASSFNQFNLQFQQPTAIGSSCNQLPMQNLMITPQPCTINDSVVPDSTQCFPTE
LSTNDLSQPNLLFQNDVPTSNVEHLYRNVGVNVSELSNPISSVDDSSVDQLIYEPSFLVRQYDP
EDFFHGGFPSSLLFQADRLDSQFS*
>GhARR22
MSGNGASSSSLNMISGEDVVNNNLTALVVDDSPLLRLLHDIHLKKYGLKVQVAENGKVAVDL
FHLGASFDLVLMDKEMPVMNGVEATKELRAMGVTSMIVGVTSKDGPGEQQAFMEAGLDYC

FEKPLTPEIISILLEELNKHNKKD*
>GhARR23
MVCTRNDLSAWKDFPKGLRVLLLDEDTNSAAEIKSKLEAMDYIVYTFCNENEALAAVSSRPE
SFHVAIVEVCTNSNNGGFKFLETAKDLPTIMTSNIHCISTMMKCIALGAVEFLRKPLSEDKLRNI
WQHVVHKAFSVGGDDLSESLKPVKDYVASMLHQQLENGECKHEDLDKIEDASMIHEKDHEP
SAVNNKYPAPSTPQLQQGGRLIVNGDCQEHTNCSMEKESGEPDGESKSVETTSGHTIAEVIAL
VGQHQKPRETMVKEEADSIDGAKGERTVDSHPQDRVHSEDSCAGADKPNTVSGLHSSRPNKA
NRKKLKVDWTTELHKKFVQAVDQLGIDQAIPSRILELMKVEGLTRQNVASHLQKYRMHKRHI
LPKEDDPRWLRRDQTQRSCHPHKPIMAFPPYHSNPVPVGPVYPMWGAPHPASIQTWGSHGYY
HPWQPTESWQWKPYPGVHADAWGCPVMPIPQGNCSTFTQNGYGFHCSSTMDNRSGMPQNSI
QHQPAEEVIDEVVKEAINKPWLPLPLGLKPPSTDSVLAELSRQGISTIPPRNQRL*
>GhARR24
MAQSKNGGRKLGQSNKMTALVVDDNMINRTIHHRLLDNLGIENEVVSNGKEAVDIHYSGKM
FDLVLMDMDMPIMNGIEATKKLREMGIRSVIAGVSSRAMEEEIQEFMEAGLDDYQEKPLTMP
KLVSIIHKING*
>GhARR25
MEEKMGGSNGEDSGEDQFPVGMRVLAVDDDPICLKILANLLCKCQYQVTTTNQAIIALKMLR
ENKNRYDLVISDVNMPDMDGFKLLELVGLEMDLPVIMLSAHSDTKLVMKGITHGACDYLLKP
VRIEELKNIWQHVVRRKKPDSKDELAAPNHDKARGGTGEAGQTWVACSSDQKVNKKRKDQS
EDEEEETEDNGRENEDSSCQKKPRVVWSVDLHRKFVAAVNQLGLGDQAVPKKILDLMNVEG
LTRENVASHLQKYRLYLRRLSSVATQQANMVVALGSRGPSHLRTGSLDGFGDFRSFTGPGRFS
SASLPSYQSRGTFGRLNSSAALTLSGISSGVIQPGNSVNGLRKIQPVVFPANQNQNGVLFQGITA
SIELNQLSQTKSTNHFGKYNCVDERNVFRISSSFPDARVVVGSSSNSLSTASGNALVLQGNTQQ
VQCSAAFENLPSFGMTSLNRESNDLSVRGSSNCLEHGRCSENWQGTVQLSNFPPSNEQLPSNN
LQESIPWRNSSPSNGCIALSSMASSAFLGDSRADVLCKAGLNNSYSHVDSRVSASGSMMDQSN
AVSSNTNDVSLFSHLNGEAPFAVWLSEGDKSSFDTDLRSNDNFLFLQSKPQNGFSQNNFESLE
DIMSPVFKLEQNNETAFMDGGFGFDVAYPLGSCM*
>GhARR26
MNVDGDGDKGLREQNHRLYDGSKRTTNGVVAEEHVMLEDVKVNKIAQNVKDGHVGAVQA
PAMRQIAQQQSQNAMCYWQRFLHLTTVTVLLVENDDSTRHVVTALLRNCCYDVVEAANVL
QAWKILEDLTNHIDLILAEVGMPSLSGLVLLSKIMSHKTRKNVPVIMMSSQDSMNLVFKCLSK
GAVDFLVKPIRKNELKNLWQHVWRRCHSSSGSGSESGTQTQKSVRSKSVEKSDNNSGSNDEE
NNGSIGLNIGDGSDDGSGTQNSWTKQAIEVDSPRLVSPPDQVAECPDSTCAQVVHSNAELSGK
KWVPVAAAKGCQEQDEQLDNVAVGKDLDIGMPRNLDLQLECPVEVPMRTVGAKQINLLDM
SSSKFSEQIGKRQLDLNSESPSNKQNSEAANQTGITSKTTDLKKEIAENEDSNRLSKIPDGNDKT
INDSKEAPSVELGLKRIRGVKDAGTVVRDERNVLRRSGSSAFSRYNTASNANKVPVVNIGSSS
ALDSNLELTRKGSVCDNQSHLVNYLPNQSSNVGSNNIDMGSTTNNAFAKAAVDKNKSAASST
VRSLHPSSIFQPMKNDLLSATQKVVFDKADDVTTTAELAQARGIHHELQMLHPSNHYDQHHH
LTHGMQKQQQRQPPEHDDLSLKKLAADAPLCGSSNVLGGLVEGNAANYSVNGSASGSNHGS
NGPNGSSNAVNTVGTNMESDNGIAGKSGSGDASGSGSGSGSGSGSGSKADQSKSAHREAALT
KFRQKRKERCFQKKVRYQSRKRLAEQRPRIRGQFVRQTVNNNDPASNVNSCDK*
>GhARR27
MEVAGEVLASESQLHVLAVDDSHVDRKVIEKLLKISSCKVTTVESGARALQFLGLDEEKGSLG
FNGLKVNLIITDYSMPGMTGYELLKKIKRSSTFREIPVVIMSSENILTRIDSCLEEGAEEFLVKPV

KLADVKRVMNDCIMRGETEGNKRRRVDKRKFEDDNHASSSSSSSSSSIPSSPSSPLPSSPSTAAT
SSPLHSLKRAKLRDHN*
>GhARR28
MNSSSGKSYMSTASSSVIWKAGGDVVADQFPAGLRVLVVDDDPTCLMILEKMLKACQYNVT
KCNRAETALSMLRENRNGFDIVISDVHMPDMDGFKLLEHIGLEMDLPVIMMSADDGKNVVM
KGVTHGACDYLIKPVRIEALKNIWQHVVRKRKNEWKDFEQSGSVEEGDRQPKQSEDADYSSS
ANEGNWKSSKRRKDDEDENDERDDTSTLKKPRVVWSVELHQQFVAAVNQLGIEKAVPKKIL
ELMNVPGLTRENVASHLQKYRLYLRRLSGVSQHPSNLNNSFMNPQDATFGPLDLQALAATGQ
LPAQSLATLQTAGLGRSTAKSGIPMPLVDQRNIFSFENPKLRFGEAQQHVNNNKQMNLLHGIP
TTMEPKQLAGLHHTAQSMGNMNMPVLSHGSQSSQNNPLLMQMAQPQARGQMLNDSTVGLA
PRLLSPMGQPMLSNGMATNVSTRNGVPENIRAPSYNPVSQTSSMLNFPMNHTSELPGNSFPLV
STPGISSLAPKVAFQEDVNSEIKGSVGFMPSYDIFNDLNQHKPQNWELQNVGMMFNTSQHSNS
LRGNLDHTQSVLVQQGFSTGQNRNSTVISKAMFSAGDSTGHVNAQNGNQHLNSLFVDNTVR
VKSERVADAAPANLYPDHFGQEDLMSALLKQQDVIAPAENNDFDFDGYSIDNIPV*
>GhARR29
MNVSSVKGSMSISSSTSTWKAGDTISDQFPAGLRVLVVDDDPTCLMILEKMLIACLYKVTKCN
RAETALSKLRENKNGYDIVLSDVHMPDMDGFKLLEHIGLEMDLPVIMMSADDGKKVVMKGV
THGACDYLIKPVRIEALKNIWQHVVRKRKNEWKEFEQSGSVEEGDRQPKQSDDADYSSSANE
GNWKGSKKRKDEEEETDERDDTSTLKKPRVVWSVELHQQFVAAVNQLGIDKAVPKKILELM
NVPGLTRENVASHLQKYRLYLRRLSGVSQHQSNLSTIISAQDPTFGSLSSLSGLDLQTLAATGQ
LPAQSLARLQAAGLGRATAKSGIPITLVDQRNIFSFENPKLRFGEGQQQHMTNKQQVNLLHGIP
TTMEPKQLVSLRHAAQSIGNMNMQVPPHGAQSSQNNPLLMQMGQQQQQQQQQSRGQILVDS
TINHAPRLSSSMGQPILSNGIATNVSSRNGIPENIRAPGYSQTPSMLNFPMNHASELPGNSFALG
STPGVSILTSKGAFQEDVNSEIKGSGGLMPSYDVFNDLDQHKLQSWELQNVGMAFDTSQHSN
SLQGNLDLTQSALGQQGFSSGQMNGHNRSAAVASKAMFSTGDVKELGSAQNVNQHLNNLLV
DNTIRVKSERVCDTSPANIFPDHFGQDDLMSALLKQQESVASSENEFDFDGYSMNNIPV*
>GhARR30
MSSWKRSGMLVQRKSMKLLCQFESERAHAAMLDLCHRIFPLTFLSENLKEAGFCLRLLHPEP
SLRPTTRDILQSKVLNRFQEVFAEELSSSINRDDTESELLLHFRDDTVVISFARSVDDGRDAQA
MRVKEFFADPSHFPVDQISRHKSLGVVDPQNLLEEKEAIEEKLALSDYELRLAREDVMKLKTE
LQRKHAVRLKESLFMTLRIIRSTKMVKTATWLYRFTIGEVLAVKVTDQNLDVWANSLASVPD
ALCHYYYQYLSSSSEAAEGILCIGFLKRSRQQKEITREQAVQDLELLAFSNHFRDAIIDGLFTRM
LSSGGGSGSCSKDMAVELGDETHVLVVDDSLIDRKLVERLLKNSACKVTTAENALRAVEYLR
LGNDTLEGTVSEVNMIITDYCMPGMTGYELLKKIKESSILKEVPVIIMSSENIPTRISQCLEEGAK
MFMLKPLKRSDVKQLKWHLMKCRN*
>GhARR31
MHSSGGGSGSCSKGMAMELGDEPHVLVVDDSLIDRKLVERLLKNSSCKAENALRAVNYLRL
GNDTLEGTVSEVNMIITDYCMPGMTGYELLKKIKESSVLKEVPVVIMSSENIPTRISQCLEEGA
KMFMLKPLKRSDVKQLKWHLMKCRN*
>GhARR32
MLSCGGGSGGCSKDMAVELGDEPHVLAVDDNLIDRKLVERLLKNSSCKVTTAENALRALDY
LGLGNDRLEATVSEVNMIITDYCMPGMTGYELLKKIKESSVLKEVPVVIMSSENIPTRTSQCLE
EGAKMFMLKPLKQSDVKQLKWHLMKCRN*
>GhARR33

MGMAIDRDDTQFHVLAVDDSLIDRKLIERLLKTSSFQVTAVDSGSKALEFLGLNNNDADDDEE
YEQRDSNTESVSSDEDQDHQDLGLGVNLIITDYCMPGMTGYDLLKKIKKSSSFKDIPVVIMSSE
NIPSRINRCLEDGAEEFFLKPVQLSDVNKLRPHLMKGRSKDIDSSHKTTSTRLISSGN*
>GhARR34
MGEVLMSSDEVKTESEVDRENEEFEGNKRRKIMSGGSFVSGEAVNWESFLPTMALRVLLVEA
DDSTRQIVTALLRKCNYRVSAVPDGLQAWEMLKAKPHNIDLILTEVDLPSISGFALLTLIMEHE
ICKSIPVIMMSSQDSISTVYKCMLRGAADYLVKPLRRNELKNLWQHVWRRQQLIASGNTPQDE
SVGQKKVEAISENNAASNCSSGCLVGGERNKEQTEKGSEAQSTCTKPDMEAEDANMENMQE
FLLLPSNSQEHEVQTNFNQRLLVHEKKTGAVGACKDTEILVEAGDALGDSPRKAIDFMGTFDR
NCNSSLMNSTSKVGSLTHLDFSLGRCYPNAFKNHATREKPTLWHPNSSAFTRYSSRPSQPLQS
ALTSASDQKKESGTDSEKMLPNSITEYNSDTASPTLTPQRNTNPLTTGTTGQLKQTEVAASCTQ
QVILPVLVPSPSLANQKELACCVNPFHHPSFKSNSSGQFYDRLASNANQLTNQPLQKLDQKMN
STEDRGHIYPTTDQSATSSFCNGSLSQLNGIAYGSSAASNNSNIEQVAVVRASADSKNDDVFPS
PSGNSHRSIQREAALTKFRLKRKDRCFEKKVRYESRKKLAEQRPRVKGQFVRQPQADPSHYG
NSSDG*
>GhARR35
MVEVGMEVDVEVGTAEETKKEEEEEEEENDESSEVVRWEKFLPQMVLRVLLVEADDSTRQII
AALLRKCSYRVAAVPDGLMAWETLKDRPHNIDLILTEVELPSVSGFALLTLVMEHDICKNIPVI
MMSSEDSFSMVLKCMLKGAADFLIKPVRRNELRNLWQHVWRRHMLAGGCANHSLPAKEHN
VEDTAENNAAENNQSSDYGSSTQKIKDESDTQGLSQWKCMSSNVSDTGREQQGNSVKPGQES
LQNEGQAEGISDKLFVSENSERFVEVAHCSGTCESIALKLEENSAFVDEMAHNVVGLQSDKRI
AYVMIGVGCNDELRESSTGAIDLIDSFDKQPMDTFAISSLSDGANKLEFSPQLELSLRRPCSRSA
KSQGTNEKHILNHSDASPFSWYNNRKSLQPIFLTLDGNHEEGNSNPRQDLESNNGMRMDDVH
AGCNDVSPHFYHAKSSLPPACSPKQVGKQEYSPLPVSTSVHSDPDVDDSEQRYRWCDEATNSS
NDQTLQGQNKQEPIEELRCSSLIDDQSACSHLCNDVENHEKSCTHGAVPSWSNASSVVTLAAA
IDKGTTMETFNNSNCFIHDGLKGMDTHRSSQREAALMKFRLKRKDRCFEKKVRYQNRKRLAE
QRPRVKGQFIRQMQHGTPLDNGDSHKI*
>GhARR36
MAGEFPAGLKILIVDDDRTCLLVLERMLRKFSYQVTKCQQAREALALLRQDKNRFDIVLCDL
HMPDINGFELLQIIEVEMDLPVVMMSSDDGKGVVMKGIVHGACDYLVKPVRMEAIGLIWQH
VVRKKKKRFSGEITRSLPLQPADNAVPPMDKRSLKYRKRTGEDEDVAEDGESSEGKKPRMVW
TQELHDLFVATVNELGRGNAVPKKILERMQAMNVTFLTRANIASHLQKYRMHLQKEGPVPSS
DSRDVNAYIDHRNLQFQPSPTTPYQLPMQNLITERANENVLSIAPSHVDGGSNIFNSNIASESSC
SLPTQVTLYNLYRANLLYQNDFPPTSNGVAISNDNEFPPNDGVANSNDESFYCNVVDGTELSN
PFSAVAVDGTELSNPLSAVEELIHEPSFLVGQYDQQAFFRGPI*
>GhARR37
MMEMRNTNGVVAEELRTLEGDDLKVEIAQNVKDCHTGVVRTPAVLQIQQQQPQSASGCWER
FLHQASIKVLLVENDDSTRHVVAALLRNCRYEVIEAASGLQAWKILEDLTNHIDLVLTEVFMP
CFSGIFLLSKIMSHKTHKNVPVIMMSSHDSIGIVFKCLSKGAVDFLVKPIRKNELKNLWQHVW
RRCHSSSGSGSGSESGTRTQKSEKSESVENSDNSGSNDEEDNENIGLNVGDGSDDGSGTQSSW
TKQAAEIESPRPVSPQDRVAECPDSTCAQVIHSNAEASGNKGVPKTAARGCQELDEQLDNVA
MGKGLDIRMAGSVDLQREHPVEVPIKIIGAKQINLLEMSFNKLNEPIDKRQLDLNTKSLSGELN
SEAAHQTGITSKTNDLKKESTEYEASNRISKISDGNDKTTDDSKEVLPSAELGFKRLRGAEDTE
AMLRDERNVLRRSNSSAFSRYNTASNANKVSVVNTGSSSARDSKLELTRKGSVCDVQSPLVN

DLPNQCSNVGSNNIDMASTTDNAFAKPAVLKNKSASSSAFRSGHPSSAFQPMKNDLLNAARK
PILDKADGITTKAGLKQPRLTHQELDMQDCPQHQQATDHDTLSLKKMAADAPHCGSSNVLG
GPVPVEGNAGNYSVNGSNSGSNHSSNGPHGSSTIADTVGTNIESDNGIAGKSGSGGSGDASGA
GSGSGSGSNVDQSKSARREAALTKFRQKRKDRCFRKEVRYQSRKRLAQQRPRIRGQFVRQTV
NTNDPLSEANSSDK*
>GhARR38
MEEKRRSNCGEGFSVLVVDHDTTSLMLLASMLQLFSYQVTTTEVESVALSLIEEAKDRFKLV
MADVNMDDVNSLSFLRKLLKMNIPFILMSNEKGHKAAEKAVAYGASLHLEKPISKHDLKYL
WQYAYGRPAKRAKHLHDNELRTYDPNKNVGERDEQKKMLNIMPPTERKPRLLWNEELHQK
FIAAITALGYANARPKSILTMMDEPNLTQRQVASHLQKHKEHMKRLMSKASPSELASNANAS
TSIYGKPGFLYMPQGYYSVLSQLGGHTHKYLNDSVAGTSQFSYLAQHSTAGRFSSEYHNEETL
QFPNILESMHEKQNIIPDPSPSGNLVYENHNWEPWTEAARGVAEAEIGVRNAAGGVTWNQQS
PELAQLLKHLEEEGDACGDCANQPNPDAVDQFCEWLKEAIQGNDNNP*
>GhARR39
MAISYESNMNNVYAGVTVLLVDGDSTCLIILSKMLRSFGYKVVTTKQATDALCIIRDQQHKID
LVLMEACLHDMDKYELLETIRNISSIPIIVMSTDYDRNSVLGSLFKGATLHLEKPITMDDIKNL
WQFTLIKGQEINVPIVEAKSCIKEVQSMESALGVVVDGRRNLRDEKRKRPLEVENDKEGDNCD
QGSSTLKKPKLIWTNELHNRFLQAIDMLGSEAYPKKILQVMNVTGLRKENVSSHLQKHRLLLK
RQQEAIQNTISSTVSQAASHHALSEFSPRNGFHRFTDTAQTTSVAEQHGYINDLVQDNLNG*
>GhARR40
MICTTNDLSEWKDFPKGLKVLLLDEDSNSAAELKSKLEAMDYIVYTFCNENEALSAVSSRPES
FHVAIVEINTNKKNGSFKFLETAKNLPTIMTSNIHCISTMMKCIALGAVEFLKKPLSEEKLRNIW
QHVVRKAFNSVGTDLSESVKPVKESLVSMLYLQLENGEPKNKDLDKTQDASVIHENDPEPST
GSDKYPAPSTPQLEQGGRLSADGDCQDQANCSIEKESSEQDGESKSVETTSDNTIAEVTIPVGQ
PLGPRDTMVTEEADLVDGTKGESTTYSQTENGVNSKNSQAVAVKPGTVSGIHSSCLNKANRK
KSKVDWTPALHKKFVQAVDQLGIDQAIPSRILELMKVEGLTRHNVASHLQKYRMHRKHILPK
EDNRRWPQRDQTQRSCYPHKPIIAFPPHYSNHDVPVGPLYPMWGAPSSIQMWGSQGYPLWQP
TESWHWKPYPGAHADAWGCPVMPPPHGYSSAFTQVSSYQNASVFHCSGTMDNRSGMPQNS
VEHQPAEEVIDKVVKEAISKPWLPLPLGLKPPSTDTVLADLSKQGISTFPPHINGPNSSCHGTT*
>GhARR41
MGDFVVMSCEEVKEVKGNSDTEMESGEFEGIKKKKRKQQQESCVYGEVVKWERWMALRVL
LIEADDSTRQIIAALLKKCNYKVSAVPDGLKAWEMLKGKPHNYDLILAEVDLPSISGFALLTLI
MEHELCKSIPVIMMSSQDSVSTVYKCMLRGAADYLVKPVRRNELRNLWQHAWRRQSSIIDGN
FPGDESIGRKQVEATSENDAARQTDKGSDTQSSCIKPDMEAESADMETIQEFSHLIKGKSQPSG
SQMHEAHDGLNQNLLMHETKTSVNDCKDPNLTTAYKGVELECQRTNTNISAEAGNALADSP
REAIDFMGTFNRNFNFSSINSTCKFDSSPPLDLSLRRCNNNDFKDNVTRERPILWHPNSSAFSRY
SNRLSLPQHSTLTSISNKKKESGSNSETILSNIVSEHDAATPSPMVTSQRNMIPSTAGATDQSRH
TEVATSCMEQRECPSSVNQQEPTFGVNPFHHSSLEMNSSRQFYDRLASGTNQMDQMLDSVED
KGHISPTVNQSGTSSFCNGSISQLNGIAYGSSSASYSNVDQVIVRASTERKNDDNIPAPAGNSHR
SIQREAALTKFRLKRKDRCYEKKVRYESRKKLAEQRPRVKGQFVRQVQVDHPTQTIHSMASY
NIEKCMD*
>GhARR42
MVSTRSVNSPAMKITILVVDDDSTSLAIVSAMLREFRYEVTSVKTPAAALSVLRSNPSIDLVVT
DLHMPGMNGIELQKRINKEFKLPVIIMSSDDDENVMLESLAGGAVFFIVKPVDPIGLKNVWQY

AVAAKKGKSLLIEDMDRESSSSSPADGKLSLGGNTKSVSSVNNEKNDPKNGSKRKGASGKGR
DKDDNDDESKSPPPKKPKKPKIVWTNTLHNQFLEALRQIGLERAVPKKILERMNSTGLTRENV
ASHLQKYRIFLKRIAERGCFASKAVIDRFLKSNFAAGHPLLLKTAQEYSRLEHMERLRVLASYP
GLHESLMGHSSTRNVPLFYGHPGASSSNAAQQPLGYGQSRLLSNQTNQPLFPGSGNMLNNPN
LNNHLGYGNGNGIGIGSSSSVNGGGGFSSGLLNGGNSSLTYPNQVQARPDFYNAGQSSSSPFRF
GSAGFHSSGSTLGNGLFGSSSSSYPSLNSSSSGSIVVNGLFGSSSRSHPSLNSSSSGATLGNGLFG
SSSSSYPSLNNSSYPTLNPGYTNNAANSNRGMRFYEHLLNGSAPPLTVDYGSMNQTRPTGDYG
SMNRTHNENINVPTMRTTPLDSLDFMREFPIPPVDNSTLIQGLGTGNTRLTEINSDQLLNNVPN
LDSEPRGGDGLLQDLVLESKKLANEEKAGEQSVENSDYCLPSLFPEIYPTLDELLNCDFPDPLS
DEDNHPWSEEAIGQVQSELEELINNPNPAAGDGRSNKSNPITNKQQTGGEQVTVTHPLSNVLQ
VMTAPAPAPAPAPAPAPAPAPAPALAVYNSANNANNGAPVGANSSSYEDDEDFLDSLLNFND
EFE*
>GhARR43
MVMAASETKFHVLAVDDTLIDRKLIEKLLKTSSYQVTAVDSGTKALEFLGLNNNDHHDVEVN
LIITDYCMPGMTGYDLLRKIKESSSFKDIPVVTMSSDNIPSRINRCLEDGAEEFFLKPVQLSDVN
KLRPHLMKRRTQTNVNKRKAMDEIVSPDRTRARYNELEVK*
>GhARR44
MTVEQIGSEGKDQFPIGMRVLAVDDDPTCLLLLGTLLRRCQYHVTTTSQAKTALKMLRENKN
KFDLVISDVHMPDMDGFKLLEHVGLEMDLPVIMLSANGDTKLVMKGITHGACDYLLKPVRIE
ELQNIWQHVVRRKKKDRCNSGSKEKPHPDSGEAAGIGNVDNNGKLNKKRKDQNEDEDYERD
ENGHDNEDPSAQKKPRVVWSVELHRKFVAAVNQLGIDKAVPKKILELMNVEKLTRENVASH
LQKFRLYLKRISCVANQQANMAAALGTADSAYLRMGSLNGLGNFHTLAGSDQLHNAAFRSF
PPSGVLGRLNTPAGLGIRGLPSPGTIQLGHVQNSGNPTNDLSKLQSFVPGNHNTNILQGMPMSL
ELDRLQHNKSVGHIGELPTTDSTTVFPGSGNLVDARITGFSNNPLLGVTSNSLMLEGSSQQATS
HTSVSAIGFQNGNALSDFTSIAPASNQLQDSKAGSQGQASPINCNAGQIIRSAPQEWNAPRKDA
PYQSHASINSSIPINSAMIQLGQCLDRNNSIFHKTTDLDSVGPLNFVDPLSIKHGEGDNSIMEPSV
IEKEGYLMFQPRPHGSHVPDNIGSLEDLASAMMKQEGGCNGYSLRTSI*
>GhARR45
MKNSSGGKGSMSTASSITTWKADVVPVPDQFPAGLRVLVVDDDPTCLIILEKMLRNCSYDVT
KCNRAETALLKLRENRNGFDIVISDVHMPDMDGFKLLEHIGLEMDLPVIMMSADDGKDVVM
KGVTHGACDYLIKPVRIEALKNIWQHVVRKRKNEWKDLEQSGSVEEGDRQPKQSEDADYSSS
VNEGNWKSSKKRKDDEDETEDRDDTSTLKKPRVVWSVELHQQFVAAVNQLGIDKAVPKKIL
ELMNVPGLTRENVASHLQKYRLYLRRLSGVSPHSCNPNNSFMNPQDETFGPLSSVHGLDLQTL
TATGQLPAQCLATLQAAVLGRSTAKSSIPMPLVNQRNIFSFENPKLRFGEGQQHVNNNNKQVN
LLHGIPTTMESKQLTSLRHTSQSIGNVNMQVAPHGAQSSQNNSSLIEMGQPLSRVQILNDSTVP
LSVGQPIVPNGIAANVSTRNGIPENIRAPGYNLVSQTSSILNFPMNHASELPVDSFSLRSTPGMS
DHTSKGAFHEDFNSEIKGSGGFLPTYDVFNDYQYKSQNWELQNAGMILEDSQHSNSLQGNLD
LAQSVLVQQGFPSGQINGQNRSVPIVSKAMFSAGDSTEPGNLLNVNHHLNTIRAENTVRVKSG
SVADGNPSNLFADHFGQEDLMIALLKQQQGIAPAENEFDFDGYSLDNIPV*
>GhARR46
MNPSNGNGSMSTASSSGAWDGRDQFPAGLRVLVVDDDPTCLMIFEKMLKACLYQVTKCNKA
ETALSVLREHKNEFDIVISDVHMPDMDGFELLKHITSEMDLPVILISADARKQVVMEGVTYGA
CDYLIKPVRIETLKNIWQHVVRKKARNTV*
>GhARR47

MATMHRVVQSSVSTSDATTTSYDGLTSCKAADIVISDQFPAGLRVLVVDDDITCLKILEKMLH
RCRYHVTTCPQAKVALNLLRERKGCFDVILSDVYMPDMDGYKLLEHVGLEMDLPVIMMSAD
GSTRAVMKGIRHGACDYLIKPIREEELKNIWQHVVRKKWNENKELEHSGSLDDTDQHKQRHD
DAEYASSVNDATETSLKPLKKRSNSKEEDDGEIDNDDPSTSKKPRVVWSVELHQQFVSAVNQ
LGIDKAVPKRILELMNVPGLTRENVASHLQKFRLYLKRISGVAQQGGIANPLCGPVEANVKIGS
LGSFNIQALAASGQIPPQTLAAVHAELLGRSAGNLVVATDQPALLQATPQGAKCIQVDQGVAF
VQHSVKSESSSSKHFSQSFAPVEDVASGFRSWPSNNIGTAGPSNSGGLSSQNGNMLIDLLQQQQ
QLQKPQQRSTVSELRRSINVQPSCHVVPSQSSASFRAGNSPVSVTQNGSYSRTAVIDYSLLSSQS
NCPSLNIGQVSDVNLQTTGVLSGYIPPASVSPSVSSCSVNADNCASQQVQTSSMTFKASRHLPG
FVHSTSNIPDPYGSTKSGDLLNQEPFNNLGYINKGTCLPAKFAVDEFQSHLSSSSHGKVFSENIG
TRVKQEPSMEFGDNAKVGIPMLQQFRPNDLMSVFTE*
>GhARR48
MVLAESGFSSPRNDAFPAGLRVLVVDDDPTWLKILEKMLKKCSYEVTTCCLAREALNLLRER
KDGYDIVISDVNMPDMDGFKLLEHVGLEMDLPVIMMSVDGETSRVMKGVQHGACDYLLKPI
RMKELRNIWQHVFRKKIHEVRDIESLEGFESIQMTRSGYDLFDDGHFLSGDDTTSGRKRKDAD
NKHDDRELSDPSSTKKARVVWTVDLHQKFVKAVNQIGFDKVGPKKILDLMNVPWLTRENVA
SHLQKYRLYLSRLQKESDIKNSFIGMKHSDLPSKDSTASFGPHKAMNMIPDDVPNGTYSFSAS
NSLAQNVDLKGQGDLKGITSAPVAEPKGALSIDIHDSHEAKSTQMSFDHSLGSVDSAVSFASF
NSTTPLQYPWTEIPEIQFKQECEPLHLENGFSQLPLPGPSMIENEANRSRIEVKPLLDESRSNFVE
HLGPVGAEDLFPIQSKSQSLNNQVFDLISATKSSMKTQDVGLNYLADSEFALRNLNASGVGVP
LATLSEDLQICWLQGDCYPMNYGLQDLECSTNDNPALMAETPFHLYDVLRFDHEHLFDPTEY
YAIDQGLFA*
>GhARR49
MESKELNLNKELKAGGAGNGFIDRSKVRILLCDNDTKSCEEVFSLLLKCSYQVTTVRSARQVI
DALNAEGPDIDIILTEVDLPMTKGMKLLKYIMRNNELRRIPVIMMSAQDEVSIVVKCLRLGAA
DYLVKPLRTNELLNLWTHMWRRRRELGLLEKNILNCDFDLVASDPSDANTNSTTLFSDDTDE
RSRKSSNPEMGVSTHQEDESAAATVEPPQSDSPECRPDVPGISDRRTGQFSSVPKKSELKIGESS
AFFTYVKSSAVKTSSTQVATPNHESAAQNKISEENLPQPGEQVVTDTRVHENGETWENNSQG
DDFRSSSSVPDSLSLERSSTPASMEFSQQRDFKEDKFSPALVPPRNETQHDVSGLPTQSAYLHY
MPGVLNQVMMPSSTQLFQNNLHDIHNHTSSPVVPQYNHLQQCLPHPHVSGMASFPYYPVNM
CMQPGQMPTGHSWPSFGNSSSNEVQPSKVDRREAALIKFRQKRKERCFDKKIRYVNRKRLAE
RRPRVRGQFVRKNGITVDLNGQPASADYDEDEEEEQASRDSSPEDDTSGC*
>GhARR50
MVCTANDLSTWKDFPKGLKILLLDGDTNSANELKSKLEAMDYIVYAFHDENEAISAVSSRPES
FHVAIVEVSTDNNNGSFKFLETAKDLPTIMTSNIHCISTMMKCIARGAVEFLKKPLSEDKLRNI
WQHVVHKAFNTGGNDLTESLKPVKESVVSMLQQQPENLKPKNEDSETTEDASMIHENDSEPP
AGNDKYPAPSTPQLEQGGRLLDDRDCQDHTHCSIEKGSGDQDGEAESVETTGGNTTATGGQP
RGPSETNVKEEDDSVDGTMGESPQNRVDSKGSDVVAEKPSSCPDKANRKKSKVDWTPELHK
KFVQAVDQLGIDQAIPSRILELMEVEGLTRHNVASHLQKYRMQRRHILPKDDDRRWLQRPQT
QRICNYPHKPIMAFPPYNHVPVGPVYPMWRAPSHPPTIQMWGTQGYPPWQPTESWHWKPYP
GVQADAWGCPVMPPPQGYFSTFPQNASGFQPCNVDKRNGMPQTLVEHHPEEEVIDKMVKEAI
NKPWLPLPLGLKPPSTDSVLAELYRQGISTVPPHINASDR*
>GhARR51
MGEVVVTSEEVEEVKIDSETEEQENGEVEGNMRRRRRKKKKKKKKPGCSASGAVVNWERF

LPMPALRVLLVEADDSTRQIISALLRKCSYRVAAVPDGLKAWEMLKGRPHNVDLILTEVDLPS
ISGFALLTLIMEHDICKSIPVIMMSSQDSVSTVYKCMLKGAADYLVKPIRRNELRNLWQHVWR
RKSSIVGGNSPQDESIGQKKVETTSENNAASNHSIGCLDGVRKNKEQTEKGSDAQSSCTKPEM
EAESAQKENMQEFSHLIKVNSPPIESQKHEAHGSFNQNLLMHEMETEVVDSCKDAYTTLYKG
VELENQRRDTRVLVEAGDALVDSPREAIDFMGTFNKNCTSSSINSAKKFDSSLFLDLSLRRSNP
NVFENHVTQERPTLWHPSSSAFTRYTSRVSQPLHSTLMSFSDQKKDSGTNSEKMLTNVMSEN
NSDTPSPTLTSQRNTNPLTIGATVELKQTEVATPCTQHRLFPVPVPVKGIRLNNPCNGYNTIIPP
MFYARSSSSMAPSPSTANQQEPAFHLNLFRHSSFEANSSGQLYDRLASNTNQSTSQLLRKLDQ
KLDSIEDRGHISPTTDQSASSSFCNGSLSQLNGVAYGSTGASNGNVDQAAVTRASTESKNDDSF
PSPSGKPCRSIQREAALMKFRLKRKDRCFEKKVRYESRKKLAEQRPRVKGQFVRQVQADPMH
IETEHHYGNSSDG*

>GhARR52

MGNMATQTEFHVLAVDDSLIDRKLIERLLKTSSYQVTAVDSGSKALEFLGLNGENEDEERNSS
VESVSAADEDHQVGVNLIITDYCMPGMTGYDLLRKIKQSSSFKDIPVVIMSSENIPSRINRCLED
GAEEFFLKPVKLSDVNKLRPHLMKGITTKNEMQSNTNKRKGSEEIQSPDRTRPRYNELEVV*

>GhARR53

MGMVQMNNNSPVTSGLVELNTHICDEKMNIMDGVTGEGQGLSDEDESRINEDVENRNKGKK
AVVQRRGPLVCWERFLPLRSLKVLLVENDDSTRHVVCALLRNCGYEVTAVSNGLLAWKILED
LTNHLILF*

>GhARR54

MARNGGVAWMRTTEKIDGYDLSTSDTEEVHVLAVDDSLVDRKVIERLLRISSCKVTAVDSGR
RALQYLGLDEEIQKKETNGFDGLKVDLIITDYCMPGMTGYELLKKVKESSAFREIPVVIMSSEN
VIARIDRCLEEGAEDFIVKPVKLSDVKRIIDYTTTELREGEQREGARAKRRGINKRKQREGDDD
LSSSPPSTLSSSTSSSPSSSIQSATAPSSPSTLDSPTRRLKMTSSE*

>GhARR55

MTMEEKLGGSNGEDGGRDRFPIGMRVLAVDDDPICLKVLENLLRKCQYHVTTTNQAITALKM
LRENRNRYDLVISDVNMPDMDGFKLLELVGLEMDLPVIMLSAHSDTKLVMKGITHGACDYLL
KPVRIEELKNIWQHVVRKKKPDSKDQINASNQDKSRGGTGETGPTSSSSDQKVSKKRKDQSED
EDEEGDDNGHEDPSTQKKPRVVWSVELHRKFVSAVNQLGLEKAVPKKILDLMNVDGLTREN
VASHLQKYRLYLKRLSSVATQQANMVAALGSKDPSYLRMGSLDGFGDFRTLTGPGRISSASLS
SYQPGGLFGRLNSSAALSLRGISSGVIQSGHSQTLNNPINGFGKIQPAVVPANQNQNGTLFQGIP
TSINQLSQNKPTNHFGEFNRGNDPNAFGVATNFQDARVTVGGSSNTLPVSSGNPLLLQSNTQQ
TQHSGGAFGNQPSSLGVTSLNQEPFDMNVRGSSNFLDHGRCSENWQSAVQLSSFPSNALSASE
TFSHEQLPSNNLQESISWTSSHLSSSPLDLSSSMANPANLEDSRGNISQVGLNNNVIQNIDYTTK
QQWGDSRHDYNGNMNNSFSRGDSLVPASGPMMDQSNVISDKMNDVSLFSQFSGDSTYVVPH
LDGEKSAFDTKSRSNDDFLFETKPQNGFNQNSFEPLENIMMSMIKSDQNNETPLMDGEFGFDA
YSLGSCI*

>GhARR56

MRVERVISDENDSFPVGMRVLAVDDDPTCLLLLETLLTKCKYNVTTTSQAITALKMLRENKN
KFDLVISDVHMPDMDGFKLLELVGLEMDLPVIMLSANGDTKLVMKGITHGACDYLLKPVRIE
ELQNIWQHVIRRKKFDRKDQDGSGSQDKPYIDSSEAAGLGNVDQNGKFNKKRKDQNEDEDE
ERDENGHDNEDLSTQKKPRVVWSVELHRKFVAAVNQLGVEKAVPKKILELMNVENLTRENV
ASHLQKFRLYLKRISCVANQQANMAAALGSVDSTHLRMGTMNGLGNFHTLAGPDQIHNAAF
RSFQHSGVLRRLNTPAGVGISGLPSSGLVQLSPVQNLGNTSTYQRKLQSFVIPGNHNANILQGM

PMSLEFDQLQNNRAASHVGQFPSVDNTTVLPVSSISGSLIDAGCSNSPLLGVTSNSLLLEGSSQL
PTLHASNLRDSVSGFPNRSTLSDFTSIAPASNQLHGSKADIQDQAFPISCNAGKIISSAPQDWNA
PKKDAPYQSNVPSCSINSLRAVNGDMAQLGQCLDQNNPIFNRNVGFDFVDPLSIKCSEGENLA
MEPSVIEKEGYLMVQPSRQGIYIPDNEGENFAMEPSVIEKEGYLIVQPRRQGSYIPGNLGSLEDL
ASAMTKQDHDKGKPTDGDFGYNDYSLRTCI*
>GhARR57
MCHEQKEARHGVERDGQGSGSIGENGSRIVERTLNVNNGSLEAIEVHDVSEIPQQQPRGSMIR
WERFLPFRTIKVLLVENEDLTRHLVSALLQNCSYEVVAVANGLQAWKLLEDPTNHIDIVLTEE
DMPVLSGSDLLCMIMNHKMLKNIPVIMMSSHDCINLVFKCLSEGAVDFLVKPIRKNELKNLW
QHVWRRCHGSSGSGSVSESGTLSKKSIKSKVNDEPENYAANTDEHDDDSDVLVGCNGSENGS
GTQSSWTKRAAEGESSQLMSSLNRFPDAPNSTCAQVVHVKHEKCGSPWTCVTRRKECQEQHE
QLLDATEGKDLEVRVESNHEWQCGNQCENSPTHLAEAASKPFDRGWFEHQDENITGNDRTPD
IITTLQQAECRASDAPGGPSDVPQLKDGACHGSEEKLSFELTLKRWQGASDGRNAANDGHNV
LRHSDSSAFSKYSIASSAKQALTGNVGSCSPLDHSSVTKKTEIMCTFPSHSNGILLNQSSVGSNN
KNDMTTTAKFVGPKPKALVDKSGSISTFKCLHSSSFQPMHGCCICSSQEVSPETVGHDTSLKIM
AITDKQCRSSSSVNGSASGSKYGSNGHNGSETGLRAEHAVTEDGNGTASGRRGGSGADEDRV
AQRAAALTKFRQKRKERCFEKKVRYQSRKKLAEQRPRVKGQFVRRTVSDWGGKDSSSYDFT
SEDRYSDSLR*
>GhARR58
MELMKSENIVQDRQPQPQEEEEIMQQEEEEEQRFHVLAVDDSVIDRKLLEKLLKASSYQVTCV
ESGEKALEYLGLLHHSSPASSSSQHHHQVSIITLFYAKLIIKKIPTQSYTI*
>GhARR59
MFYQIMDGDYGGSCGGCSEDMTVEFGYEPHVLAVDDNLIDRTLVEKLLKNSSCKVTTAENGL
RALEFLGLGSDERNSLEGTVSKVNLVITDYCMPGMTGYELLKKIKESSVLKEVPVVIMSSENIP
TRINQCLEEGAQMFMLKPLKQSDVKQLKRHLMKYRS*
>GhARR60
MELMKSSDNIIQDRTQQQHKQEPEMQAQEGQQEQGHHFHVLAVDDSVIDRKLLEKLLKVSSY
QVTCVDSGDKALEYLGLLNNLDSDSTASSSSPSSSSSSSSSSSNSSSCSQSSQREGLKVNLIMTD
FCMPGMSGYDLLKRLKGSSWKDVPVVVMSSENVPSRISMCLEGGAEEFMLKPLQLSDLEKIE
AYLLKSLHLSCTNIDKDDDDNNADHNSDNNNDKVVNNDSNIDKGNNSTSMNNNFSKRKALS
SEDTESRRPKIKGLAVAI*
>GhARR61
MTMEEKMGGSNREDEALDRFPVGMRVLAVDDDPICLKVLGTLLLKCQYQVTTTNQAISALK
MLRENRNRYDLVISDVNMPDMDGFKLLELVGLEMDLPVIMLSAHSDTKLVMKGITHGACDY
LLKPVRIEELKNIWQHVVRRKKPDSKDRVNALNQDKASGGIGEAVQTSTSSSDQKFNKKRKD
QNEDEEDDGEDNEHENDDPSTQKKPRVVWSVELHRKFVAAVNHLGLDKAVPKKILDLMNVE
GLTRENVASHLQKYRLYLKRLSSVATQQANMVAALGGKDPSYLRMGSLDGFGDFRTLTGSG
RLSSASLSSYQPSGMFGRLNSSAALNLRGISSGVIQQGHPQTLSNSINGLGKIQPVGLPANQNQ
NGTLFQGIPTSIELNQLLQNKSANHFGELNPVNNPNVSGVATNFPDARMTVGSSSSSLSTPSGSP
LLLQANTQQAQRSGAFGNQSSLDVAFLNQESFDMGVHGFSNFADNGGCHENWQNTVQLSSF
PSNSLSTCEAFSHEQLHTNNLQESISWRSSHLNDNPIDLSASMANAGLEDSRGDMQCQISPSNN
VIHNIDYAAKQQWGENSSFAGADSLVAGAPYVQHLEAGKLAFNTKLRSNEDILFEQRKPQNE
FSQNNFETLDVISSPMIKPEQYNETGMMEGELGYDAFPLGSCI*
>GhARR62

MTKGMKLLKYIMRNNELRRIPVIMMSAQDEVSIVVKCLRLGAADYLVKPLRTNELLNLWTH
MWRRRREILLVHSFCRALSCSKGVSLVVGLFSLLIPSMAFTSSKVPNGSVVTKTIGNINHRGKI
YPITRGFDRRRLRNTGLLMAKLRFPNERLGNIYEKTGGFGVTRGSSLICLSSRTQKSETDCSDA
SSAQIAEDEVGHSSMHGRTIHSSPGLAEACRFACNDAKFVNERARNDIVLLSRGIMRLDARAR
QDVAILGSGFLKLDARAREDTEKIDRGVKKKAERLHHIATILKAKAESRLKKAADKHWSDGA
LEADLRHADFRAKQRAMEDALMALEFVKNIHDMMVSKGYKFPLRWEKGSLSANDLMLEKN
GKTLDFFNGEVSTDRISAIQEAYWSIASALSEADGIDYTDPEELELLLMTLIDLDAMDGKSSVS
LLQECSRSPDVNTRQALANALAAAPSMWTLGNAGMGALQRLAEDDNPAIAAAASKAIYELK
KQWEIEEGDSWRFMMNMKPSEDIDGDDEDN*
>GhARR63
MRVERIINETNDEFPIGIRVLAVDDDPTCLLLLETLLRRCKYNVTTTNQAITALKLLRQNKFDL
VICDVHMPDMDGFKLLELVGLEMDLPVIMLSANGDTNVVMKGITHGACDYLLKPVRIEELNN
IWQHVVRRKRFERKDWYNSDSQDKLRADSGEAVGMGSISNNGKLKKKRKDQDEDEERDEN
GHHNNEDPSIRKKPRIVWSAELHSKFVAAVNQLGMKNATPKKILELMNVEKLTTQNVGSHLQ
AFIRTFSANSYDDPKKFRLYLKRISCAANQQVNIAAAVRMRSPNGLGNFHTLAGSNQLHNAAF
RSFPPRGVLGRLNTHAGLLIRGLPSPRTIRSGHGQSSVNSGNDQSKLQSFVSGIHNANILQGLPM
SLELDQGQTNNGVSHIGELPIADSTTVFPVSSSLIDATITGFSGNPLLGVTSHSLMLEASSQQAS
NSRDIVPAIGFRNENTLSDFAPLAPASNRESNADLQCEPIPINCNAGELISSAPQEWNSPYQSNV
TPCSMNSSIPVSGTMVQFGLCLDQNNSMDSDSIGPSSFIDNSAMEQSIIDKEGYLMLEPWEQGS
HIPYNIGLLEDLWELQ*
>GhARR64
MTYVVGIKVIATTYTVCFFNAMDGGFDGDFPAGLKVLVVDDNRTCLLVLETMLRKLSYEVTT
CQLARHALALLREDKNRFDIVLCDLHMPEMDGLKLLEIIGLEMDLPVVMMSSDDGKGVIMKG
IIHGACDYLVKPVQMEAVRLIWQHVVRKRQRALGDFQQLRGNIHATGRTLLLKQAKNAVDQ
MPARERRILKRARENDDEDEDDEDDEGELSEEVTTAKKPRVIWTQELHDIFVIAVNQLRQRMN
NSLLLYLYHSLMEINMLITCLPEAVPKKILERMQAMNVTGLSRANIASHLQKYRLHLRKGGGQ
PLADNRDVNLNPSIGQASSFNQFNLQFQQPTATGSSCNQLPMQNLMITPQPCTINDSVVPDSTQ
CFPTELSTNDLSQPNLLFQNDVPTSNVEHLYRNVGVNVSELSNPISSLDDSSVNQLIYEPSVLVR
QYDQGDFFHGGFPRSDSYVSVRDIEYLISEEAPMKRISFFLRQMLVLYDLCSSLLFQADRIDSQF
S*
>GhARR65
MSGNGASSSSLNMISGEDVVNNNLSALVVDDSPLLRLLHDIHLKKYGLKVQVAENGKVAVDL
FHLGASFDLVLMDKEMPVMNGVEATKELRAMGVTSMIVGVTSKDGPGEQQAFMEAGLDYC
FEKPLTPEIISFLLEELNKHNNKN*
>GhARR66
MVCTRNDLSAWKDFPKGLRVLLLDEDTNSAAEIKSKLEAMDYIVYTFCNENEALSAVSSRPES
FHVAIVEVCTNSNNGGFKFLETAKDLPTIMTSNIHCISTMMKCIALGAVEFLRKPLSEDKLRNI
WQHVVHKAFSAGGDDLSESLKPVKDYVASMLHQQLENGECKHEDLDKIEDASMIHENDHEP
SAVNNKYPAPSTPQLQQGGRLIVNGDCQEHTNCSMEKESGEPDGESKSVETTSGHTIAEVIAPV
GQHQKPRETMVKEEADSVDGAKGERTLDSHPRDRVNSEDSHAGAEKPNTVSGLHSSRPNKA
NRKKLKVDWTTELHKKFVQAVDQLGIDQAIPSRILELMKVEGLTRHNVASHLQKYRMHKRHI
LPKEDDRRWPQRDQTQRSCHPHKPIMAFPPYHSNPVPVGPVYPMWGTPHPASIQTWGSQGYY
HPWQPTESWQWKPYPGVHADAWGCPVMPIPQGNCSTFTRNGYGFHCSSTMDNRSGMPQNSI
QHQPAEEVIEEVVKEAINKPWLPLPLGLKPPSTDSVLAELSRQGISTIPPRNQRL*

>GhARR67
MAQSKHGGRKLGQSNKMTALVVDDNMINRTIHHRLLENLGVENEVVSNGKEAVDIHYSGKM
FDLILMDMDMPIMNGIEATKKLREMGIRSVIAGVSSRAMEEEIREFMEAGLDDYQEKPLTMSK
LVSIIHKING*
>GhARR68
MEEKMGGSNGEDSGEDQFPVGMRVLAVDDDPICLKILANLLCKCQYQVTTTNQAIIALKMLR
GNKNRYDLVISDVNMPDMDGFKLLELVGLEMDLPVIMLSAHSDTKLVMKGITHGACDYLLK
PVRIEELKNIWQHVVRRKKPDSKDELAAPNHDKSRGGTGEAGQTWVACSSDQKVNKKRKDQ
SEDEEEETEDNGHENEDSSSQKKPRVVWSVDLHRKFVAAVNQLGLGDQAVPKKILDLMNVE
GLTRENVASHLQKYRLYLRRLSSVATQQANMVVALGSRGPSHLRTGSLDGFGDFRSFTGPRR
FSSASLPSYQSRGTFGRLNSSAALTLSGISSGVIQPGNSVNGLGKIQPVVLPANQNQNEALFQGI
TASIELNQLSQTKSTNHFGEYNCVNERNVFRISSSFPDARVVVGSSSNSLSTASGNALVLHGST
QEVQCSAAFENLPSFGMTSLNRESNDLSVRGSFNCLEHGRCSENWQGTVQLSNFPPSNEQLPS
NNLQESIPWRNSSPSNSRIALSSMASSAVLGDSRADILCKAGLNNSYSHVDSRVSASGSMMDQ
NNAVSSNTNDVSLFSHLNGEAPFAVRHSEGDKSSSDRFEVQ*
>GhARR69
MNVDGDGDKGLRELNHRLYDGSKRTTNGVVAEEHVMLEDVKVNKIAQNVKDGHVVAVQA
PAMRQITQQQPQNAMCYWQRFLHLTTVTVLLVENDDSTRHVVTALLRNCCYDVVEAANVLQ
AWKILEDLTNHIDLILAEVGMPSLSGLVLLSKIMSHKTRKNVPVIMMSSQDSMNLVFKCLSKG
AVDFLVKPIRKNELKNLWQHVWRRCHSSSGSGSESGTQTQKSVRSKSVEKSDNNSGSNDEEN
NGSIGLNIGDGSDDGSGTQNSWTKQAVEVDSPRLVSPSDQVAECPDSTCAQVVHSNAELSGN
KWVPVAAAKGCQEQDEQLDNVAVGKDLDIGMPRNLDLQLECPVEVPIRTVGAKQINLLDMS
SSKFSEQIEKRQLDLNSESPSNKQKSEAANQTGTTSKTTDLKKEIAENEVSNRLSKIPDVNDKTI
NDSKELPSVELGLKRLRGVKDAGTVVRDERNVLRRSDSSAFSRYNTASNANKVPVVNIGSSSA
LDSNLELTRKGSVCNNESHLVNELPNQSSNVGSNNIDMGSTTNNAFAKAAVDKNKSAASSTV
RSLHPSSIFQPMKNDLLSATQKVVFDKADDVSTTAGLAQARGIHHELQMQHPSNHYDQHHHL
THGMQQQQPPEHDDLSLKKLAADAPHCGSSNVWGGLVEGNAANYSVNGSASGSNHGSNGP
NGSSNAVNTVGTNMESDNGIAGKSGSGDVSGSGSGSGSGSKADQSKSAHREAALTKFRQKRK
ERCFQKKVRYQSRKRLAEQRPRIRGQFVRQTVNNDPASNGNSCDE*
>GhARR70
MEVAGEVLASESQLHVLAVDDSHVDRKVIEKLLKISSCKVTTVESGARALQFLGLDEEKGSLG
FNGLKVNLIMTDYSMPGMTGYELLKKIKRSSAFREIPVVIMSSENILTRIDSCLEEGAEEFLVKP
VKLSDVKRVMNDCIMRGETEGNNRRRVDKRKFEDDNHASSSSSSSSSIPSSPSSPLPSSPSTAAT
SSPLHSLKRAKLRDDN*
>GhARR71
MNSSSGKSYMSTASSSVLWKAGGDVVADQFPAGLRVLLVDDDPTCLMILEKMLKACQYNVT
KCNRAETALSMLRENRNGFDIVISDVHMPDMDGFKLLEHIGLEMDLPVIMMSADDGKNVVM
KGVTHGACDYLIKPVRIEALKNIWQHVVRKRKNEWKDFEQSGSVEEGDRQPKQSEDADYSSS
ANEGNWKSSKRRKDDEDENDERDDTSTLKKPRVVWSVELHQQFVAAVNQLGIEKAVPKKIL
ELMNVPGLTRENVASHLQKYRLYLRRLSGVSQHPSNLNNSFMNPQDATFGPLDLQALAATGQ
LPAQSLATLQAAGLGRSTAKSGIPMPLIDQRNIFSFENPKLRFGEAQQQHVSNNKQTNLLHGIP
TTMEPKQLAGLHHTAQSMGNMNMPVLSHGSQSSQNNPLLMQMAQPQARGQMLNDSTVGLA
PRLLSPRGQSMLSNGMATNVSTRNGVPENIRAPSYNPVSQTSLMLNFPMNHTSELPGNSFPLVS
TPGISSLAPKVAFQEDVNSEIKGSVGFMPSYDIFNDLNQHKPQNWELQNVGMMFNSSQHSNSL

QGNLDHTQSILVQQGFLSGQNRNSTVISKAMFSAGDSTGHVNAQNSNQHLNSLLDNTVRVKS
ERVADTAPANLYPDHFGQEDLMSALLKQQDIIAPAENNDFDFDGYSIDNIPV*
>GhARR72
MNVSSVKGSMSISSSTSTWKAGDTISDQFPAGLRVLVVDDDPTCLMILEKMLIACLYKVTKCN
RAETALSKLRENKNGYDIVLSDVHMPDMDGFKLLEHIGLEMDLPVIMMSADDGKQVVMKGV
THGACDYLIKPVRIEALKNIWQHVVRKRKNEWKEFEQSGSVEEGDRQPKQSDDADYSSSANE
GNWKGSKRRKDEEEETDERDDTSTLKKPRVVWSVELHQQFVAAVNQLGIDKAVPKKILELM
NVPGLTRENVASHLQKYRLYLRRLSGVSQHQSNLSTIISAQDPTFGSLSSLSGLDLQTLAATGQ
LPAQSLARLQAAGLGRATAKSAIPITLVDQRNIFSFENPKLRFGEGQQQHMTNKQQVNLLHGIP
TTMEPKQLVSLRHAAQSVGNMNMQVPPHGPQSGQNNPLLMQMGQQQQQQQQSRGQILVDS
TINHAPRLSSMGQPILSNGMATNVSSRSGIPENIRAPGYSQTPSMLNFPMNHASELPGNCFPLGS
TPGVSNLTSKGAFQEDVNSETKGSGGFMPSYDVFNDLNQHKPQSWELQNVGIAFDSSQHSNS
LQGNLDLTQSALGQQGFSSGQMNGHNRSAAVASKAMFSTGDVKELRSAQNINQHLNNLLVD
NTIRVKSERVCDTSPANIFPDHFGQDDLMSALLKQQESVASSENEFDFDGYSMNNIPV*
>GhARR73
MLSCGAGSGGCSKDMAVELGDEPHVLAVDDNLIDRKLVERLLKNSSCKVTTAENALRALDY
LGLGNDTLEGTVSEVNMIITDYCMPGMTGYELLKKIKESSVLKEVPVVIMSSENIPTRISQCLEE
GAKMFMLKPLKQSDVKQLKWHLMKCRN*
>GhARR74
MGMAIDRDDTQFHVLAVDDSLIDRKLIERLLKTSSFQVTAVDSGSKALEFLGLNNNDADDDEE
DEQRDSNTESVSSDEDQDHQDLGLGVNLIITDYCMPGMTGYDLLKKIKKSSSFKDIPVVIMSSE
NIPSRISRCLEDGAEEFLLKPVQLSDVNKLRPHLMKGRSKDIDSSHKTTSTRLITSGN*
>GhARR75
MGEVLMSSDEVKTESEIDRENGEFEGNKRRKKMSGGSFVSGEAVNWESFLPTMALRVLLVEA
DDSTRQIVTALLRKCNYRVSAVSDGLQAWEMLKAKPHNIDLILTEVDLPSISGFALLTLIMEHE
ICKSIPVIMMSSQHSISTVYKCMLRGAADYLVKPLRRNELRNLWQHVWRRQSLIANGNTPQDE
SVGQKKIEAISDNNAASNRSSGCLVGGERNKEQTEKGSEALSTCTKPDMEAEDANMENMQEF
LLLPSNSQKHEVQMNFNQRLLVHEKKTGVVGACKDTKISVETGDAVGDSPRKAIDFMGTFDR
NCNSSSMNSTSKVGSSTHLDFSLGRCSPNAFENHATREKPTLWHPNSSAFTRYSSRPSQPLQST
LTSVSDQKKESGTDSEKMLPNSIDEYNSDTPSPKLTPQRNTNPLTTGTTGQLRQTEVAASCTQQ
VVLPVLVPSPSLANQKELACCVNPFHHPSFESNSSGQFYDRLASNTNQLTNQPLQKLDQKMNS
TEDRGHISPTTDQSATSSFCNGSLSQLNGIAYGSTAASNNSNIDQEAVVRASADSKNDDVFPSP
TGNSHRSIQREAALTKFRLKRKDRCFEKKVRYESRKKLAEQRPRVKGQFVRQPQADLSHYGN
SSDG*
>GhARR76
MVEVGMEVDLEVGTAEETKKKKEEEENDESSEVVRWEKFLPQMVLRVLLVEADDSTRQIIAA
LLRKCSYIVAAVPDGLMAWETLKDRPHNIDLILTEVELPSISGFALLTLVMEHDICKNIPVIMMS
SEDSFSMVLKCMLKGAADFLIKPVRRNELRNLWQHVWRRHMLAGGCAHHNLPAKEHNVED
TAENNAAENNQSSDYGSSTQKIKDESDTQGLLQLKCMSSNVSDTGREQQGNFVKPGQESLQN
EGQAEGISDKLFVSKNSKRFVEVAHCSGTCESIASKLEENSAFVDEMAHNVVGLQSDKRIAYV
MIGVGCNDELRESSTGAIDLIDSFDKQPMATFAISSLSDGAKKLEFSPQLELSLRRPCSRSAKSQ
GTNEKHILNHSDASPFSWYNSRKSLLPIFLTLDGNREEGNSNPRQDLESNNGMRMDDVHAGC
NDVSPHFYHAKSSLPPACGPKQVGKQEYSPLPVSTSVHSDPDVDDSEQCYRWCDEATNSSND
QTLQGQNKQEPIEELRCSSLIDYQSACSRLCNDVENHEKSCTHGAVPSWSNASSVVTLAAAID

KGTTMETFNNSNCFIHDGLKGMDTHRSSQREAALMKFRLKRKDRCFEKKVYYYYYYYYSFWL
KVSTRMIPMVVCLC*
>GhARR77
MLELSSTLSSHVRFLLQSLTEANADSVFRELCQRDKEAAVLALGVIARGCINGLYPRLSEEAAE
KLAPIKLENQLPFFPILPENLGTARLVVDFDRWWTGEESVNVLLESTGQVTTVKSARQVIDAL
NVEGPDIDIILTEVDLQMTKGMKLLKHIMRNNELRRIPVIMMSAQDEVSIVVKCLRLGAADYL
VKPLRTNELLNLWTRMWRRRREVLCFLKQG*
>GhARR78
MAGEFPAGLKILIVDDDRTCLLVLERMLRKFSYQVTKCQQAREALALLRQDKNRFDIVLCDL
HMPDINGFELLQIIEVEMDLPVVMMSSDDGKGVVMKGIVHGACDYLVKPVRMEAIGLIWQH
VVRKKKKRFSGEITRSLPLQRADNAVPAMDKRSLKYRKRTSEDEDVAEDGESSEGKKPRMV
WTQELHDLFVAAVNELGRGNAVPKKILERMQAMNVTFLTRANIASHLQKYRMHLQKEGAVP
SSDSRDVNAYIDHRNLQFQPSPTTPYQLPMQNLITERANENVLSIAPSHVDGGSNIFNSNIASES
SCSLPTQVTLYDLYRANLLYQNDFPPISNGVAISNDNEFPPNDGVANSNDESLYCNVVDGTELS
NPFSAVAVDGTELSNPLSAVAVDGTELSNPLSAVEDLIHEPSFLVGQYDQQAFFRGPI*
>GhARR79
MMEMRNTNGVVAEELGTLEGDDLKVDKIAQNVKDCHVGVVQAPAVLQIQQQPQSASGCW
ERFLHQASIKVLLVENDDSTRHVVAALLRNCRYEVIEAASGLQAWKILEDLTNHIDLVLTEVF
MPCFSGIFLLSKIMSHKTRKNVPVIMMSSHDSIGIVFKCLSKGAVDFLVKPIRKNELKNLWQHV
WRRCHSSSGSGSGSESGTQTQKSEKSGSVENSDNSGSNDEEDNENNGLNVGDGSDDGSGTQS
SWTKQAAEIKSPSPVSPQDRVAECPDSTCAQVIHSNAEASGNKGVPATAPRGCQELDEQLDNV
PMGKGLDIRMAGSVDLQRERPVEVRIKTIGANQINLLEMSFNKLNEPIDKRQLDLNTKSSSGEL
NSEAAHQTGITSKTNDLKKESTEYEASNRISKISDGNDKTTDDSKEVLPSTELGFKRLRGAEDT
EAMLRDERNVLRRSNSSAFSRYNMASNANKFSFVNTGSSSARDSKLELTRKGSVCDVQSPLV
NDLSNQYSNVGSNNINMASTTDNAFAKPAVLKNKSASSSTFRLGHPSSAFQPMKNDLLNAAR
KPVLDKADGVTTKAGLKQPRLTHQELDMQDRLQHQQPTDHDTLSLKKMGADAPHCGSSNVL
GGPVPVEGNAGNYSVNGSNSGSNHASNGPHGSSTLADTVGTNIESDNGIAGKSGSGGSGDAS
GTGSGRGSGSKVDQSKSARREAALTKFRQKRKDRCFRKKVRYQSRKRLAQQRPRIRGQFV*
>GhARR80
MEEKRRSNCGEGFSVLVVDHDTTSLMLLTSMLQLFSYQVTTTEVESVAISLIEEAKDRFKLVM
ADVNMDDVNSLSFLRKLLKMNIPFILMSKEKGHKAAEKAVAYGASLHLEKPISKHDLKYLWQ
YAYGRPAKRAKYLHGNELRTYDPNKNVGERDEQKKMLNIMPPTEKKPRLLWNEELHQKFIA
AITALGYANARPKSILTMMDEPNLTQRQVASHLQKHKEHMKRLMSKASPSELASNANAPTSI
YGKPGFLYMPQGYYSVLSQLGGHTHKYLNDSVAGTCQFSYLPQHSTADHFSSEYHNEETLQF
PNILESMHEKQNIIPGPSPSGNLVYENHNWEPWTEAARGVAEAEIGVGNAAGGVTWNQQSPE
LAQLLKHLEEEGDDCGDCANQPHPDAVDQFCEWLKEAIQGNDNNP*
>GhARR81
MAISVESNMNNVYAGVTVLLVDGDSTCLIILSKMLRSFGYKVVTTKRATDALCIIRDQQHKID
LVLTEACLHDMDKYELLETIRNISSLPIIVMSTDYDRNAVLGSLFKGAALHLEKPITMDDIKNL
WQFTLIKGREINVPIIEAKSCIKEVSSIESALGVVVDGRRNLRDEKRKRPLEVENDKEGDNWDQ
GSSTLKKPKLIWTNELHNRFLQAIDMLGSEAYPKKILQLMNVPGLRKENVSSHLQKHRLSLKR
QQEAILNTISSTESQAASHHALSEFSPRNGFHLFTDTTQTTSVAEQHGYINGLVQDNLNG*
>GhARR82
MICTTNDLSAWKDFPKGLKVLLLDEDSNSAAELKSKLEAMDYIVYTFCNENEALSAVSSRPES

FHVAIVEINTNKKNGSFKFLETAKDLPTIMTSNIHCISTVMKCIALGAVEFLKKPLSEEKLRNIW
QHVVRKAFNAVGTDLSESVKPVEESLVSMLYLQLENGEPKDKDLDKTQDASVIHENDPEPST
GSDKYPAPSTPQIEQGGRLSANGDCQDHANCSIEKESSEQDGESKSVETTSDNTIAEVTIPVGQP
QGPRDTMVTEEADLVDGTKGKSTTYSQTENGVNSKNSQAVAEKPSTVSGIHSSCLNKANRKK
SKVDWTPALHKKFVQAVDQLGIDQAIPSRILELMKIEGLTRHNVASHLQKYRMHRKHILPKED
DRRWPQRDQTQRSCYPHKPIIAFPPHYSNHVVPVGPLYPMWGAPPYPSSIQMWGSQGYPLWQ
PTESWHWKPYPGVHADAWGCPVMPPPHGYSSAFTQVSSYQNASVFHCSGTMDNRSGMPPNS
VEHQPAEEVIDKVVKEAINKPWLPLPLGLKPPSTDTVLAELSKQGISTFPPHIYGLNSSCHGTT*
>GhARR83
MGDFVVMSCEEVKEVKGNSDTEMENGEFEGIKKKKKKQQQESCVYGEVVKWERWMALRV
LLIEADDSTRQIIAALLKKCNYKVSAVPDGLKAWEMLKGKPHNYDLILAEVDLPSISGFALLTL
IMEHELCKSIPVIMMSSQDSVSTVYKCMLRGAADYLVKPVRRNELRNLWQHAWRRQSSIIGG
NFPGDESIGRKQVEATSENDAARQTDKGSDTQNSCIKPDMEAESADMETIQEFSDLIKGKSQPS
GSQMHEAHDCLNQNLLMHETKTSVNACKDPNLTAAYKGVELECQRTNMNISVEAGNALADS
PREAIDFMGTFNRNFNFSSINSTSKFDSSPPLDLSLRRCNNNDFENNVARERPILWHPNSSAFTR
YSNRLSQPQHSTLTSISNKKKESGSNSETILSNIVSEHDAATPSPTVTSQRNMIPSTAGATDQSR
HTEVATSCMEQRECPSSVNQHEPTFGVNPFHHSSLEMNSSRQFYDRLASGTNQMDQKLDSVE
DKGHISPTVNQSGTGSFCNGSISQLNGIAYGSGSASNSNVDQVIVRASTERKNDDNVPAPAGNS
HRSIQREAALTKFRLKRKDRCYEKKVRYESRKKLAEQRPRVKGQFVRQTQVDHPTQIQAECH
YHNSPDG*
>GhARR84
MVSTRSVNSPAMKITILVVDDDSTSLAIVSAMLREFRYEVTSVKTPAAALSVLRSNPSIDLVVT
DLHMPGMNGIELQKRINKEFKLPVIIMSSDDDENVMLESLAGGAVFFIVKPVDPVGLKNVWQ
YAVAAKKGKSLLIEDMDRESSSSSPADGKLSLGGNTKSVSSVNNEKNDPKNGSKRKGASGKG
RDKDDNDDESKSPPPKKTKKPKIVWTNTLHNQFLEALRQIGLERAVPKKILERMNSTGLTREN
VASHLQKYRIFLKRLAERGCFASKAVIDRFLKSNFAAGHPLLLKTAQEYSRLEHMERLRVLAS
YPGLRESLMGHSSTGSVPLFYGHPGASSSNAAQQPLGYGQSRLLSNQTNRPLFPGSGNMLNNP
YLNNRLGYGNGNGIGIGIGIGTSSSVNGGGGFSSGLLNGGNSSLTYPNQVQARPDFYNAGPSSS
SPFRFGSAGFHSSGSTLGNGLFGSGSSSYPSLNSSSSGSTVVNGLFGSSSRSHPSLNSSSSGATLG
NGLFGSSSSSYPSLNNSSYPSLNPGYTNNAANSYRGMRFYEHLLNGSAPPLTGDYGSMNQTRP
TGDYGSMNRTHNENINVPTMRTTPLDSLDFMREFPIPPVDSSTLIQGLGTGNTRLTEINSDQLL
NNVPNLGNEPRGGDGLLQDLVLESKKLANKEKAGEQSVENSDYCLPSLFPEIYPTLDELLNCD
FPDPLSDEDNHPWSEEAIGQQQTGGEQVTVTHPLSNVLQVMTAPAPAPAPAPAPAPAPALAVY
NLANNANNGAPVGANSSSYEDDEDFLDSLLNFNDEFE*
>GhARR85
MVMAASETKFHVLAVDDTLIDRKLIEKLLKTSSYQVTAVDSGTKALEFLGLNNNDHQDVEVN
LIITDYCMPGMTGYDLLRKIKESSSFKDIPVVIMSSDNIPSRINRCLEGGAEEFFLKPVQLSDVNK
LRPHLMKRTQTNVNKRKAMDEIVSPDRTRARYNELEVN*
>GhARR86
MTVEQIVSEGKDQFPIGMRVLAVDDDPTCLLLLGTLLRRCQYHVTTTSQAKTALKMLRENKN
KFDLVISDVHMPDMDGFKLLEHVGLEMDLPVIMLSANGDTKLVMKGITHGACDYLLKPVRIE
ELQNIWQHVVRRKKKDRCNSGSKDKPHPDSGEDAGIGNVDNNGKLNKKRKDQNEDEDDER
DENGHDNEDPSAQKKPRVVWSVELHRKFVAAVNQLGIDKAVPKKILELMNVEKLTRENVAS
HLQKFRLYLKRISCVANQQANMAAALGTADSAYLRMGSLNGLGNFHTLAGSDQLHNAAFRS

FPPSGVLGRLNTPAGLGIRGLPSPGTIQLGHVQNSGNPTNDLSKLQSFVPGNHNTNILQGMPMS
LELDRLQHNKSVGHIGELPTTDSTTVFPGSGSLVDARITGFSNNPLLGVTSNSLMLEGSSQQAT
SHTSVSAIGFQNGNALSDFTLIAPASNQLQDSKADSQGQASLINCNAGQIIRSAPQEWNAPRKD
APYQSHALINSSIPINSAMIQLGQCLDRNNSIFHRTTDLDSVGPLNFVDPLSIKHSEGDNYIMEPS
VIEKEGYLMFQPRPHGSHVPDNTGSLKDLASAMMKQEDDFGCNGYSLRTSI*

Arabidopsis thaliana

>ARR3-A
MNFEFIFLYLFRLVRYFLLPPSQKRKKGTYRFVSLIVTWNKKIFCKRNEVENDPPIKRFDIEEYN
NENIKQSREELVAISLFYLFISYSFLTMAKDGGVSCLRRSEMIGIGIGELESPPLDSDQVHVLAV
DDSLVDRIVIERLLRITSCKVTAVDSGWRALEFLGLDDDKAAVEFDRLKVDLIITDYCMPGMT
GYELLKKIKESTSFKEVPVVIMSSENVMTRIDRCLEEGAEDFLLKPVKLADVKRLRSYLTRDV
KVAAEGNKRKLTTPPPPPPLSATSSMESSDSTVESPLSMVDDEDSLTMSPESATSLVDSPMRSP
GLA*

>ARR4-A
MARDGGVSCLRRSEMMSVGGIGGIESAPLDLDEVHVLAVDDSLVDRIVIERLLRITSCKVTAV
DSGWRALEFLGLDNEKASAEFDRLKVDLIITDYCMPGMTGYELLKKIKESSNFREVPVVIMSS
ENVLTRIDRCLEEGAQDFLLKPVKLADVKRLRSHLTKDVKLSNGNKRKLPEDSSSVNSSLPPPS
PPLTISPESSPPLTVSTESSDSSPPLSPVEIFSTSPLSSPIDDEDDDVLTSSSEESPIRRQKMRSPGLD
*

>ARR5-A
MAEVLRPEMLDISNDTSSLASPKLLHVLAVDDSMVDRKFIERLLRVSSCKVTVVDSATRALQY
LGLDGENNSSVGFEDLKINLIMTDYSMPGMTGYELLKKIKESSAFREIPVVIMSSENILPRIDRC
LEEGAEDFLLKPVKLADVKRLRDSLMKAEERAFKNIMHKRELEANDIYSQLKRAKI*

>ARR6-A
MAEVMLPRKMEILNHSSKFGSPDPLHVLAVDDSHVDRKFIERLLRVSSCKVTVVDSATRALQ
YLGLDVEEKSVGFEDLKVNLIMTDYSMPGMTGYELLKKIKESSAFREVPVVIMSSENILPRIDR
CLEEGAEDFLLKPVKLSDVKRLRDSLMKVEDLSFTKSIQKRELETENVYPVHSQLKRAKI*

>ARR7-A
MAVGEVMRMEIPAGGDLTVTTPELHVLAVDDSIVDRKVIERLLRISSCKVTTVESGTRALQYL
GLDGGKGASNLKDLKVNLIVTDYSMPGLSGYDLLKKIKESSAFREVPVVIMSSENILPRIQECL
KEGAEEFLLKPVKLADVKRIKQLIMRNEAEECKILSHSNKRKLQEDSDTSSSSHDDTSIKDSSCS
KRMKSESENLFSLL*

>ARR8-A
MVMETESKFHVLAVDDSLFDRKMIERLLQKSSCQVTTVDSGSKALEFLGLRVDDNDPNALST
SPQIHQEVEINLIITDYCMPGMTGYDLLKKVKESAAFRSIPVVIMSSENVPARISRCLEEGAEEFF
LKPVKLADLTKLKPHMMKTKLKKESEKPVAIEEIVVSKPEIEEEEEESSVIEILPLHQEIESEQLE
PMLSSNKRKAMEEVVSTDRSRPKYNDITTSV*

>ARR9-A
MGMAAESQFHVLAVDDSLFDRKLIERLLQKSSCQVTTVDSGSKALEFLGLRQSTDSNDPNAFS
KAPVNHQVVEVNLIITDYCMPGMTGYDLLKKVKESSAFRDIPVVIMSSENVPARISRCLEEGAE
EFFLKPVRLADLNKLKPHMMKTKLKNQKLEEIETTSKVENGVPTAVADPEIKDSTNIEIEILPLQ
QDLLLVQQEEQTLSINNKRKSVEEGISTDRARPRFDGIATAV*

>ARR15-A
MALRDLSSSLSSSSSPELHVLAVDDSFVDRKVIERLLKISACKVTTVESGTRALQYLGLDGDNG

SSGLKDLKVNLIVTDYSMPGLTGYELLKKIKESSALREIPVVIMSSENIQPRIEQCMIEGAEEFLL
KPVKLADVKRLKELIMRGGEAEEGKTKKLSPKRILQNDIDSSPSSSTSSSSSSHDVSSLDDDTP
SSKRIKLESRG*
>ARR16-A
MNSSGGSCSSLMDVVAYDHHLHHGHDEELHVLAVDDNLIDRKLVERLLKISCCKVTTAENAL
RALEYLGLGDQNQHIDALTCNVMKVSLIITDYCMPGMTGFELLKKVKQESSNLREVPVVIMSS
ENIPTRINKCLASGAQMFMQKPLKLADVEKLKCHLMNCRS*
>ARR17-A
MNKGCGSGSDSCLSSMEEELHVLAVDDNLIDRKLVERILKISSCKVTTAENGLRALEYLGLGD
PQQTDSLTNVMKVNLIITDYCMPGMTGFELLKKVKQESSNLKEVPVVILSSENIPTRINKCLAS
GAQMFMQKPLKLSDVEKLKCHLLNCRS*
>ARR1-B
MMNPSHGRGLGSAGGSSSGRNQGGGGETVVEMFPSGLRVLVVDDDPTCLMILERMLRTCLY
EVTKCNRAEMALSLLRKNKHGFDIVISDVHMPDMDGFKLLEHVGLEMDLPVIMMSADDSKS
VVLKGVTHGAVDYLIKPVRMEALKNIWQHVVRKRRSEWSVPEHSGSIEETGERQQQQHRGG
GGGAAVSGGEDAVDDNSSSVNEGNNWRSSSRKRKDEEGEEQGDDKDEDASNLKKPRVVWS
VELHQQFVAAVNQLGVEKAVPKKILELMNVPGLTRENVASHLQKYRIYLRRLGGVSQHQGN
LNNSFMTGQDASFGPLSTLNGFDLQALAVTGQLPAQSLAQLQAAGLGRPAMVSKSGLPVSSIV
DERSIFSFDNTKTRFGEGLGHHGQQPQQQPQMNLLHGVPTGLQQQLPMGNRMSIQQQIAAVR
AGNSVQNNGMLMPLAGQQSLPRGPPPMLTSSQSSIRQPMLSNRISERSGFSGRNNIPESSRVLPT
SYTNLTTQHSSSSMPYNNFQPELPVNSFPLASAPGISVPVRKATSYQEEVNSSEAGFTTPSYDM
FTTRQNDWDLRNIGIAFDSHQDSESAAFSASEAYSSSSMSRHNTTVAATEHGRNHQQPPSGMV
QHHQVYADGNGGSVRVKSERVATDTATMAFHEQYSNQEDLMSALLKQEGIAPVDGEFDFDA
YSIDNIPV*
>ARR2-B
MVNPGHGRGPDSGTAAGGSNSDPFPANLRVLVVDDDPTCLMILERMLMTCLYRVTKCNRAE
SALSLLRKNKNGFDIVISDVHMPDMDGFKLLEHVGLEMDLPVIMMSADDSKSVVLKGVTHGA
VDYLIKPVRIEALKNIWQHVVRKKRNEWNVSEHSGGSIEDTGGDRDRQQQHREDADNNSSSV
NEGNGRSSRKRKEEEVDDQGDDKEDSSSLKKPRVVWSVELHQQFVAAVNQLGVDKAVPKKI
LEMMNVPG*REKT*PVTSRSIGYI*DGLEEYRNTKEI*TIRL*LVKIRVLDLFLR*MDLIFNL*LLL
VSSLLRALHSFKQLVLAGLHSLNQGCRFLPL*IREASSTLKTQK*DLETDMVRR*TMEICFMVS
QRVVTCVCVLDRMFRAAE*CCQ*QTSYLEEDHRCYHPSGNSRYCQAAFQEEAISLVRWRLET
VSPRPTAECYQLLTRSSITSPRIYLAAASRWQVPQGFQFQYQFLTKKRSTARMQKEVHQLLLL
DLVTQATTYLTIFRSTNSTTRTSAIN*TIGICGIWDWSSVPIRTQQLQPQPQHFPLRKHTLRLLRR
EKDGKRTQQLWVSMGRTCSHRAGICII*TTFLWTVVQSE*SQKEWRRQ*LVLQQIHCFTSSIIK
KI**AHFSNRKASHP*ITSSNLTDTPSIISRS*
>ARR10-B
MTMEQEIEVLDQFPVGMRVLAVDDDQTCLRILQTLLQRCQYHVTTTNQAQTALELLRENKNK
FDLVISDVDMPDMDGFKLLELVGLEMDLPVIMLSAHSDPKYVMKGVKHGACDYLLKPVRIEE
LKNIWQHVVRKSKLKKNKSNVSNGSGNCDKANRKRKEQYEEEEEEERGNDNDDPTAQKKPR
VLWTHELHNKFLAAVDHLGVERAVPKKILDLMNVDKLTRENVASHLQKFRVALKKVSDDAI
QQANRAAIDSHFMQMNSQKGLGGFYHHHRGIPVGSGQFHGGTTMMRHYSSNRNLGRLNSLG
AGMFQPVSSSFPRNHNDGGNILQGLPLEELQINNNINRAFPSFTSQQNSPMVAPSNLLLLEGNP
QSSSLPSNPGFSPHFEISKRLEHWSNAALSTNIPQSDVHSKPDTLEWNAFCDSASPLVNPNLDTN

PASLCRNTGFGSTNAAQTDFFYPLQMNQQPANNSGPVTEAQLFRSSNPNEGLLMGQQKLQSG
LMASDAGSLDDIVNSLMTQEQSQSDFSEGDWDLDGLAHSEHAYEKLHFPFSLSA*
>ARR11-B
MEKSGFSPVGLRVLVVDDDPTWLKILEKMLKKCSYEVTTCGLAREALRLLRERKDGYDIVISD
VNMPDMDGFKLLEHVGLELDLPVIMMSVDGETSRVMKGVQHGACDYLLKPIRMKELKIIWQ
HVLRKKLQEVRDIEGCGYEGGADWITRYDEAHFLGGGEDVSFGKKRKDFDFEKKLLQDESDP
SSSSSKKARVVWSFELHHKFVNAVNQIGCDHKAGPKKILDLMNVPWLTRENVASHLQKYRL
YLSRLEKGKELKCYSGGVKNADSSPKDVEVNSGYQSPGRSSYVFSGGNSLIQKATEIDPKPLAS
ASLSDLNTDVIMPPKTKKTRIGFDPPISSSAFDSLLPWNDVPEVLESKPVLYENSFLQQQPLPSQ
SSYVANSAPSLMEEEMKPPYETPAGGSSVNADEFLMPQDKIPTVTLQDLDPSAMKLQEFNTEA
ILRSLNWELPESHHSVSLDTDLDLTWLQGERFLANTGLQFQDYSSSPSLLSELPAHLNWYGNE
RLPDPDEYSFMVDQGLFIS*
>ARR12-B
MTVEQNLEALDQFPVGMRVLAVDDDQTCLKILESLLRHCQYHVTTTNQAQKALELLRENKN
KFDLVISDVDMPDMDGFKLLELVGLEMDLPVIMLSAHSDPKYVMKGVTHGACDYLLKPVRIE
ELKNIWQHVVRSRFDKNRGSNNNGDKRDGSGNEGVGNSDQNNGKGNRKRKDQYNEDEDED
RDDNDDSCAQKKQRVVWTVELHKKFVAAVNQLGYEKAMPKKILDLMNVEKLTRENVASHL
QKFRLYLKRISGVANQQAIMANSELHFMQMNGLDGFHHRPIPVGSGQYHGGAPAMRSFPPNG
ILGRLNTPSGIGVRSLSSPPAGMFLQNQTDIGKFHHVSSLPLNHSDGGNILQGLPMPLEFDQLQT
NNNKSRNMNSNKSIAGTSMAFPSFSTQQNSLISAPNNNVVVLEGHPQATPPGFPGHQINKRLE
HWSNAVSSSTHPPPPAHNSNSINHQFDVSPLPHSRPDPLEWNNVSSSYSIPFCDSANTLSSPALD
TTNPRAFCRNTDFDSNTNVQPGVFYGPSTDAMALLSSSNPKEGFVVGQQKLQSGGFMVADAG
SLDDIVNSTMKQEQSQGDLSGGDLGYGGFSSLRTCI*
>ARR14-B
MPINDQFPSGLRILVVDDDTSCLFILEKMLLRLMYQGLILYQSSHLLIDFLKKVLTFLFYLLFVV
TICSQADVALTILRERKDSFDLVLSDVHMPGMNGYNLLQQVGLLEMDLPVIMMSVDGRTTTV
MTGINHGACDYLIKPIRPEELKNIWQHVVRRKCVMKKELRSSQALEDNKNSGSLETVVVSVSE
CSEESLMKCRNKKKKKKRSVDRDDNEDDLLLDPGNSKKSRVVWSIELHQQFVNAVNKLGID
KAVPKRILELMNVPGLSRENVASHLQKFRLYLKRLSGEASQSNDSESTKRYENIQALVSSGQL
HPQTLAALFGQPIDNHHSASFGVWIPNDNLGRSQNEHFSVDVSSASNRPVSVAVHGLSSSANF
RQRGDVNNNRIRQGYGSNVNEES
WILERSSRQR*
>ARR18-B
MEFGSTEDGRHDKFPVGMRVLAVDDNPTCLRKLEELLLRCKYHVTKTMESRKALEMLRENS
NMFDLVISDVEMPDTDGFKLLEIGLEMDLPVIMLSAHSDYDSVMKGIIHGACDYLVKPVGLKE
LQNIWHHVVKKNIKSYAKLLPPSESDSVPSASRKRKDKVNDSGDEDDSDREEDDGEGSEQDG
DGSGTRKKPRVVWSQELHQKFVSAVQQLGLDKAVPKKILDLMSIEGLTRENVASHLQKYRLY
LKKIDEGQQQNMTPDAFGTRDSSYFQMAQLDGLRDFTAARQIPSSGLLSRSHLTKLQPPMYSSI
NLQGMNSSSFIQQGHHQNSSNSANPFGTYHSTLSPRIQNVNLFQRTSSPLEPLQFPRSKSYIGDF
KGLGDRAIGGSFLDTCMPFGSSSTSLPSASTNPLMLQANYTQPLHIASDGIQPCIEGTPSNSASP
NISFQGLSRFPGHSWQGNLNTTRFPPSSLPLNLAFLPDQVTCAGNNLGDCTSLVSAENPGGEM
QCDPQLLGGFMQNVNPLGGQKWEQQNCTMLNNPFGNIEYPLPADNMVFRDNNSTRSKGLDE
SLMNPIDNSQEYVGKATTMLDPEMKSGKPENDNQHDVFDDIMNEMMKQEENNGMVPVATR
FGFDSFPPP*

>ARR19-B
MLVGKISGYEDNTRSLERETSEITSLLSQFPGNTNVLVVDTNFTTLLNMKQIMKQYAYQVSIET
DAEKALAFLTSCKHEINIVIWDFHMPGIDGLQALKSITSKLDLPVVIMSDDNQTESVMKATFYG
ACDYVVKPVKEEVMANIWQHIVRKRLIFKPDVAPPVQSDPARSDRLDQVKADFKIVEDEPIIN
ETPLITWTEEIQPVQSDLVQANKFDQVNGYSPIMNQDNMFNKAPPKPRMTWTEVIQPVQSNLV
QTKEFGQLNDYSQIMNQDSMYNKAATKPQLTWTEEIQPVQSGLVQANEFSKVNGYSQSMNQ
DSMFNKSATNPRLTWNELLQPVQSDLVQSNEFSQFSDYSQIMNEDNMFNKAAKKPRMTWSE
VFQPVQSHLVPTDGLDRDHFDSITINGGNGIQNMEKKQGKKPRKPRMTWTEELHQKFLEAIEII
GGIEKANPKVLVECLQEMRIEGITRSNVASHLQVKKKTHTLNIKHRINLEENQIPQQTQGNGW
ATAYGTLAPSLQGSDNVNTTIPSYLMNGPATLNQIQQNQYQNGFLTMNNNQIITNPPPPLPYLD
HHHQQQHQSSPQFNYLMNNEELLQASGLSATDLELTYPSLPYDPQEYLINGYNYN*
>ARR20-B
MSVFSNILDENSRNLRNEIPCDDGIASPINDDDEEFLTKSNRVLLVGADSNSSLKNLMTQYSYQ
VTKYESGEEAMAFLMKNKHEIDLVIWDFHMPDINGLDALNIIGKQMDLPVVIMSHEYKKETV
MESIKYGACDFLVKPVSKEVIAVLWRHVYRKRMSKSGLDKPGESGTVESDPDEYDDLEQDNL
YESNEEGSKNTCDHKEEKSPTKKPRMQWTPELHHKFEVAVEKMGSLEKAFPKTILKYMQEEL
NVQGLTRNNVASHLQKYRQSSKKTCTPQEPQEDFVWGNAGPDVTLAASKTLLSSHATPSYLI
NNQAAPRGSYFMNNIPYPSTSCLPVNNNNCFMTNPSTYIDQFQHQLQQQQHQQYQSTLNSIS
AMLTKQESRHVPSSAMENSEPLMIYNSNLPFGIDECFPPAGFNIFDQIGHN*
>ARR13-B
MAFAQSVYNQSSVLKINVMVVDDNRVFLDIWSRMLEKSKYREITVIAVDYPKKALSTLKNQR
DNIDLIITDYYMPGMNGLQLKKQITQEFGNLSVLVMSSDPNKEEESLSCGAMGFIPKPIAPTDLP
KIYQFALTYKRNGKSTLSTEQNQKDANVSVPQQIMLVPEQAYVLKTKKKNCSSKSDTRTVNS
TNVSHVSTNGSRKNRKRKPKGGPSDDGESLSQPPKKKKIWWTNPLQDLFLQAIQHIGYDKVV
PKKILAIMNVPYLTRENVASHLQKYRLFVKRVVHQGRFSMLSDRGKDSMFRQTHIKEPYVNY
YTPSTSWYETSLNNRSFYSESVHGHSRLLSEAREPVRYNQMSYNYMNRNISFENQPSQNEETR
TVFEPPVMANKISQTSQVLGFGQLGPSAISGHNFNTNMMSSYGSLTPNQPGTSHFSYGMQSVL
NNENATYNPQPPANATTQPNLDELPQLENLNLYNDLGNTSELPYNISNFQSDDNKKQGEEDG
DWTFVNINQDQSNGESSNTIATPETNTPNFNINPNQNQGQAVPEFTDWSFLDQQELVDDDFMN
SLFNNDMN*
>ARR21-B
MASAQSFYNQSSVLKINVMVVDDDHVFLDIMSRMLQHSKYRDPSVMEIAVIAVDDPKKALST
LKIQRDNIDLIITDYYMPGMNGLQLKKQITQEFGNLPVLVMSSDTNKEEESLSCGAMGFIPKPI
HPTDLTKIYQFALSNKRNGKSTLSTEQNHKDADVSVPQQITLVPEQADVLKTKRKNCSFKSDS
RTVNSTNGSCVSTDGSRKNRKRKPNGGPSDDGESMSQPAKKKKIQWTDSLHDLFLQAIRHIGL
DKAVPKKILAFMSVPYLTRENVASHLQKYRIFLRRVAEQGLYSMLSDRGIDSMFRQTHIKEPY
FNYYTPSTSWYDTRLNNRSFYSKPVHGFGQSKLLSTTREPVCFNQMPYNYMNRSSTYEPHRIG
SGSNLTLPIQSNLSFPNQPSQNEERRSFFEPPVMANKIAQTSQVLGFGQLGPSAISGHNFNNNMT
SRYGSLIPSQPGPSHFSYGMQSFLNNENVTYNPQPPANATTQPNLDELPQLENLNLYNDFGNTS
ELPYNISNFQFDDNKHQQGEADPTKFELPAAKFSTELNHEDDGDWTFVNINQGQSNGETSNTI
ASPETNTPILNINHNQNQGQDVPEFNDWSFLDPQELVDDDFMNSLFNNDMN*
>ARR23
MTAKDSSEPVTTLTQFNNIDVVITDYHMPGLNGVQLKKRIDEEFGNLPVIDLYRNIEHEELFRR
ALCFMHKPISRRDLNSLNVVCQQALRHRMNGETNLNGSGQNVTDDPDDWLRLREKPKLKWT

KPLQHRFMSALKSLGVASKY*
>ARR22-C
MATKSTGGTEKTKSIEVKKKLINVLIVDDDPLNRRLHEMIIKTIGGISQTAKNGEEAVILHRDGE
ASFDLILMDKEMPERDGVSTTKKLREMKVTSMIVGVTSVADQEEERKAFMEAGLNHCLEKPL
TKAKIFPLISHLFDA*
>ARR24-C
MTRDQVAEELPNLAESKLTALVVDDSFVNQTIHQKLLNRLGIKNDVVTNGKEAVDVYCSGGN
YDLILMDMDMPIMNGIQATKRLREMGIESKIAGVTTRANEGEKKEFMEAGLNDFQEKPLTISK
LLSILHKLNFYVQT*
>APRR1
MDLNGECKGGDGFIDRSRVRILLCDNDSTSLGEVFTLLSECSYQVTAVKSARQVIDALNAEGP
DIDIILAEIDLPMAKGMKMLRYITRDKDLRRIPVIMMSRQDEVPVVVKCLKLGAADYLVKPLR
TNELLNLWTHMWRRRRMLGLAEKNMLSYDFDLVGSDQSDPNTNSTNLFSDDTDDRSLRSTN
PQRGNLSHQENEWSVATAPVHARDGGLGADGTATSSLAVTAIEPPLDHLAGSHHEPMKRNSN
PAQFSSAPKKSRLKIGESSAFFTYVKSTVLRTNGQDPPLVDGNGSLHLHRGLAEKFQVVASEGI
NNTKQARRATPKSTVLRTNGQDPPLVNGNGSHHLHRGAAEKFQVVASEGINNTKQAHRSRGT
EQYHSQGETLQNGASYPHSLERSRTLPTSMESHGRNYQEGNMNIPQVAMNRSKDSSQVDGSG
FSAPNAYPYYMHGVMNQVMMQSAAMMPQYGHQIPHCQPNHPNGMTGYPYYHHPMNTSLQ
HSQMSLQNGQMSMVHHSWSPAGNPPSNEVRVNKLDRREEALLKFRRKRNQRCFDKKIRYVN
RKRLAERRPRVKGQFVRKMNGVNVDLNGQPDSADYDDEEEEEEEEEEENRDSSPQDDALGT*
>APRR3
MCFNNIETGDEVETERQVFGSSEEDEFRVEDTARNTNNVQISQQQQQPLAHVVKWERYLPVR
SLKVLLVENDDSTRHIVTALLKNCSYEVTAVPDVLEAWRILEDEKSCIDLVLTEVDMPVHSGT
GLLSKIMSHKTLKNIPVIMMSSHDSMVLVFKCLSNGAVDFLVKPIRKNELKNLWQHVWRRCH
SVRVLIWLPVLQKLLCVFFLLMRLLIYQSSGSGSESGIHDKKSVKPESTQGSENDASISDEHRNE
SGSSGGLSNQDGGSDNGSGTQSSWTKRASDTKSTSPSNQFPDAPNKKGTYENGCAHVNRLKE
AEDQKEQIGTGSQTGMSMSKKAEEPGDLEKNAKYSVQALERNNDDTLNRSSGNSQVESKAPS
SNREDLQSLEQTLKKTREDRDYKVGDRSVLRHSNLSAFSKYNNGATSAKKAPEENVESCSPH
DSPIAKLLGSSSSSDNPLKQQSSGSDRWAQREAALMKFRLKRKERCFEKKVRYHSRKKLAEQ
RPHVKGQFIRKRDDHKSGSEDN*
>APRR5
MTSSEEVVEVTVVKAPEAGGGKLSRRKIRKKDAGVDGLVKWERFLPKIALRVLLVEADDSTR
QIIAALLRKCSYRVAAVPDGLKAWEMLKGKPESVDLILTEVDLPSISGYALLTLIMEHDICKNIP
VIMMSTQDSVNTVYKCMLKGAADYLVKPLRRNELRNLWQHVWRRQTSLAPDSFPWNESVG
QQKAEGASANNSNGKRDDHVVSGNGGDAQSSCTRPEMEGESADVEVSARDAVQMECAKSQ
FNETRLLANELQSKQAEAIDFMGASFRRTGRRNREESVAQYESRIELDLSLRRPNASENQSSGD
RPSLHPSSASAFTRYVHRPLQTQCSASPVVTDQRKNVAASQDDNIVLMNQYNTSEPPPNAPRR
NDTSFYTGADSPGPPFSNQLNSWPGQSSYPTPTPINNIQFRDPNTAYTSAMAPASLSPSPSSVSP
HEYSSMFHPFNSKPEGLQDRDCSMDVDERRYVSSATEHSAIGNHIDQLIEKKNEDGYSLSVGKI
QQSLQREAALTKFRMKRKDRCYEKKVRYESRKKLAEQRPRIKGQFVRQVQSTQAP*
>APRR7
MNANEEGEGSRYPITDRKTGETKFDRVESRTEKHSEEEKTNGITMDVRNGSSGGLQIPLSQQT
AATVCWERFLHVRTIRVLLVENDDCTRYIVTALLRNCSYEVVEASNGIQAWKVLEDLNNHIDI
VLTEVIMPYLSGIGLLCKILNHKSRRNIPVIMMSSHDSMGLVFKCLSKGAVDFLVKPIRKNELKI

LWQHVWRRCQSSSGSGSESGTHQTQKSVKSKSIKKSDQDSGSSDENENGSIGLNASDGSSDGS
GAQSSWTKKAVDVDDSPRAVSLWDRVDSTCAQVVHSNPEFPSNQLVAPPAEKETQEHDDKF
EDVTMGRDLEISIRRNCDLALEPKDEPLSKTTGIMRQDNSFEKSSSKWKMKVGKGPLDLSSESP
SSKQMHEDGGSSFKAMSSHLQDNREPEAPNTHLKTLDTNEASVKISEELMHVEHSSKRHRGT
KDDGTLVRDDRNVLRRSEGSAFSRYNPASNANKISGGNLGSTSLQDNNSQDLIKKTEAAYDC
HSNMNESLPHNHRSHVGSNNFDMSSTTENNAFTKPGAPKVSSAGSSSVKHSSFQPLPCDHHNN
HASYNLVHVAERKKLPPQCGSSNVYNETIEGNNNTVNYSVNGSVSGSGHGSNGPYGSSNGMN
AGGMNMGSDNGAGKNGNGDGSGSGSGSGSGNLADENKISQREAALTKFRQKRKERCFRKKV
RYQSRKKLAEQRPRVRGQFVRKTAAATDDNDIKNIEDS*
>APRR9
MGEIVVLSSDDGMETIKNRVKSSEVVQWEKYLPKTVLRVLLVESDYSTRQIITALLRKCCYKV
VAVSDGLAAWEVLKEKSHNIDLILTELDLPSISGFALLALVMEHEACKNIPVIMMSSQDSIKMV
LKCMLRGAADYLIKPMRKNELKNLWQHVWRRLTLRDDPTAHAQSLPASQHNLEDTDETCED
SRYHSDQGSGAQAINYNGHNKLMENGKSVDERDEFKETFDVTMDLIGGIDKRPDSIYKDKSR
DECVGPELGLSLKRSCSVSFENQDESKHQKLSLSDASAFSRFEESKSAEKAVVALEESTSGEPK
TPTESHEKLRKVTSDQGSATTSSNQENIGSSSVSFRNQVLQSTVTNQKQDSPIPVESNREKAAS
KEVEAGSQSTNEGIAGQSSSTEKPKEEESAKQRWSRSQREAALMKFRLKRKDRCFDKKVRYQ
SRKKLAEQRPRVKGQFVRTVNSDASTKS*
>APRR2
MVITANDLSKWENFPKGLKVLLLLNGCDSDGDGSSAAETRSELESMDYIVTTFTDETEALSAV
VKNPESFHIAIVEVNMSAESESFKFLEAAKDVLPTIMISTDHCITTTMKCIALGAVEFLQKPLSPE
KLKNIWQHVVHKAFNDGGSNVSISLKPVKESVVSMLHLETDMTIEEKDPAPSTPQLKQDSRLL
DGDCQENINFSMENVNSSTEKDNMEDHQDIGESKSVDTTNRKLDDDKVVVKEERGDSEKEEE
GETGDLISEKTDSVDIHKKEDETKPINKSSGIKNVSGNKTSRKKVDWTPELHKKFVQAVEQLG
VDQAIPSRILELMKVGTLTRHNVASHLQKFRQHRKNILPKDDHNHRWIQSRENHRPNQRNYN
VFQQQHRPVMAYPVWGLPGVYPPGAIPPLWPPPLQSIGQPPPWHWKPPYPTVSGNAWGCPVG
PPVTGSYITPSNTTAGGFQYPNGAETGFKIMPASQPDEEMLDQVVKEAISKPWLPLPLGLKPPS
AESVLAELTRQGISAVPSSSCLINGSHRLR*
>APRR4
MQPLNMAEILDHRGVLTDGDDGPFRNLTNFYDMFSSNFPEGLRVLVFDEDPSYLLILERHLQK
FQYQVTICNEVNKAMHTLRNHRNRFDLAMIQVNNAEGDIFRFLSEIGSEMDLPIIIISEDDSVKS
VKKWMINGAADYLIKPIRPEDLRIVFKHLVKKMRERRSVVTGEAEKAAGEKSSSVGDSTIRNP
NKSKRSSCLEAEVNEEDRHDHNDRACASSAKKRRVVWDEELHQNFLNAVDFLGLERAVPKKI
LDVMKVDYISRENVASHLQVTFLIYNIIVHFQQHFCFYS*
>APRR6
MAGCLVPECADDISILLIDHDTASIASLTSMLQQFSKRVMSVDVASKALSMIEKQKKEIGLIIAN
IEMPHIDSHSFLNALLLKDIPLILINPEIKTKEPSDLLTKRACFSLDKPISNDDIKNMWQHVFSKK
SQELKKINITEDQENVMDKDTYQIEAFRANLKRQRISQASLLGRRPFIDTFSTYETFQKRKSIAN
VEWKTTPSYAIEIENKRKEWKKSVGRRKSLWNSERHMKFIAAISILGEEDFRPKSILEIMNDPN
LTHRQVGSHLQKYKAQIDQISYTLPRNESRSIDKTFEYPSNYKYPFKISDLTNNLIVSNSLWNSL
EKKNSASASITQFLFKKPIGEKEETMPKFHIGGKLDLSNHSVHGNVLNKLSMNVNFVPSTISNN
PAYNILSIDSSSIDSSSYTGLVSTGLSSENSPILYGLPSNDGASNTCTSQMESERISIPQYDPNQCH
PHRSILETDVNQIDLDFTSILDSFDPLVDECLMKENNRFLPNPTMNLLDTDIDKMDWVSFIENLS
HHDINMNHMDWDPSTANYVLPETNMNINFPEKHTNEIGWVSSQVGYVPFENMIPSEVDINHM

GMGYSGGSIPPQEETNTNNVGFVSCEIHSEIPPKTNMAILETNYSNPLDWVFPEDITSLETNTIQ
KSLVSCETSYDALDNMVPLETNMEEMNDALYDISIEDLISFDIDANEKDIFSWLEDNGFSEENN
MMESCEYHNIESVNQSDDMKIDDNFDDYRECMDWINEEMNKDV*
>APRR8
MIRKCGYKVVATTRADDLPLIINNKDKKIDLVLAEFRLIEMNKYELLEKIRSICEIPVVVSGAHV
KDAIVECLCRGAKLCLEKPLMENDFKILWQFTVSRQRNFRSQIDINPPEKNHSITHTQSLGAEL
KKNNNNSEVETEDLDKYKDELGQGNKRKERADTDTGEHTEKNNGSDLGDQKKPKLLFADDL
QNETLEAVPNIEEANNERKAPTEIKKNGESSEKKSPELVCMEEELQKWSAESFIDLTASVENES
QDPLESVGDSVGPHEIPLSPPESSNNNVAAQLQMPTHEALDEEVMQDGLSLSDLEVDLQEGHG
SNKELFDKIFTDLAKELKP*
