# Supplementary figures and images for: Genomic Insights into ARR Genes: Key Role in Cotton Leaf Abscission Formation
Source: Int J Mol Sci. 2025 Jul 24;26(15):7161. doi: 10.3390/ijms26157161 (PMC12346069; doi:10.3390/ijms26157161)

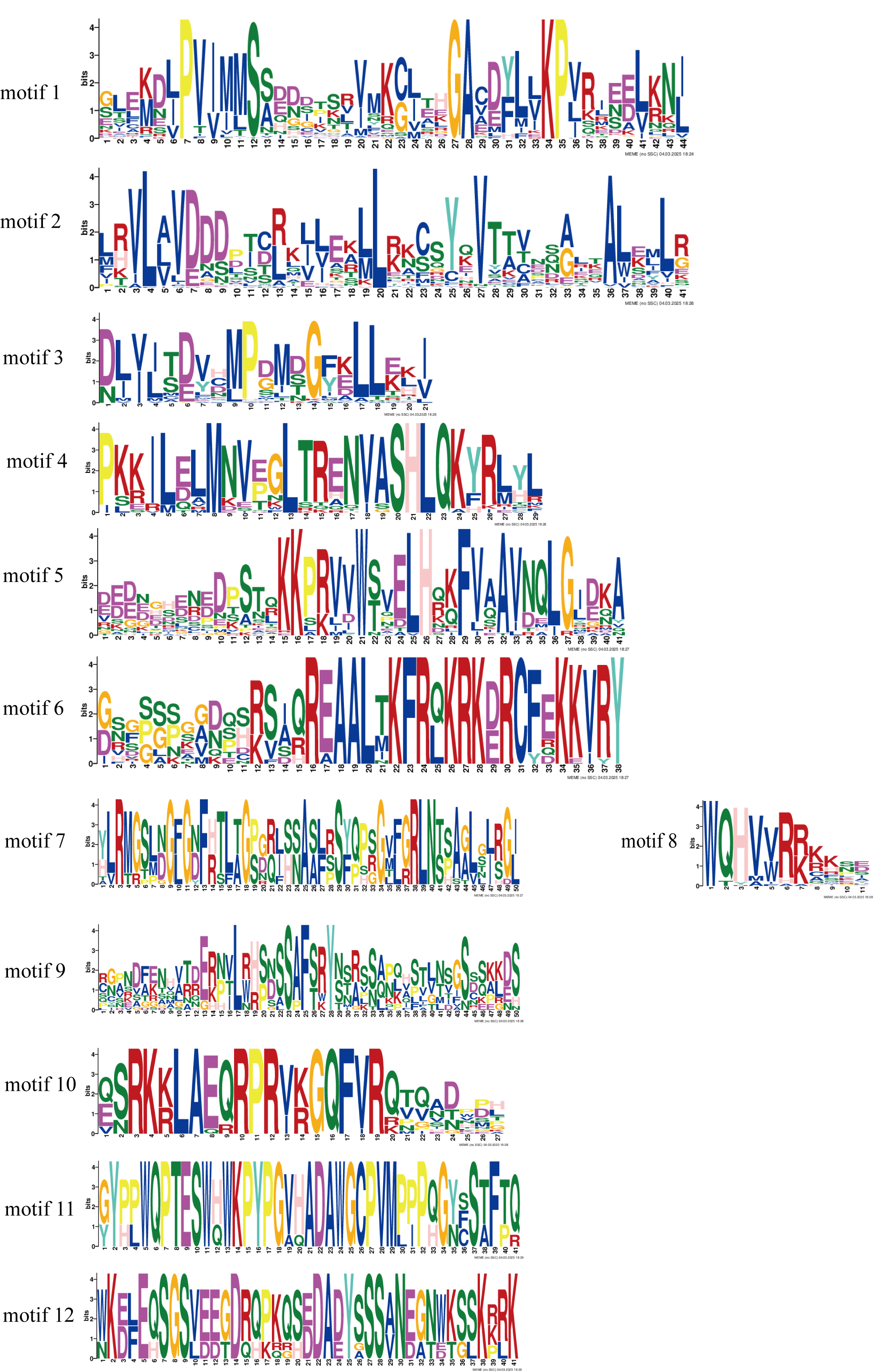

Supplement: Supplementary file 1 [file ijms-26-07161-s001.zip › Additional file S2.tif]
